# Dereplication and Chemotaxonomical Studies of Marine Algae of the Ochrophyta and Rhodophyta Phyla

**DOI:** 10.3390/md13052714

**Published:** 2015-04-30

**Authors:** Robert Brkljača, Emrehan Semih Gӧker, Sylvia Urban

**Affiliations:** School of Applied Sciences (Discipline of Chemistry), Health Innovations Research Institute (HIRi), RMIT University, GPO Box 2476V Melbourne, Victoria 3001, Australia; E-Mails: robert.brkljaca@rmit.edu.au (R.B.); emrehan_goker@hotmail.com (E.S.G.)

**Keywords:** ochrophyta, rhodophyta, dereplication, profiling, algae, HPLC-NMR, HPLC-MS

## Abstract

Dereplication and chemotaxonomic studies of six marine algae of the Ochrophyta and one of the Rhodophyta phyla resulted in the detection of 22 separate compounds. All 16 secondary metabolites, including four new compounds (**16**–**19**), could be rapidly dereplicated using HPLC-NMR and HPLC-MS methodologies in conjunction with the MarinLit database. This study highlights the advantages of using NMR data (acquired via HPLC-NMR) for database searching and for the overall dereplication of natural products.

## 1. Introduction

The Ochrophyta phylum contains in excess of 1800 species [1]. Recently, 17 new compounds, mostly terpenoids, were reported from the Ochrophyta phylum [2]. In contrast, marine algae belonging to the Rhodophyta phylum, are represented by over 6500 species [1] and produce a large number of halogenated secondary metabolites [3]. For example, nine new compounds were recently reported from the Rhodophyta phylum, a majority representative of bromophenols [2].

The intention of this study was to select a range of marine algae and conduct a dereplication/chemotaxonomical investigation as a means to rapidly differentiate the secondary metabolites present across different genera and between species of the same genera. For instance, marine algae of the Ochrophyta phylum such as *Cystophora retroflexa* have been reported to produce carotenoids, phlorethols and fucophlorethols [4,5,6] while *Cystophora subfarcinata* is known to produce tocotrienols and phloroglucinols [7]. The remaining Ochrophyta phylum genera and species investigated included, *Sargassum decipiens*, *Sargassum vestitum*, *Sargassum cf. fallax*, and *Halopteris pseudospicata* and importantly these have not had any marine secondary metabolites reported. However, the *Sargassum* genus is known to produce meroditerpenoid, tocotrienol and terpenoid type compounds [8]. The only marine alga of the Rhodophyta phylum studied was of the *Laurencia* genus which is known to produce very different secondary metabolites from the marine brown algae [9,10,11,12].

In this study, we examined six specimens of marine brown algae belonging to the Ochrophyta phylum (*Sargassum cf. fallax*, *Sargassum decipiens* (R.Brown ex Turner) J.Agardh, *Sargassum vestitum* (R.Brown ex Turner) C.Agardh, *Cystophora retroflexa* (Labillardière) J.Agardh, *Cystophora subfarcinata* (Mertens) J.Agardh *and Halopteris pseudospicata* Sauvageau) and one marine red alga from the Rhodophyta phylum (*Laurencia* sp.), all of which were collected from Port Phillip Bay, Victoria, Australia. These marine algae were selected for phytochemical evaluation on the basis of three criteria, but in all three instances the intention was to rapidly dereplicate the secondary metabolites present and to avoid lengthy isolations. The marine algae were either selected on the basis of the observed biological activity of the crude extracts, or due to the fact that no previous secondary metabolites had been described from the species of marine alga. The final motivation for the selection was based upon the fact that our research group has previously conducted studies on other closely related *Sargassum*, *Cystophora* and *Laurencia* species and so the intention was to compare the secondary metabolites in closely related species.

Herein, we report the chemical profiling/dereplication conducted using HPLC-NMR and HPLC-MS leading to the identification of seven different structure classes. In total, 22 compounds were detected in the dichloromethane crude extracts of the marine algae studied, of which 16 could be dereplicated.

## 2. Results and Discussion

The frozen marine algae were extracted with 3:1 methanol/dichloromethane, evaporated under reduced pressure and sequentially solvent partitioned (triturated) into dichloromethane and methanol soluble fractions, respectively. The dichloromethane and methanol crude extracts were initially analysed by off-line analytical HPLC and ^1^H-NMR analyses and this established that the majority of the secondary metabolites were present in the dichloromethane crude extracts. Based on this, only the dichloromethane extracts were further examined by chemical profiling methodologies (HPLC-NMR & HPLC-MS).

### 2.1. Chemical Profiling (HPLC-NMR & HPLC-MS)

The dichloromethane crude extracts were subjected to both HPLC-NMR and HPLC-MS chemical profiling and a total of 22 compounds were detected from the seven separate marine algae. Identical HPLC-NMR and HPLC-MS conditions were employed to probe the dichloromethane crude extracts of each alga to allow for comparison between each genera and/or species. Analysis of the stop-flow WET1D proton NMR spectra and extracted UV profiles for each of the compounds concluded the presence of seven distinct chemical structure classes including phenolic acids, phenols, resorcinols, phloroglucinols, xanthophylls, tocotrienols and C_15_ halogenated acetogenins, which are known to occur in these genera or species of algae [6,7,8,13,14,15,16,17].

**Figure 1:**
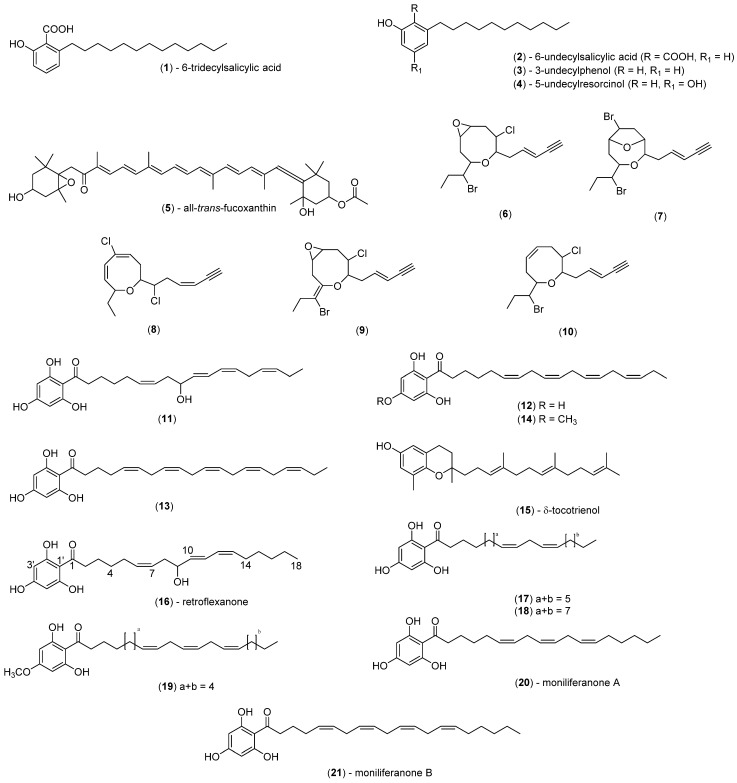
Compounds and range of structure classes as dereplicated by HPLC-NMR and HPLC-MS in conjunction with the MarinLit database.

Twelve known (**1**–**5**, **11**–**15**, **20** and **21**) and four new (**16**–**19**) compounds (Figure 1) were dereplicated from the dichloromethane crude extracts of the algae. Compounds were dereplicated by analysis of the HPLC-NMR acquired data (WET1D and various combinations of gCOSY, HSQCAD, and gHMBCAD), high resolution HPLC-MS data, and use of the MarinLit database by searching parameters such as the taxonomy (usually genus), UV, MS and NMR data. While some structure classes are unique to certain genera or species, some can be present across various genera or species of algae (Table 1). For instance, in this study, phloroglucinols were detected in *S. cf. fallax*, *C. subfarcinata* and *C. retroflexa* while tocotrienols were exclusive to *S. cf. fallax*. The xanthophylls, which are known to occur in many marine brown algae [8,18], were found in high abundance in *S. vestitum* and *H. pseudospicata*. The specimen of *S. decipiens* was concluded to produce phenolic acids, phenols, and resorcinols while the specimen of *Laurencia* sp. could be deduced as containing C_15_ halogenated acetogenins. Table 2 summarises each of the components detected in the seven marine algae studied together with the search criteria used to dereplicate the structures present in the dichloromethane crude extracts. The amount of compound present in each of the crude extracts was estimated on the basis of the limit of detection (LOD) methodology recently reported for our HPLC-NMR system [19]. In this study, the LOD for five key NMR experiments was established for a given set of parameters. These LODs and the parameters utilized were reviewed, and on this basis, the approximate amount of each compound present in the crude extract for each of the HPLC-NMR analyses undertaken was estimated (see Table 2). In HPLC-NMR analyses, it is imperative to supress signals arising from the HPLC solvents (HDO signal arising from D_2_O and the CH_3_CN peak) in order to maximise signal intensity and obtain better quality NMR spectra. Unfortunately, during this process NMR signals of the compound of interest which occur within this suppression region are also inadvertently suppressed.

**Table 1:** Chemotaxonomic comparison of the seven marine algae studied and the chemical classes present in each.

Alga	Chemical Class(es) Present	Compounds Present *
*C. subfarcinata*	Phloroglucinols	**18**, **19**
*C. retroflexa*	Phloroglucinols	**11**-**13**, **16**, **18**, **20**, **21**
*S. cf. fallax*	Phlroglucinols, tocotrienols	**12**, **14**, **15**, **17**, **20**, **21**
*S. decipiens*	Phenols, phenolic acids, resorcinols	**1**–**4**
*S. vestitum*	Xanthophylls	**5**
*H. pseudospicata*	Xanthophylls	**5**
*Laurencia* sp.	Polyhalogenated C_15_ acetogenins	-

* Retention times for compounds present are provided in Table 2.

**Table 2:** Identification of chemical structure classes present in seven marine algae studied (ordered on the basis of HPLC-NMR retention time, *R*_t_).

Peak #	*R_t_* (min)	Compound	Structure Class	Marine Alga (~Amount Present in μg)	UV (nm)	MarinLit Search Parameters	New/Known
1	2.29	(**2**)	Phenolic acid	*S. decipiens* (500–1000)	240, 302	Compound not in MarinLit database	Known
2	2.44	(**3**)	Phenol	*S. decipiens* (500–1000)	236, 301	Compound not in MarinLit database	Known
3	3.42	(**11**)	Phloroglucinol	*C. retroflexa* (750–1000)	235, 285	Molecular formula, UV ± 5	Known
4	3.55	(**1**)	Phenolic acid	*S. decipiens* (<100)	235, 301	Molecular formula, UV ± 10	Known
5	4.45	(**16**)	Phloroglucinol	*C. retroflexa* (750–1000)	235, 285	Compound not in MarinLit database	New
6	5.00	n.a.	C_15_ acetogenin	*Laurencia* sp. (<25)	220, 237	Genus, UV ± 5, 1 triplet methyl group	Not Identified
7	6.05	n.a.	Unknown	*Laurencia* sp. (500–1000)	220, 240, 255	Unable to dereplicate using any parameters	Not Identified
8	6.70	n.a.	C_15_ acetogenin	*Laurencia* sp. (<25)	220, 237	Genus, UV ± 5, 1 triplet methyl group	Not Identified
9	7.87	(**4**)	Resorcinol	*S. decipiens* (<10)	229, 276, 281	Compound not in MarinLit database	Known
10	9.98	(**12**)	Phloroglucinol	*C. retroflexa* (500–1000), *S. cf. fallax* (<250)	230, 285	Molecular formula, UV ± 5	Known
11	12.95	(**13**)	Phloroglucinol	*C. retroflexa* (250–750)	230, 285	Molecular formula, UV ± 5	Known
12	13.65	(**17**)	Phloroglucinol	*S. cf. fallax* (<250)	215, 228, 285	Compound not in MarinLit database	New
13	14.53	(**5**)	Xanthophyll	*S. vestitum* (<100), *H. pseudospicata* (<100)	450	Molecular formula	Known
14	15.50	(**20**)	Phloroglucinol	*C. retroflexa* (<500), *S. cf. fallax* (<100)	212, 228, 285	Compound not in MarinLit database	Known [13]
15	20.15	(**21**)	Phloroglucinol	*C. retroflexa* (<250), *S. cf. fallax* (<50)	230, 285	Compound not in MarinLit database	Known [13]
16	21.62	(**14**)	Phloroglucinol	*S. cf. fallax* (<100)	212, 228, 285	Molecular formula, UV ± 5	Known
17	22.96	(**18**)	Phloroglucinol	*C. retroflexa* (<100), *C. subfarcinata* (<50)	228, 285	Compound not in MarinLit database	New
18	23.16	n.a.	Phloroglucinol	*C. retroflexa* (<100)	238, 288	Genus, UV ± 5	Not Identified
19	26.71	n.a.	Xanthophyll	*S. vestitum* (<5), *H. pseudospicata* (<5)	450	Insufficient data to search MarinLit Database	Not Identified
20	30.27	n.a.	Xanthophyll	*S. vestitum* (<5), *H. pseudospicata* (<5)	450	Insufficient data to search MarinLit Database	Not Identified
21	33.40	(**19**)	Phloroglucinol	*C. subfarcinata* (<50)	213, 228, 285	Compound not in MarinLit database	New
22	60.80	(**15**)	Tocotrienol	*S. cf. fallax* (<10)	212, 300	Class, UV ± 5, contains only singlet aromatic/vinyl CH_3_ groups, aromatic ring	Known

n.a. indicates that a structure could not be concluded.

### 2.2. Identification of Phenols, Phenolic Acids and Resorcinols

The specimen of *S. decipiens* was concluded to contain compounds **1**–**4**, which represent three different structure classes (phenols, phenolic acids and resorcinols). While the phenols and phenolic acids displayed similar UV absorbances (300 nm), the resorcinol exhibited unique UV absorbances (276 and 281 nm). The compound eluting at *R*_t_ = 3.55 min was dereplicated from the MarinLit database using the molecular formula and UV absorbances as search parameters. This yielded four possible compounds, but analysis of the NMR data obtained from HPLC-NMR supported only one of these structures. In this way, the compound eluting at *R*_t_ = 3.55 min could be identified as 6-tridecylsalicylic acid (**1**) which had been previously reported from the marine brown alga *Caulocystis cephalornithos* [20].

The remaining compounds eluting at *R*_t_ = 2.29, 2.44 and 7.87 min did not yield any matches in the MarinLit database when using various combinations of UV, mass and molecular formulae, and, when only UV data was searched this resulted in far too many possible structure classes. On the basis of the WET1D NMR spectra and UV profiles of the remaining three compounds (*R*_t_ = 2.29, 2.44 and 7.87 min) it could be concluded that they were closely related to 6-tridecylsalicylic acid (**1**). The marine brown alga *C. cephalornithos*, which is known to produce 6-tridecylsalicylic acid (**1**), also produces a variety of structurally related analogues [20,21]. Comparison of the data obtained from HPLC-NMR and HPLC-MS to the data for the compounds reported from *C. cephalornithos*, allowed for the compounds eluting at *R*_t_ = 2.29, 2.44 and 7.87 min to be identified as undecylsalicylic acid (**2**), 3-undecylphenol (**3**) and 5-undecylresorcinol (**4**) respectively. It is important to point out that these compounds do not appear separately in the MarinLit database and care must be taken in such dereplication exercises. This is the first study conducted on *S. decipiens* and compounds **1**–**4** have not been reported from the *Sargassum* genus. This highlights the importance of profiling different genera and species as a means of extending knowledge on marine biodiversity. The HPLC-NMR and HPLC-MS data of the compounds identified were consistent with the data reported previously [20,21,22,23].

### 2.3. Identification of Xanthophylls

**Figure 2:**
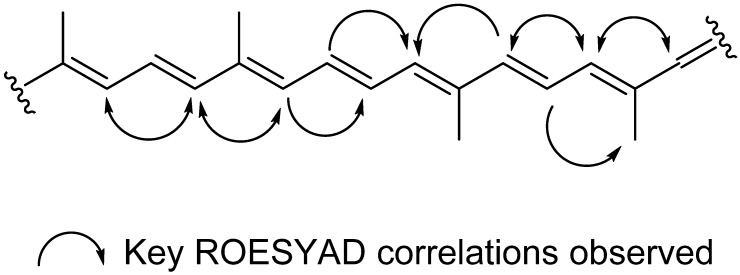
key ROESYAD correlations confirming the all *trans* configuration in all-*trans*-fucoxanthin (**5**).

The dichloromethane crude extracts of *S. vestitum* and *H. pseudospicata* were found to contain an abundance of xanthophylls. The main compound in the dichloromethane crude extract of each of these two algae (compound eluting at *R*_t_ = 14.53 min) was identified as all-*trans*-fucoxanthin (**5**), the main pigment found in marine brown algae. This pigment was identified using the MarinLit database by searching the molecular formula obtained by high resolution HPLC-MS, which yielded only one structure. The UV profile, WET1D, gCOSY and HSQCAD NMR spectra of **5** were found to be in agreement with the structure of all-*trans*-fucoxanthin [24,25] A ROESYAD NMR experiment conducted in stop-flow HPLC-NMR mode confirmed that the configuration for all of the double bonds was *trans* (Figure 2).

**Figure 3:**
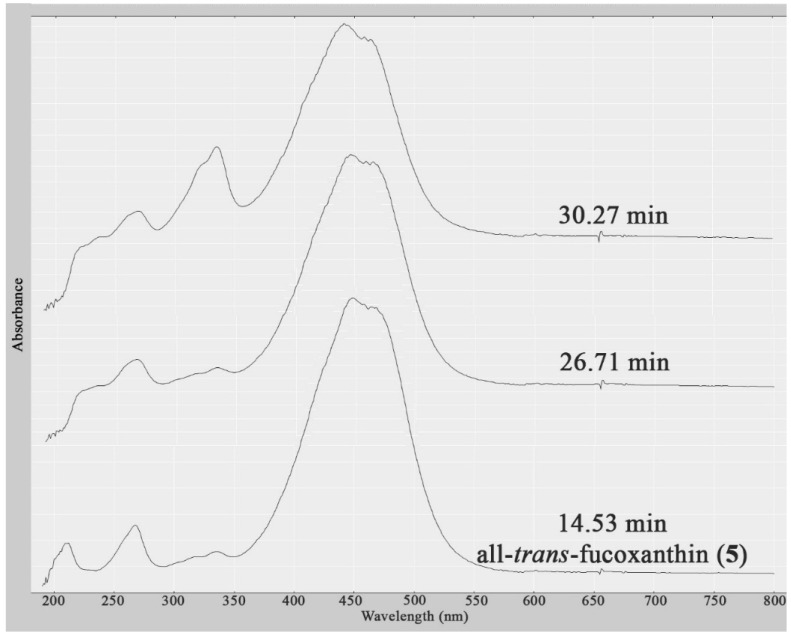
UV profiles (obtained from HPLC-NMR) of the three related xanthophylls present in the dichloromethane crude extracts of *H. pseudospicata and S. vestitum* showing their similarity.

A further two structurally related compounds eluting at *R*_t_ = 26.71 and 30.27 min were also detected in the dichloromethane crude extract of both *S. vestitum* and *H. pseudospicata*, but in much lower quantities. Identification was not possible due to the insufficient quantities present to obtain HPLC-NMR data, and due to the fact that the compounds did not ionize in either positive or negative ESI high resolution HPLC-MS. However, these compounds could be deduced to be two xanthophyll structural analogues based upon comparison of their UV profiles to all-*trans*-fucoxanthin (**5**) (Figure 3). This is the first report of all-*trans*-fucoxanthin (**5**) and other xanthophylls occurring in the *Halopteris* genus [8].

### 2.4. Identification of Halogenated C_15_ Acetogenins

**Figure 4:**
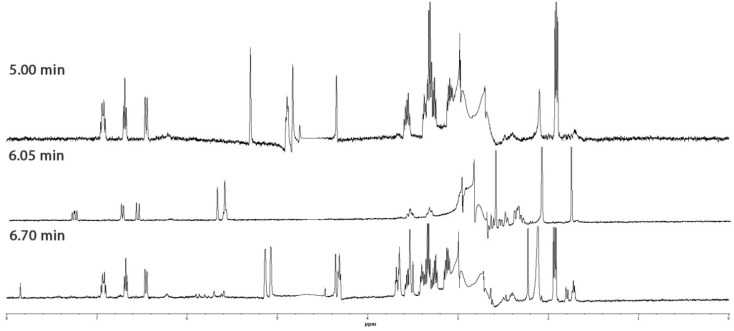
WET1D NMR spectra (75% CH_3_CN/D_2_O, 500 MHz) of compounds eluting at *R*_t_ = 5.00, 6.05 and 6.70 min from the dichloromethane crude extract of *Laurencia* sp.

Profiling of a specimen of *Laurencia* sp. yielded three compounds (eluting at *R*_t_ = 5.00, 6.05 and 6.70 min), which displayed similar WET1D NMR spectra (Figure 4) confirming that they were structurally related. WET1D and gCOSY NMR data were obtained for the compounds eluting at *R*_t_ = 5.00 and 6.70 min, while WET1D, gCOSY, HSQCAD and gHMBCAD NMR data was obtained for the compound eluting at *R*_t_ = 6.05 min.

Based upon the HPLC-NMR WET1D spectra (Figure 4), it was clear that the compounds eluting at *R*_t_ = 5.00 and 6.70 min were structural analogues, whereas the compound eluting at *R*_t_ = 6.05 min was a closely related structural derivative. A MarinLit search was conducted for the compound eluting at *R*_t_ = 5.00 min using the genus, UV and the presence of a single triplet methyl in the WET1D proton NMR spectrum obtained from stop-flow HPLC-NMR. This yielded nine possible structures including five polyhalogenated C_15_ acetogenins (**6**–**10**) (Figure 1). The WET1D NMR data for the compound eluting at *R*_t_ = 5.00 min supported the presence of olefinic protons (δ 6.93, dt, *J* = 7.5, 10.0 Hz; 6.69, t, *J* = 8.0 Hz; 6.46, d, *J* = 10.0 Hz), and the presence of halogens was deduced based on the presence of several deshielded methines (δ 5.30, s; 4.90, m; 4.84, s; 4.76, s; 4.35, s). Unfortunately, due to suppression of the methine signals in the D_2_O suppression region, it was not possible to confirm the total number of halogens present. Furthermore, the compound eluting at *R*_t_ = 5.00 min did not ionize in either positive or negative ESI high resolution HPLC-MS, making it impossible to determine a molecular formula. It could only be proposed that the compounds eluting at *R*_t_ = 5.00 min and *R*_t_ = 6.70 min are of the polyhalogenated C_15_ acetogenin structure class, but the exact nature of the ring size could not be concluded.

The WET1D NMR spectrum for the compound eluting at *R*_t_ = 6.05 min showed slight differences suggesting it was a structural variant, further supported by the UV profile (220, 237 nm for the compounds eluting at *R*_t_ = 5.00 and 6.70 min), which also differed, and the absence of any absorbances in the UV profile suggested very little conjugation (220, 240 and 255 nm). Only partial HSQCAD and gHMBCAD NMR data could be obtained for this component and this hindered the ability to dereplicate the compound. Similar to the compounds eluting at *R*_t_ = 5.00 and 6.70 min, the compound eluting at *R*_t_ = 6.05 min did not ionize in ether positive or negative ESI high resolution HPLC-MS. Various searches conducted of the MarinLit database using different combinations of data (taxonomy, UV and NMR) did not aid in dereplicating a general structure class, and so the compound eluting at *R*_t_ = 6.05 min remains unidentified. Off-line isolation would be necessary to unequivocally deduce the structures of these three compounds.

### 2.5. Identification of Phloroglucinols and Tocotrienols

The dichloromethane crude extracts of *S. cf. fallax*, *C. subfarcinata* and *C. retroflexa* yielded a total of eleven phloroglucinols (**11**–**14**, **16**–**21**) and one tocotrienol (**15**). These two structure classes display similar UV absorbances, with the phloroglucinols typically displaying an absorbance at 285 nm and the tocotrienols at 300 nm, however, differences are observed in the WET1D NMR spectra. This represents the first instance of phloroglucinols and tocotrienols being reported from *C. retroflexa* and *S. cf. fallax*. The known phloroglucinols (**11**–**14**) and tocotrienol (**15**) were all dereplicated with use of the MarinLit database utilising the search parameters as indicated in Table 2, and by comparison of the NMR data obtained by HPLC-NMR. One of the phloroglucinols (compound eluting at *R*_t_ = 23.16 min) could not be identified due to insufficient quantities present to permit sufficient HPLC-NMR data acquisition and also due to there being no observed molecular ion in the high resolution HPLC-MS. However, based on the similarities of the UV absorbances and WET1D NMR data to **11**, it was concluded to represent a phloroglucinol containing an alcohol substituent in the terpene side chain. δ-tocotrienol (**15**) did not ionize in the high resolution HPLC-MS analysis, but a search of the MarinLit database together with consideration of the WET1D NMR data, resulted in δ-tocotrienol (**15**) being identified. The HPLC-NMR and HPLC-MS data of the known compounds was consistent with the data previously reported [26,27,28,29].

As well as compounds **11**–**15**, a series of new phloroglucinols were also determined. A MarinLit search conducted on these phloroglucinols using combinations of taxonomy, UV, molecular formulae and NMR data did not result in any confirmed structures, supporting the fact that these represented new phloroglucinols.

High resolution HPLC-MS yielded a molecular formula of C_24_H_34_O_5_ for **16** (observed 401.2337 [M − H]^−^, calcd. for C_24_H_33_O_5_, 401.2328) and stop-flow HPLC-NMR enabled for WET1D proton, gCOSY, HSQCAD and gHMBCAD NMR data to be obtained (Table 3). Three double bonds were concluded to be present based on the degrees of unsaturation and inspection of the WET1D NMR spectrum (δ_H_ 7.30, 1H; 6.80, 1H; 6.48, 1H; 6.32, 1H; 6.28, 1H; 6.23, 1H). A diagnostic methine at δ_H_ 4.96 was observed which suggested the presence of an alcohol substituent, together with a diagnostic methylene at δ_H_ 3.89, which is typical of the methylene group attached adjacent to the ketone functionality. The gCOSY and gHMBCAD NMR data was able to secure the spin system from this diagnostic methylene (δ_H_ 3.89, H-2) to the first olefinic proton (δ_H_ 6.32, H-6). Further interpretation of the gCOSY NMR data secured the spin system from H-6 (δ_H_ 6.32) to H-14 (δ_H_ 3.00). The proton at position H-14 lies on the edge of the suppression region of the acetonitrile signal, which prevented any gCOSY NMR correlations from the proton being observed. Fortunately, in the gHMBCAD NMR spectrum, key correlations were observed from this methylene which allowed for the carbon at position C-15 to be assigned (δ_C_ 29.9). The terminal methyl group (δ_H_ 1.72, H-18) displayed a key gHMBCAD NMR correlation to the carbon at position C-16 (δ_C_ 32.0), confirming its assignment. The protons at positions H-5, H-15 and H-16 could not be assigned as these signals resided in the acetonitrile suppression region. The configuration of the double bonds was established by consideration of the NMR coupling constants and on biosynthetic grounds. Compound **16** represents a structure analogue of the known phloroglucinol **11**, which is known to have a 6*Z* configuration [28].The co-occurrence of **11** and **16** led to the conclusion that their double bond configuration about position 6/7 would be the same when considering their biosynthesis. As such, the structure for **16** was proposed as 9-hydroxy-1-(2,4,6-trihydroxyphenyl)octadeca-6*Z*,10*E*,12*Z*-trien-1-one, attributed the trivial name retroflexanone (**16**). The configuration of the single stereogenic center at position C-9 remains undefined.

**Table 3:** NMR data (500 MHz, 75% CH_3_CN/D_2_O, suppression of HDO and CH_3_CN at δ_H_ 4.64 and 2.82 ppm, respectively) for retroflexanone (**16**) obtained via stop-flow HPLC-NMR.

Retroflexanone (16)				
Position	δ_H_ (*J* in Hz)	δ_C_^a^_,_ Type	gCOSY	gHMBCAD
1		207.2, s		
2	3.89, t (7.0)	44.2, t	3	1, 3, 4
3	2.48, p (7.0)	25.1, t	2, 4	1, 2, 4, 5
4	2.23, m	29.9, t	3	2, 3, 5, 6
5	SS	27.8, t		
6	6.32, m	132.6, d	7	
7	6.23, m	126.2, d	6, 8	
8	3.12, m	35.8, t	7 ^w^, 9	6, 7, 9
9	4.96, dt (6.5, 7.0)	72.6, d	8, 10	11
10	6.48, dd (7.0, 15.0)	136.6, d	9, 11	12
11	7.30, dd (15.0, 11.0)	126.4, d	10, 12	
12	6.80, t (11.0)	128.8, d	11, 13 ^w^	
13	6.28, m	133.3, d	12, 14	
14	3.00, m ^b^	ND		12, 13, 15
15	SS	29.9, t		
16	SS	32.0, t		
17	2.13, m	23.0, t	18	16
18	1.72, t (6.5)	14.3, q	17	16, 17
1′		105.2, s		
2′		165.0, s *		
3′	6.72, s	95.7, d		1′, 2′, 5′
4′		ND *		
5′	6.72, s	95.7, d		1′, 3′, 6′
6′		165.0, s *		
2′-OH	ND			
4′-OH	ND			
6′-OH	ND			

^a^ carbon assignments based on HSQCAD and gHMBCAD NMR experiments; ^b^ signal assigned based on correlations in gCoSY experiment; * signals for C-2′ and C-6′are interchangeable with C4′; ^w^ indicates weak or long range correlation; SS Signal suppressed; ND Not Detected.

Based upon the molecular formula of the phloroglucinols eluting at *R*_t_ = 13.65, 22.96 and 33.40 min, the number of double bonds present in each compound could be concluded. Unfortunately, NMR and MS data alone is not sufficient to determine the placement of the double bond within the structures. This meant that only *tentative* structures could be proposed, which are represented as **17**–**19**, respectively. To establish the position of the double bonds in the terpene side chain, off-line approaches such as chemical derivatization or degradation are required. The double bonds were assigned on the basis that olefinic protons that correspond to a *cis* geometry appear as a narrow multiplet in the proton NMR spectrum [28] which was also observed for the known compounds (**12**–**14**) during this study.

Phloroglucinols **20** and **21** (compounds eluting at *R*_t_ = 20.15 and 15.50 min) do not appear in the MarinLit database, however, a recent study conducted by the research group describes the first report of these phloroglucinols occurring in *C. monilifera* and *C*. *subfarcinata* [13].

### 2.6. Anti-Microbial Activity

The crude extracts of *S. cf. fallax*, *S. vestitum*, *C. subfarcinata*, *H. pseudospicata* and *C. retroflexa* were assessed for their anti-microbial activity against six bacteria and three fungi (Table 4). The crude extracts of the algae showed varying selectivity and activity across the micro-organisms tested, with *S. vestitum*, *C. subfarcinata* and *C. retroflexa* showing the most promising activity. The crude extracts of the two marine algae specimens containing the xanthophylls (*S. vestitum* and *H. pseudospicata*) displayed different biological activity suggesting that the active component(s) was unlikely to be the xanthophylls but other chemical component(s). However, all-*trans*-fucoxanthin (**5**) has been reported to display anti-inflammatory, anti-oxidant and anti-tumor activity [30,31,32,33]. The crude extracts of *S. cf. fallax*, *C. subfarcinata*, *C. retroflexa* all showed similar anti-microbial activities which can potentially be attributed to the presence of the phloroglucinols. Phloroglucinols have been demonstrated to possess various anti-microbial activities [34,35,36]. The crude extracts of *S. decipiens* and *Laurencia* sp. were not evaluated for their biological activity.

## 3. Experimental Section

### 3.1. Marine Alga Material

*S. cf. fallax* was collected at low tide on January 3, 2003 from St Paul’s Beach, Sorrento, Port Phillip Bay, Victoria, Australia. *C. subfarcinata*, *S. vestitum* and *H. pseudospicata* were collected at low tide on January 22, 2006 from the Borough of Queenscliff (near Point Lonsdale), Port Phillip Bay, Victoria, Australia. *Laurencia* sp., *S. decipiens* and *C. retroflexa* were collected by SCUBA on 21 April 2010 from Governor Reef (near Indented Head), Port Phillip Bay, Victoria, Australia. The marine algae were identified by Dr. Gerald Kraft (The University of Melbourne) and voucher specimens (designated the code numbers 2003-06, 2006-11, 2006-12, 2006-13, 2010-04, 2010-08 and 2010-09, respectively) are deposited at the School of Applied Sciences (Discipline of Applied Chemistry), RMIT University, Melbourne, Australia.

### 3.2. Extraction

Each of the frozen marine algae were extracted with 3:1 methanol/dichloromethane (1 L). The crude extracts were then decanted and concentrated under reduced pressure and sequentially solvent partitioned (triturated) into dichloromethane and methanol soluble extracts, respectively.

**Table 4:** Anti-microbial activity of the crude extracts obtained from *S. cf. fallax*, *C. subfarcinata*, *C. retroflexa*, *S. vestitum* and *H. pseudospicata* showing zones of inhibition (mm).

Crude	Microorganism Concentration (mg/mL)	*E. coli* ATCC 25922	*S. aureus* ATCC 25923	*S. aureus MRSA* 344/2-32	*P. aeruginosa* ATCC 27853	*S. pyogenes* 345/1	*B. subtilis ATCC 19659*	*C. albicans* ATCC 10231 or 14053*	*T. mentagrophytes ATCC 28185*	*C. resinae*
***S. vestitum* (3:1 MeOH/DCM)**	50	ND ^a^	NT ^b^	NT ^b^	3	NT ^b^	ND ^a^	3 *	2	ND ^a^
***H. pseudospicata* (3:1 MeOH/DCM)**	50	ND ^a^	NT ^b^	NT ^b^	ND ^a^	NT ^b^	ND ^a^	ND ^a^	ND ^a^	ND ^a^
***S. cf. fallax* (3:1 MeOH/DCM)**	50	ND ^a^	NT ^b^	NT ^b^	ND ^a^	NT ^b^	2	ND ^a^*	ND ^a^	ND ^a^
***C. subfarcinata* (3:1 MeOH/DCM)**	50	ND ^a^	NT ^b^	NT ^b^	1	NT ^b^	ND ^a^	3*	ND ^a^	ND ^a^
***C. retroflexa* (DCM)**	50	ND ^a^	2	3	5	ND ^a^	NT ^b^	ND ^a^	NT ^b^	NT ^b^
***C. retroflexa* (MeOH)**	50	1	4	6	1	ND ^a^	NT ^b^	ND ^a^	NT ^b^	NT ^b^

^a^ indicates no zone of inhibition detected; ^b^ indicates not tested; * indicates tested against ATCC 14053. Please note: S. *decipiens* and *Laurencia* sp. were not evaluated for biological activity.

### 3.3. Biological Evaluation

For details on the biological evaluation of the dichloromethane and methanol crude extracts of *C. retroflexa* please refer to [37]. The 3:1 MeOH/DCM crude extracts of *S. cf. fallax*, *C. subfarcinata*, *S. vestitum* and *H. pseudospicata* were assessed for anti-microbial activity at the University of Canterbury, Christchurch, New Zealand. A standardized inoculum was prepared by transferring a loop of bacterial/fungal cells, from a freshly grown stock slant culture, into a 10 mL vial of sterile water. This was vortexed and compared to a 5% BaCl_2_ in water standard to standardize the cell density. This gave a cell density of 108 colony-forming units per millilitre. Ten millilitres of the standardized inoculum was then added to 100 mL of Mueller Hinton or potato dextrose agar (at between 40 and 50 °C) and mixed by swirling, giving a final cell density of 107 colony forming units per millilitre. Five millilitres of this was poured into sterile 85 mm petri dishes. The suspensions were allowed to cool and solidify on a level surface to give a “lawn” of bacteria/fungi over the dish. The crude extract was pipetted onto 6 millimeter diameter filter paper disks and the solvent evaporated. These disks were then placed onto the prepared seeded agar dishes (with appropriate solvent and positive controls) and incubated. Active anti-microbial samples displayed a zone of inhibition outside the disk, which was measured in mm as the radius of inhibition for each bacteria/fungi. The six organisms were *Eschericha coli* (ATCC 25922), *Bacillus subtilis* (ATCC 19659) and *Pseudomonas aeruginosa* (ATCC27853) for the bacteria and, *Candida albicans* (ATCC 14053), *Trichophyton mentagrophytes* (ATCC 28185) and *Cladosporium resinae* for the fungi.

### 3.4. Chemical Profiling

Chemical profiling was carried out on the dichloromethane soluble extracts of the marine algae employing HPLC-NMR and HPLC-MS methodologies. The dichloromethane extracts were dissolved in HPLC-NMR grade CH_3_CN and filtered through a 0.45 PTFE membrane filter (Grace Davison Discovery Sciences, Columbia, MD, USA). *S. decipiens*: 109.1 mg dissolved in 1090 μL (5005 μg per HPLC-NMR injection); *C. retroflexa*: 185.6 mg dissolved in 2230 μL (4161 μg per HPLC-NMR injection); *S. cf. fallax*: 166.5 mg dissolved in 1665 μL (5000 μg per HPLC-NMR injection); *S. vestitum*: 342.8 mg dissolved in 3430 μL (4997 μg per HPLC-NMR injection); *H. pseudospicata*: 61.6 mg dissolved in 615 μL (5008 μg per HPLC-NMR injection); *Laurencia* sp.: 216.2 mg dissolved in 2160 μL (5005 μg per HPLC-NMR injection); *C. subfarcinata*: 741.9 mg dissolved in 7420 μL (4999 μg per HPLC-NMR injection).

### 3.5. HPLC-NMR & HPLC-MS Conditions

For details of the HPLC-NMR conditions please refer to [37]. For both on-flow and stop-flow HPLC-NMR modes, 50 μL injections of the dichloromethane extracts were injected onto an Agilent Eclipse Plus C_18_ (150 × 4.6) 5 μ column using a solvent composition of 75% CH_3_CN/D_2_O at a flow rate of 1 mL/min. During HPLC-NMR analyses, the HDO and CH_3_CN signals are suppressed at 4.64 and 2.82 ppm, respectively. In the stop-flow HPLC-NMR mode, WET1D, gCOSY, HSQCAD and gHMBCAD NMR experiments were acquired. HRESILCMS was carried out on an Agilent 6540 Series TOF system in either the positive or negative ionization mode (ESI operation conditions of 12 L/min N2, 325 °C drying gas temperature, and 3500 V capillary voltage) equipped with an Agilent 1260 Infinity Binary Pump, Agilent 1290 Infinity Autosampler and Agilent 1260 DAD detector (Agilent, Santa Clara, CA, USA). The instrument was calibrated using the “Agilent Tuning Mix” with purine as the reference compound and the Hewlett-Packard standard HP0921. The separations were carried out using an Agilent Eclipse Plus C_18_ (4.6 × 150) 5 μ column using a solvent composition of 75% CH_3_CN/H_2_O at a flow rate of 1 mL/min.

### 3.6. On-Line (HPLC-NMR & HPLC-MS) Partial Characterization of Compounds

1-(2,4,6-trihydroxyphenyl)-hexadecadien-1-one (unknown double bond positions) (**17**): HPLC-NMR WET1D NMR (500 MHz, 75% CH_3_CN/D_2_O, suppression of HDO and CH_3_CN at t δ_H_ 4.64 and 2.82 ppm, respectively) obtained from stop-flow mode δ 6.71 (2H, s, H-3′/H-5′), 6.22 (4H, m, C**H**C**H**), 3.89 (2H, t, *J* = 8.0, H-2), 3.62 (2H, m, CHC**H**_2_CH), 2.11 (m, CH_2_)*, 1.77 (3H, t, *J* = 8.0; HRESILCMS; *m*/*z* 359.2226 (calcd for C_22_H_31_O_4_, 359.2222).

1-(2,4,6-trihydroxyphenyl)-octadecadien-1-one (unknown double bond positions) (**18**): HPLC-NMR WET1D NMR (500 MHz, 75% CH_3_CN/D_2_O, suppression of HDO and CH_3_CN at t δ_H_ 4.64 and 2.82 ppm, respectively) obtained from stop-flow mode δ 6.70 (2H, s, H-3’/H-5’), 6.20 (4H, m, C**H**C**H**), 3.87 (2H, t, *J* = 7.5, H-2), 3.61 (2H, dd, *J* = 6.0, 7 Hz, CHC**H**_2_CH), 2.46 (2H, m, H-3), 2.11 (m, CH_2_)*, 1.71 (3H, t, *J* = 7.0; HRESILCMS; *m*/*z* 387.2539 (calcd for C_24_H_35_O_4_, 387.2535).

1-(2,6-dihydroxy-4-methoxyphenyl)-octadecatrien-1-one (unknown double bond positions) (**19**): HPLC-NMR WET1D NMR (500 MHz, 75% CH_3_CN/D_2_O, suppression of HDO and CH_3_CN at t δ_H_ 4.64 and 2.82 ppm, respectively) obtained from stop-flow mode δ 6.82 (2H, s, H-3’/H-5’), 6.21 (6H, m, C**H**C**H**), 3.91 (2H, t, *J* = 7.0, H-2), 3.66 (4H, m, CHC**H**_2_CH), 2.49 (2H, m, H-3), 2.25 (2H, m, H-4), 2.10, (m, CH_2_)*, 1.70 (3H, t, *J* = 6.5; HRESILCMS; *m*/*z* 399.2539 (calcd for C_25_H_35_O_4_, 399.2535).

Please refer to the supplementary information file containing UV profiles, HPLC-NMR data including tabulated NMR data and the high resolution HPLC-MS chromatograms for all remaining compounds (these are listed in order of retention time).

## 4. Conclusions

This study has demonstrated the importance and ability of HPLC-NMR in conjunction with high resolution HPLC-MS and databases such as MarinLit to rapidly dereplicate the identity of secondary metabolites from a range of marine algae. The chemical diversity between, and within, marine algae genera was highlighted (See Table 1 and Table 2). The phloroglucinols (**11**–**14**, **16**–**21**) are an example of compounds that can be found occurring across different species of the same genus (*Cystophora*), while the xanthophylls (**15**) provide an example of compounds that can extend over different genera (*Halopteris* and *Sargassum*). The application of HPLC-NMR to investigate either genera or species of marine algae which were previously not studied (*S. decipiens*, *S. vestitum*, *S. cf. fallax*, and *H. pseudospicata*) and to rapidly identify structure classes present, without the need for traditional and lengthy isolation procedures is noteworthy. Finally, this study also highlights the advantage of using HPLC-NMR over HPLC-MS data for dereplication, especially when compounds do not easily ionize during HPLC-MS analyses. In such cases, crucial NMR data, which can be acquired via HPLC-NMR, provides important structural features and connectivity, which can enable a compound to be dereplicated. Unlike HPLC-MS, HPLC-NMR also allows for the differentiation of structural isomers.

## Supplementary Information

HPLC-NMR and HPLC-MS data and UV profiles for the crude extracts of the seven marine algae are included (these are listed in order of retention time). This material is available free of charge via MDPI Website.
