# Supplementary material for: Dereplication and Chemotaxonomical Studies of Marine Algae of the Ochrophyta and Rhodophyta Phyla
Source: Mar Drugs. 2015 Apr 30;13(5):2714–31. doi: 10.3390/md13052714 (PMC4446602; doi:10.3390/md13052714)
Supplement: Supplementary file 1 [file marinedrugs-13-02714-s001.pdf]

Supplementary Information

Order Based on Retention Time

Figure S1. Extracted UV profile of compound eluting at 2.29 min (**2**) from HPLC-NMR (*S. decipiens*).
Figure S2. WET1D Proton NMR spectrum (500 MHz, 75% CH_3CN/D_2O) of compound eluting at 2.29 min (**2**) (*S. decipiens*).
Figure S3. gCOSY NMR spectrum (500 MHz, 75% CH_3CN/D_2O) of compounds eluting at 2.29 (**2**) and 2.44 min (**3**) (peaks diffused during stop-flow analysis) (*S. decipiens*).
Figure S4. HSQCAD NMR spectrum (500 MHz, 75% CH_3CN/D_2O) of compounds eluting at 2.29 (**2**) and 2.44 min (**3**) (peaks diffused during stop-flow analysis) (*S. decipiens*).
Figure S5. gHMBCAD NMR spectrum (500 MHz, 75% CH_3CN/D_2O) of compounds eluting at 2.29 (**2**) and 2.44 min (**3**) (peaks diffused during stop-flow analysis) (*S. decipiens*).
Figure S6. High resolution negative ESI-MS of compound eluting at 2.29 min (**2**) from HPLC-MS (*S. decipiens*).
Figure S7. NMR data for compound eluting at 2.29 min (**2**) (*S. decipiens*).
Figure S8. Extracted UV profile of compound eluting at 2.44 min (**3**) from HPLC-NMR (*S. decipiens*).
Figure S9. WET1D Proton NMR spectrum (500 MHz, 75% CH_3CN/D_2O) of compound eluting at 2.44 min (**3**) (*S. decipiens*).
Figure S10. High resolution negative ESI-MS of compound eluting at 2.44 min (**3**) from HPLC-MS (*S. decipiens*).
Figure S11. NMR data for compound eluting at 2.44 min (**3**) (*S. decipiens*).
Figure S12. Extracted UV profile of compound eluting at 3.42 min (**11**) from HPLC-NMR (*C. retroflexa*).
Figure S13. WET1D Proton NMR spectrum (500 MHz, 75% CH_3CN/D_2O) of compound eluting at 3.42 min (**11**) (*C. retroflexa*).
Figure S14. High resolution negative ESI-MS of compound eluting at 3.42 min (**11**) from HPLC-MS (*C. retroflexa*).
Figure S15. NMR data for compound eluting at 3.42 min (**11**) (*C. retroflexa*).
Figure S16. Extracted UV profile of compound eluting at 3.55 min (**1**) from HPLC-NMR (*S. decipiens*).
Figure S17. WET1D Proton NMR spectrum (500 MHz, 75% CH_3CN/D_2O) of compound eluting at 3.55 min (**1**) (*S. decipiens*).
Figure S18. gCOSY NMR spectrum (500 MHz, 75% CH_3CN/D_2O) of compound eluting at 3.55 min (**1**) (*S. decipiens*).
Figure S19. High resolution negative ESI-MS of compound eluting at 3.55 min (**1**) from HPLC-MS (*S. decipiens*).
Figure S20. NMR data for compound eluting at 3.55 min (**1**) (*S. decipiens*).
Figure S21. Extracted UV profile of compound eluting at 4.45 min (**16**) from HPLC-NMR (*C. retroflexa*).
Figure S22. WET1D Proton NMR spectrum (500 MHz, 75% CH_3CN/D_2O) of compound eluting at 4.45 min (**16**) (*C. retroflexa*).

Figure S23. gCOSY NMR spectrum (500 MHz, 75% CH_3CN/D_2O) of compound eluting at 4.45 min (**16**) (*C. retroflexa*).
Figure S24. HSQCAD NMR spectrum (500 MHz, 75% CH_3CN/D_2O) of compound eluting at 4.45 min (**16**) (*C. retroflexa*).
Figure S25. gHMBCAD NMR spectrum (500 MHz, 75% CH_3CN/D_2O) of compound eluting at 4.45 min (**16**) (*C. retroflexa*).
Figure S26. High resolution negative ESI-MS of compound eluting at 4.45 min (**16**) from HPLC-MS (*C. retroflexa*).
Figure S27. Extracted UV profile of compound eluting at 5.00 min from HPLC-NMR (*Laurencia* sp.).
Figure S28. WET1D Proton NMR spectrum (500 MHz, 75% CH_3CN/D_2O) of compound eluting at 5.00 min (*Laurencia* sp.).
Figure S29. gCOSY NMR spectrum (500 MHz, 75% CH_3CN/D_2O) of compound eluting at 5.00 min (*Laurencia* sp.).
Figure S30. Extracted UV profile of compound eluting at 6.05 min from HPLC-NMR (*Laurencia* sp.).
Figure S31. WET1D Proton NMR spectrum (500 MHz, 75% CH_3CN/D_2O) of compound eluting at 6.05 min (*Laurencia* sp.).
Figure S32. gCOSY NMR spectrum (500 MHz, 75% CH_3CN/D_2O) of compound eluting at 6.05 min (*Laurencia* sp.).
Figure S33. HSQCAD NMR spectrum (500 MHz, 75% CH_3CN/D_2O) of compound eluting at 6.05 min (*Laurencia* sp.).
Figure S34. gHMBCAD NMR spectrum (500 MHz, 75% CH_3CN/D_2O) of compound eluting at 6.05 min (*Laurencia* sp.).
Figure S35. Extracted UV profile of compound eluting at 6.70 min from HPLC-NMR (*Laurencia* sp.).
Figure S36. WET1D Proton NMR spectrum (500 MHz, 75% CH_3CN/D_2O) of compound eluting at 6.70 min (*Laurencia* sp.).
Figure S37. gCOSY NMR spectrum (500 MHz, 75% CH_3CN/D_2O) of compound eluting at 6.70 min (*Laurencia* sp.).
Figure S38. Extracted UV profile of compound eluting at 7.87 min (**4**) from HPLC-NMR (*S. decipiens*).
Figure S39. WET1D Proton NMR spectrum (500 MHz, 75% CH_3CN/D_2O) of compound eluting at 7.87 min (**4**) (*S. decipiens*).
Figure S40. gCOSY NMR spectrum (500 MHz, 75% CH_3CN/D_2O) of compound eluting at 7.87 min (**4**) (*S. decipiens*).
Figure S41. High resolution negative ESI-MS of compound eluting at 7.87 min (**4**) from HPLC-MS (*S. decipiens*).
Figure S42. NMR data for compound eluting at 7.87 min (**4**) (*S. decipiens*).
Figure S43. Extracted UV profile of compound eluting at 9.98 min (**12**) from HPLC-NMR (*C. retroflexa*).
Figure S44. WET1D Proton NMR spectrum (500 MHz, 75% CH_3CN/D_2O) of compound eluting at 9.98 min (**12**) (*C. retroflexa*).
Figure S45. gCOSY NMR spectrum (500 MHz, 75% CH_3CN/D_2O) of compound eluting at 9.98 min (**12**) (*C. retroflexa*).

Figure S46. HSQCAD NMR spectrum (500 MHz, 75% CH_3CN/D_2O) of compound eluting at 9.98 min (**12**) (*C. retroflexa*).
Figure S47. gHMBCAD NMR spectrum (500 MHz, 75% CH_3CN/D_2O) of compound eluting at 9.98 min (**12**) (*C. retroflexa*).
Figure S48. High resolution negative ESI-MS of compound eluting at 9.98 min (**12**) from HPLC-MS (*C. retroflexa*).
Figure S49. NMR data for compound eluting at 9.98 min (**12**) (*C. retroflexa*).
Figure S50. Extracted UV profile of compound eluting at 12.95 min (**13**) from HPLC-NMR (*C. retroflexa*).
Figure S51. WET1D Proton NMR spectrum (500 MHz, 75% CH_3CN/D_2O) of compound eluting at 12.95 min (**13**) (*C. retroflexa*).
Figure S52. gCOSY NMR spectrum (500 MHz, 75% CH_3CN/D_2O) of compound eluting at 12.95 min (**13**) (*C. retroflexa*).
Figure S53. HSQCAD NMR spectrum (500 MHz, 75% CH_3CN/D_2O) of compound eluting at 12.95 min (**13**) (*C. retroflexa*).
Figure S54. High resolution negative ESI-MS of compound eluting at 12.95 min (**13**) from HPLC-MS (*C. retroflexa*).
Figure S55. NMR data for compound eluting at 12.95 min (**13**) (*C. retroflexa*).
Figure S56. Extracted UV profile of compound eluting at 13.65 min (**17**) from HPLC-NMR (*S. cf. fallax*).
Figure S57. WET1D Proton NMR spectrum (500 MHz, 75% CH_3CN/D_2O) of compound eluting at 13.65 min (**17**) (*S. cf. fallax*).
Figure S58. gCOSY NMR spectrum (500 MHz, 75% CH_3CN/D_2O) of compound eluting at 13.65 min (**17**) (*S. cf. fallax*).
Figure S59. High resolution negative ESI-MS of compound eluting at 13.65 min (**17**) from HPLC-MS (*S. cf. fallax*).
Figure S60. Extracted UV profile of compound eluting at 14.53 min (**5**) from HPLC-NMR (*H. pseudospicata*).
Figure S61. WET1D Proton NMR spectrum (500 MHz, 75% CH_3CN/D_2O) of compound eluting at 14.53 min (**5**) (*H. pseudospicata*).
Figure S62. gCOSY NMR spectrum (500 MHz, 75% CH_3CN/D_2O) of compound eluting at 14.53 min (**5**) (*H. pseudospicata*).
Figure S63. HSQCAD NMR spectrum (500 MHz, 75% CH_3CN/D_2O) of compound eluting at 14.53 min (**5**) (*H. pseudospicata*).
Figure S64. ROESYAD NMR spectrum (500 MHz, 75% CH_3CN/D_2O) of compound eluting at 14.53 min (**5**) (*H. pseudospicata*).
Figure S65. High resolution negative ESI-MS of compound eluting at 14.53 min (**5**) from HPLC-MS (*H. pseudospicata*).
Figure S66. High resolution positive ESI-MS of compound eluting at 14.53 min (**5**) from HPLC-MS (*H. pseudospicata*).
Figure S67. NMR data for compound eluting at 14.53 min (**5**) (*H. pseudospicata*).

Figure S68. Extracted UV profile of compound eluting at 15.50 min (**20**) from HPLC-NMR (*S. cf. fallax*).
Figure S69. WET1D Proton NMR spectrum (500 MHz, 75% CH_3CN/D_2O) of compound eluting at 15.50 min (**20**) (*S. cf. fallax*).
Figure S70. High resolution negative ESI-MS of compound eluting at 15.50 min (**20**) from HPLC-MS (*S. cf. fallax*).
Figure S71. NMR data for compound eluting at 15.50 min (**20**) (*S. cf. fallax*).
Figure S72. Extracted UV profile of compound eluting at 20.15 min (**21**) from HPLC-NMR (*C. retroflexa*).
Figure S73. WET1D Proton NMR spectrum (500 MHz, 75% CH_3CN/D_2O) of compound eluting at 20.15 min (**21** (*C. retroflexa*)).
Figure S74. High resolution negative ESI-MS of compound eluting at 20.15 min (**21**) from HPLC-MS (*C. retroflexa*).
Figure S75. NMR data for compound eluting at 20.15 min (**21**) (*C. retroflexa*).
Figure S76. Extracted UV profile of compound eluting at 21.62 min (**14**) from HPLC-NMR (*S. cf. fallax*).
Figure S77. WET1D Proton NMR spectrum (500 MHz, 75% CH_3CN/D_2O) of compound eluting at 21.62 min (**14**) (*S. cf. fallax*).
Figure S78. High resolution negative ESI-MS of compound eluting at 21.62 min (**14**) from HPLC-MS (*S. cf. fallax*).
Figure S79. NMR data for compound eluting at 21.62 min (**14**) (*S. cf. fallax*).
Figure S80. Extracted UV profile of compound eluting at 22.96 min (**18**) from HPLC-NMR (*C. subfarcinata*).
Figure S81. WET1D Proton NMR spectrum (500 MHz, 75% CH_3CN/D_2O) of compound eluting at 22.96 min (**18**) (*C. subfarcinata*).
Figure S82. High resolution negative ESI-MS of compound eluting at 22.96 min (**18**) from HPLC-MS (*C. subfarcinata*).
Figure S83. Extracted UV profile of compound eluting at 23.16 min from HPLC-NMR (*C. retroflexa*).
Figure S84. WET1D Proton NMR spectrum (500 MHz, 75% CH_3CN/D_2O) of compound eluting at 23.16 min (*C. retroflexa*).
Figure S85. Extracted UV profile of compound eluting at 26.71 min from HPLC-NMR (*H. pseudospicata*).
Figure S86. Extracted UV profile of compound eluting at 30.27 min from HPLC-NMR (*H. pseudospicata*).
Figure S87. Extracted UV profile of compound eluting at 33.40 min (**19**) from HPLC-NMR (*C. subfarcinata*).
Figure S88. WET1D Proton NMR spectrum (500 MHz, 75% CH_3CN/D_2O) of compound eluting at 33.40 min (**19**) (*C. subfarcinata*).
Figure S89. High resolution negative ESI-MS of compound eluting at 33.40 min (**19**) from HPLC-MS (*C. subfarcinata*).

Figure S90. Extracted UV profile of compound eluting at 60.80 min (**15**) from HPLC-NMR (*S. cf. fallax*).

Figure S91. WET1D Proton NMR spectrum (500 MHz, 75% CH_3CN/D_2O) of compound eluting at 60.80 min (**15**) (*S. cf. fallax*).

Figure S92. NMR data for compound eluting at 60.80 min (**15**) (*S. cf. fallax*).

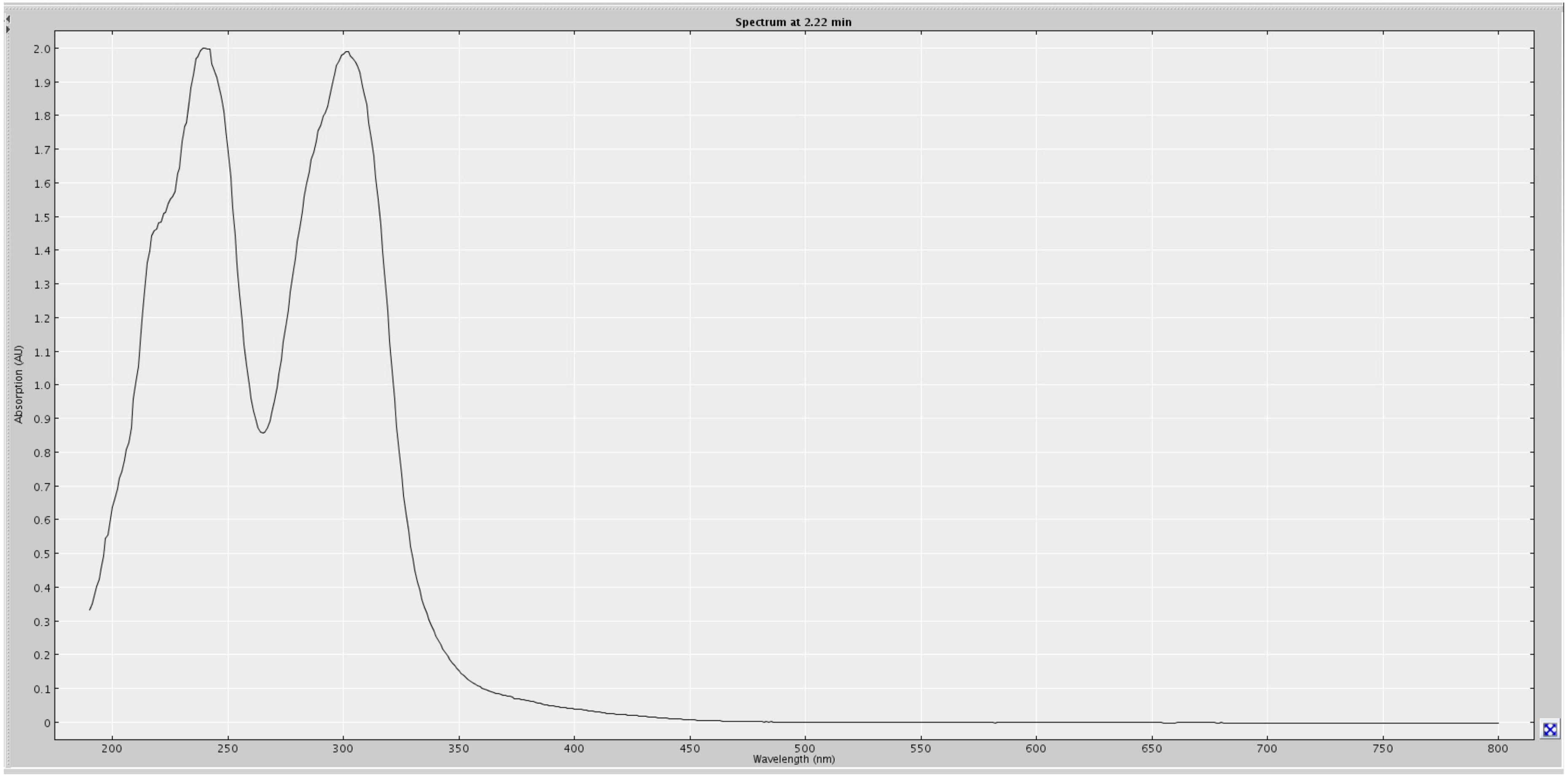

Figure S1. Extracted UV profile of compound eluting at 2.29 min (**2**) from HPLC-NMR (*S. decipiens*).

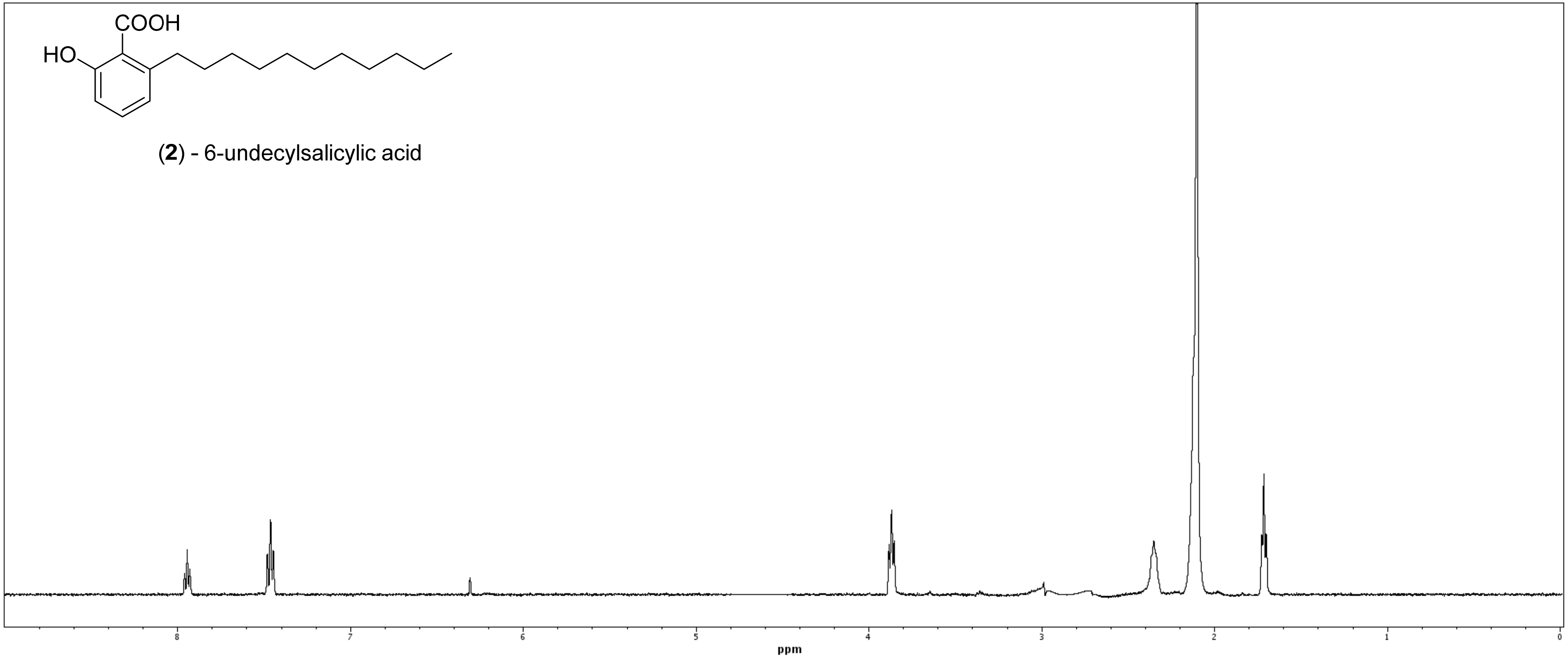

Figure S2. WET1D Proton NMR spectrum (500 MHz, 75% CH_3CN/D_2O) of compound eluting at 2.29 min (**2**) (*S. decipiens*).

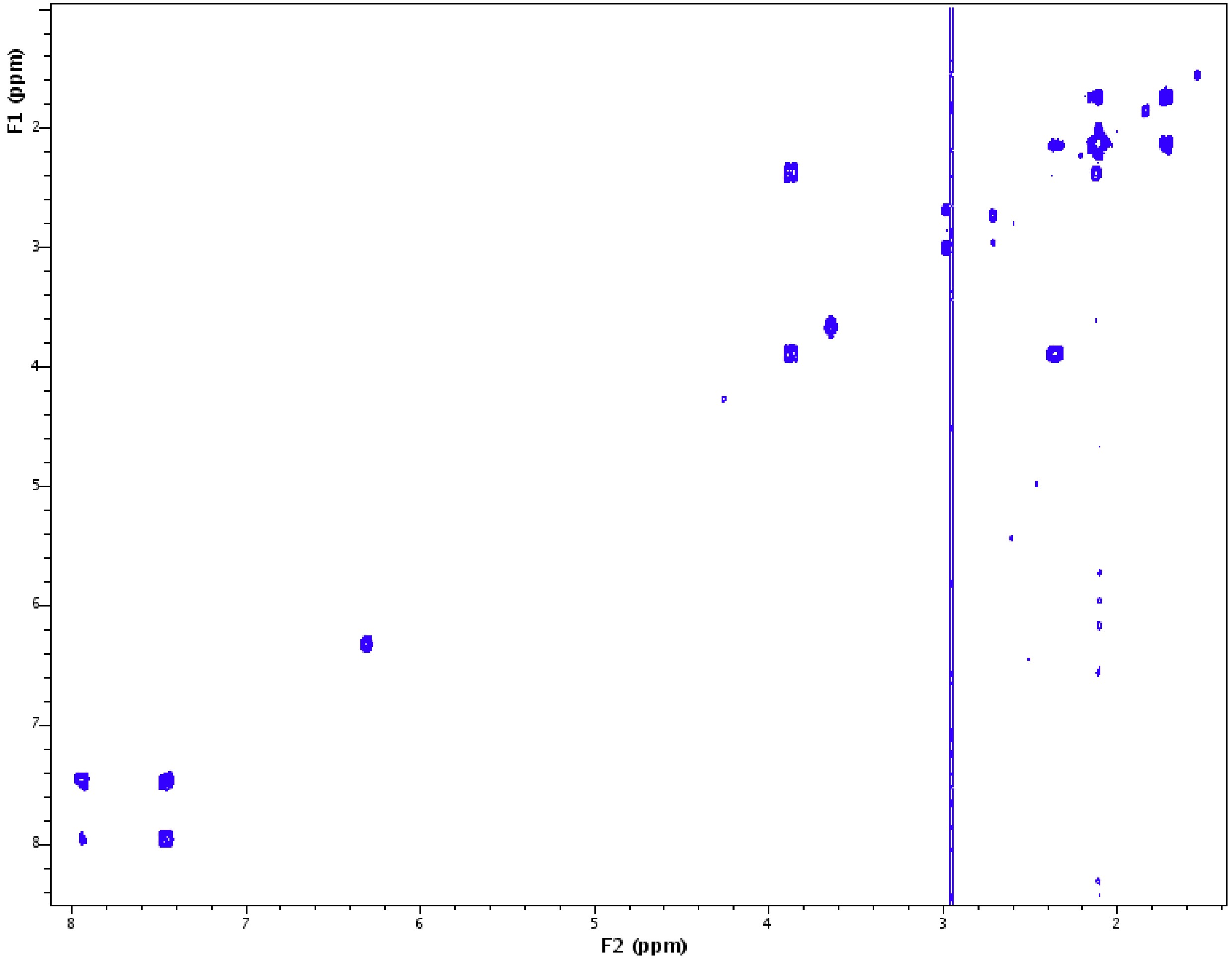

Figure S3. gCOSY NMR spectrum (500 MHz, 75% CH_3CN/D_2O) of compounds eluting at 2.29 (**2**) and 2.44 min (**3**) (peaks diffused during stop-flow analysis) (*S. decipiens*).

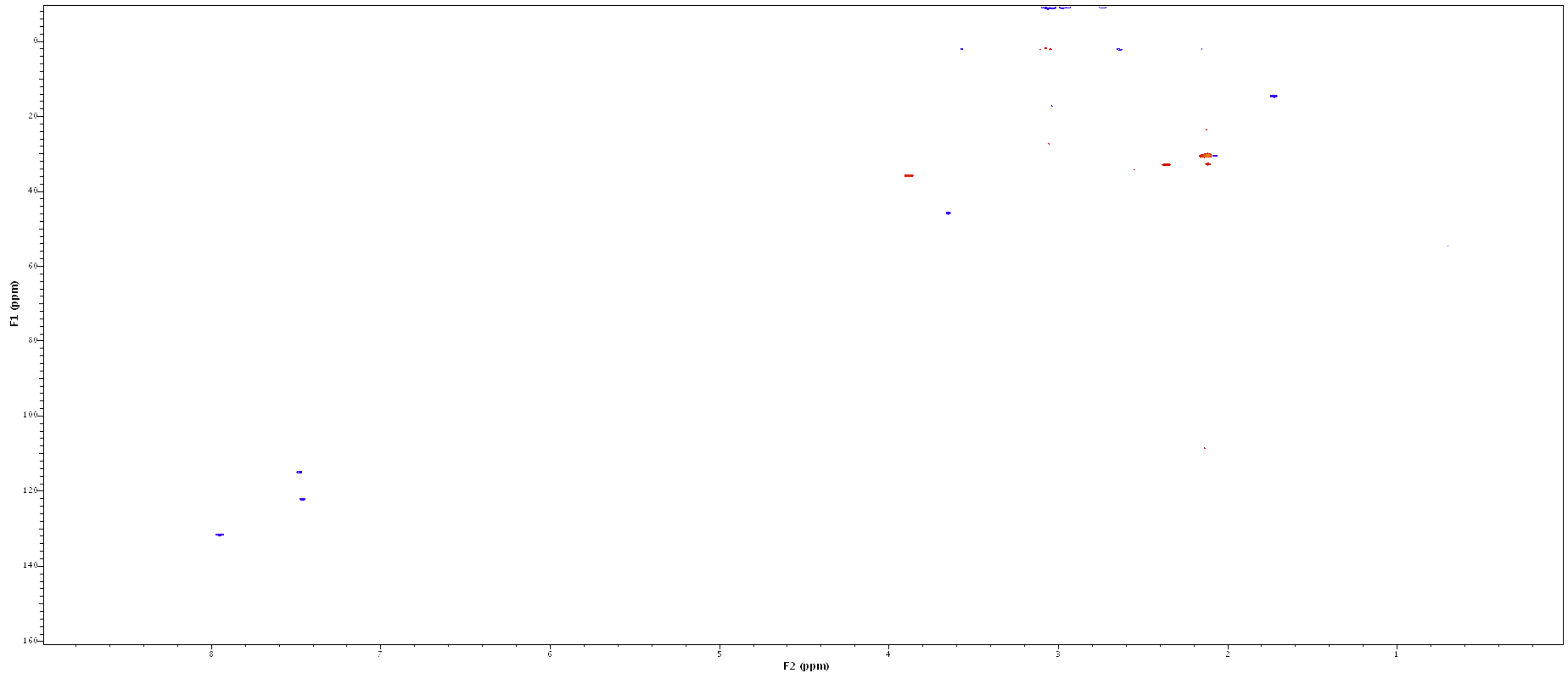

Figure S4. HSQCAD NMR spectrum (500 MHz, 75% CH_3CN/D_2O) of compounds eluting at 2.29 (**2**) and 2.44 min (**3**) (peaks diffused during stop-flow analysis) (*S. decipiens*).

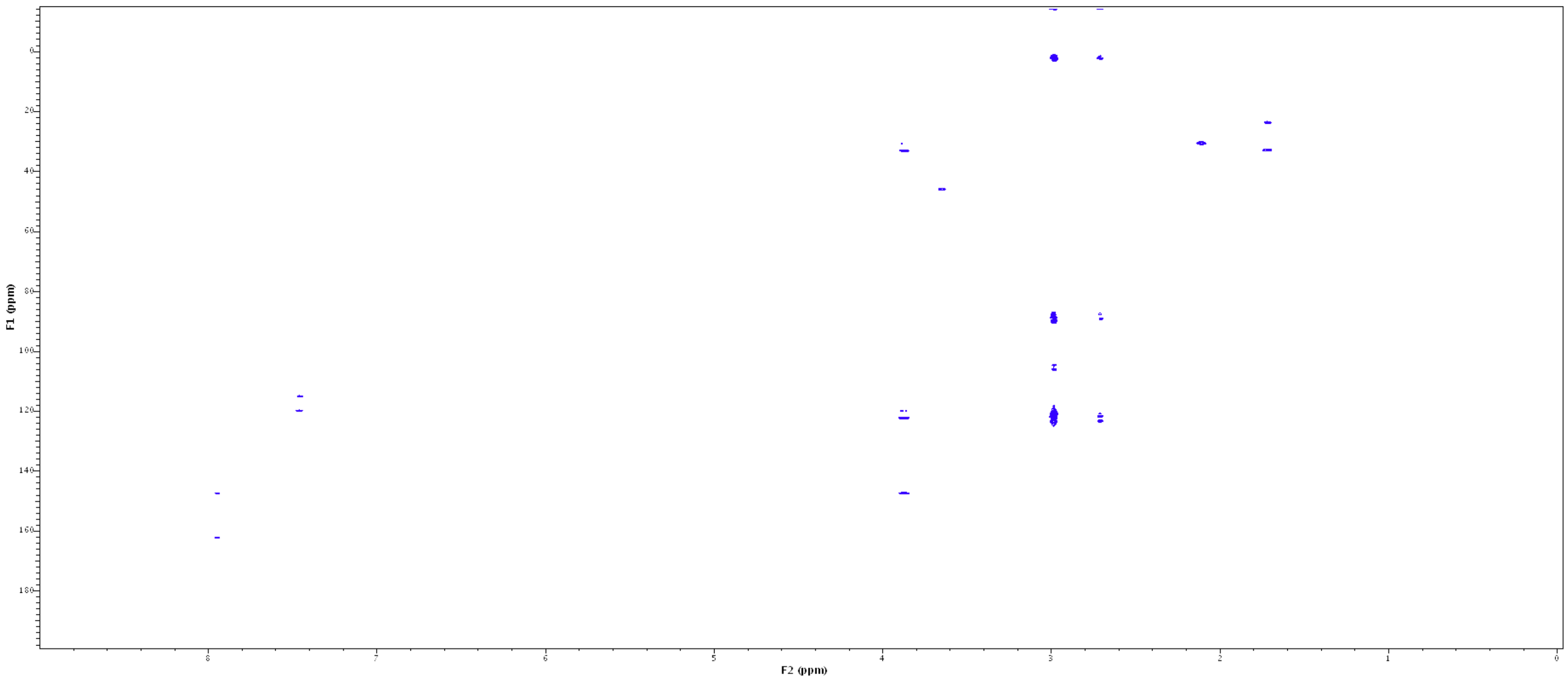

Figure S5. gHMBCAD NMR spectrum (500 MHz, 75% CH_3CN/D_2O) of compounds eluting at 2.29 (**2**) and 2.44 min (**3**) (peaks diffused during stop-flow analysis) (*S. decipiens*).

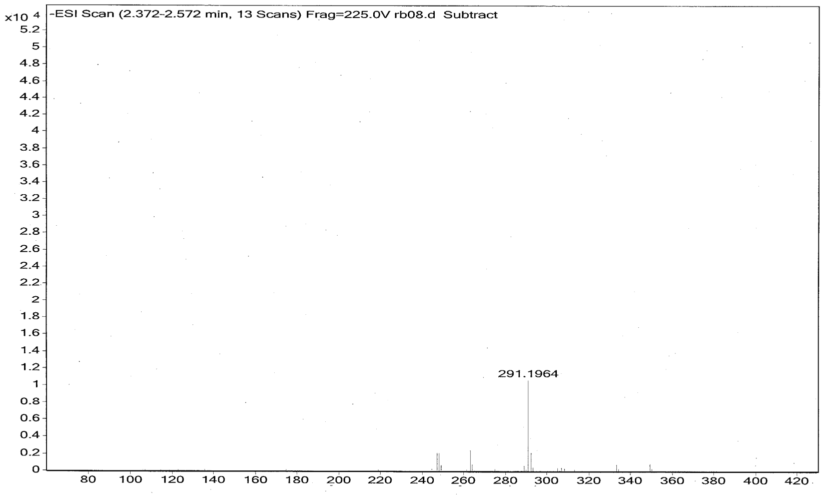

Figure S6. High resolution negative ESI-MS of compound eluting at 2.29 min (**2**) from HPLC-MS (*S. decipiens*).

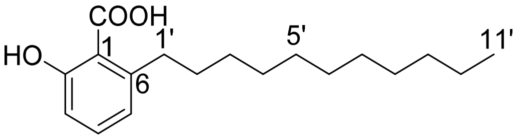

(**2**) - 6-undecylsalicylic acid

Position	δC [a], mult.	δH (*J* in Hz)	gCOSY	gHMBCAD
1	119.6, s			
2	162.0, s			
3	114.7, d	7.47, d (8.0)	4	-
4	131.4, d	7.94, dd (8.0, 8.0)	3, 5	2, 6
5	121.9, d	7.45, d (8.0)	4	1, 3
6	147.2, s			
7	ND			
1′	35.5, t	3.87, t (7.5)	2′	1, 5, 6, 2′, 3′
2′	32.7, t	2.35, m	1′	3′, 4′
3′	30.2, t	2.10, m		4′, 5′
4′	30.2, t	2.10, m		3′, 5′, 6′
5′	30.2, t	2.10, m		3′, 4′, 6′, 7′
6′	30.2, t	2.10, m		4′, 5′, 7′, 8′
7′	30.2, t	2.10, m		5′, 6′, 8′
8′	30.2, t	2.10, m		6′, 7′
9′	32.5, t	2.10, m		7′, 8′
10′	23.2, t	2.10, m	11′	8′
11′	14.3, q	1.71, t (6.0)	10′	9′, 10′
2-OH		ND		
7-OH		ND		

Referenced to 75% CH_3CN/D_2O; [a] Carbon assignments based on HSQCAD and gHMBCAD NMR experiments; ND Not Detected.

Figure S7. NMR data for compound eluting at 2.29 min (**2**) (*S. decipiens*).

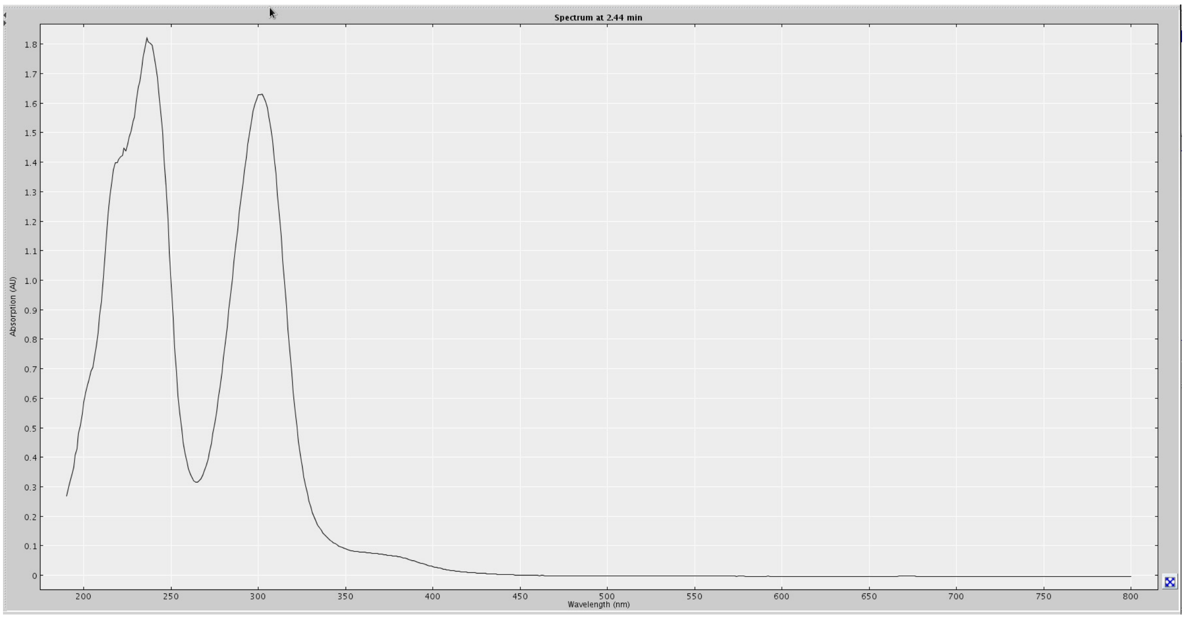

Figure S8. Extracted UV profile of compound eluting at 2.44 min (**3**) from HPLC-NMR (*S. decipiens*).

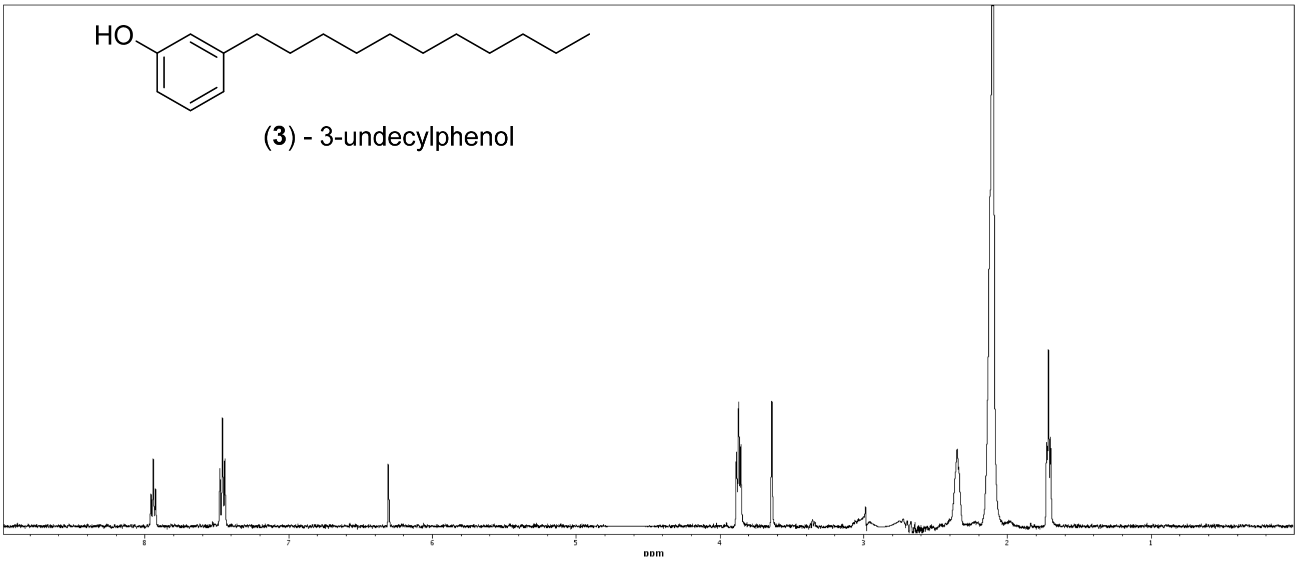

Figure S9. WET1D Proton NMR spectrum (500 MHz, 75% CH_3CN/D_2O) of compound eluting at 2.44 min (**3**) (*S. decipiens*).

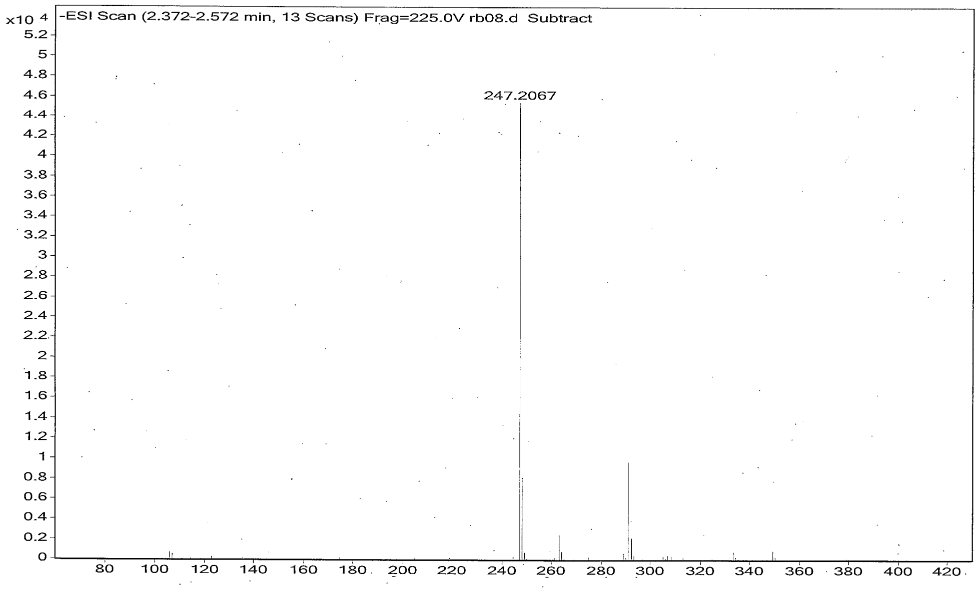

Figure S10. High resolution negative ESI-MS of compound eluting at 2.44 min (**3**) from HPLC-MS (*S. decipiens*).

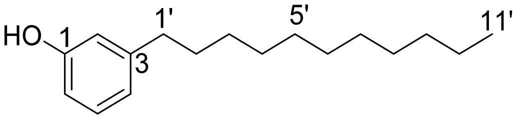

(**3**) - 3-undecylphenol

Position	δC [a], mult.	δH (*J* in Hz)	gCOSY	gHMBCAD
1	162.0, s			
2	ND	6.31, s		
3	147.2, s			
4	121.9, d	7.45, d (8.5)	5	6
5	131.5, d	7.94, dd (8.5, 9.0)	4, 6	1, 3
6	114.7, d	7.47, d (9.0)*	5	
1′	35.5, t	3.87, t (7.5)	2′	3, 4, 2′, 3′
2′	32.7, t	2.35, m	1′	3′, 4′
3′	30.2, t	2.10, m		4′, 5′
4′	30.2, t	2.10, m		3′, 5′, 6′
5′	30.2, t	2.10, m		3′, 4′, 6′, 7′
6′	30.2, t	2.10, m		4′, 5′, 7′, 8′
7′	30.2, t	2.10, m		5′, 6′, 8′
8′	30.2, t	2.10, m		6′, 7′
9′	32.5, t	2.10, m		7′, 8′
10′	23.2, t	2.10, m	11′	8′
11′	14.3, q	1.71, t (6.0)	10′	9′, 10′
1-OH		ND		

Referenced to 75% CH_3CN/D_2O; [a] Carbon assignments based on HSQCAD and gHMBCAD NMR experiments; ND Not Detected; * Signals overlapped.

Figure S11. NMR data for compound eluting at 2.44 min (**3**) (*S. decipiens*).

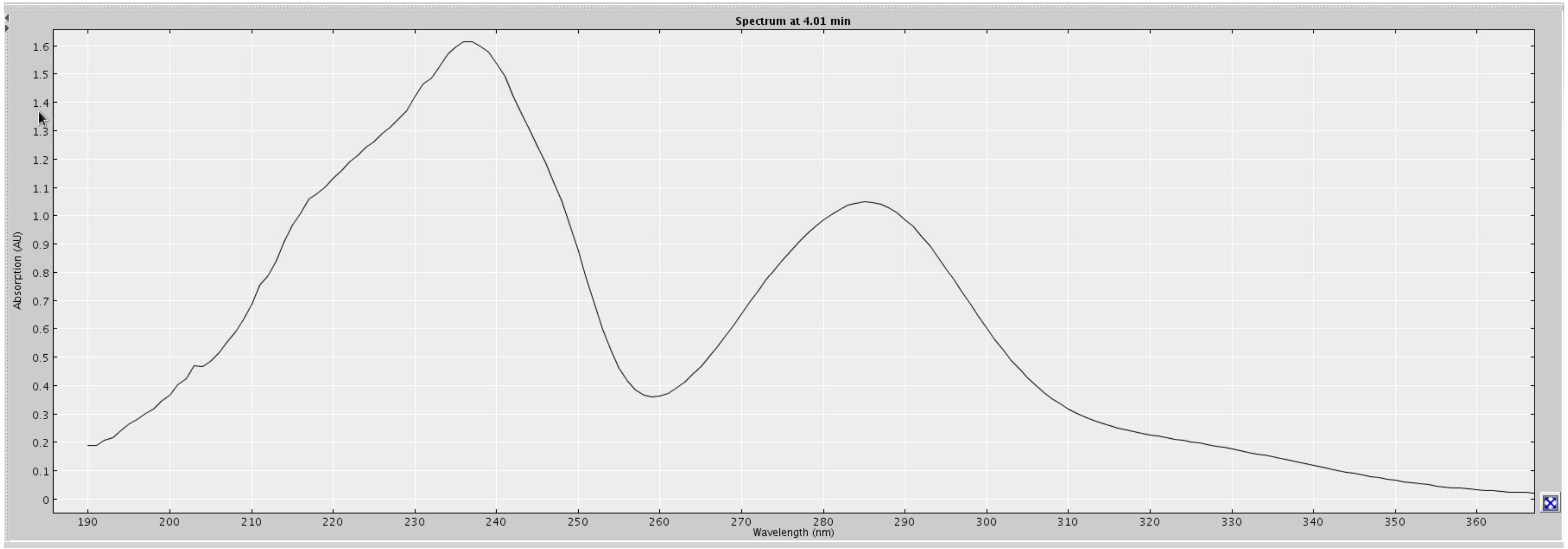

Figure S12. Extracted UV profile of compound eluting at 3.42 min (**11**) from HPLC-NMR (*C. retroflexa*).

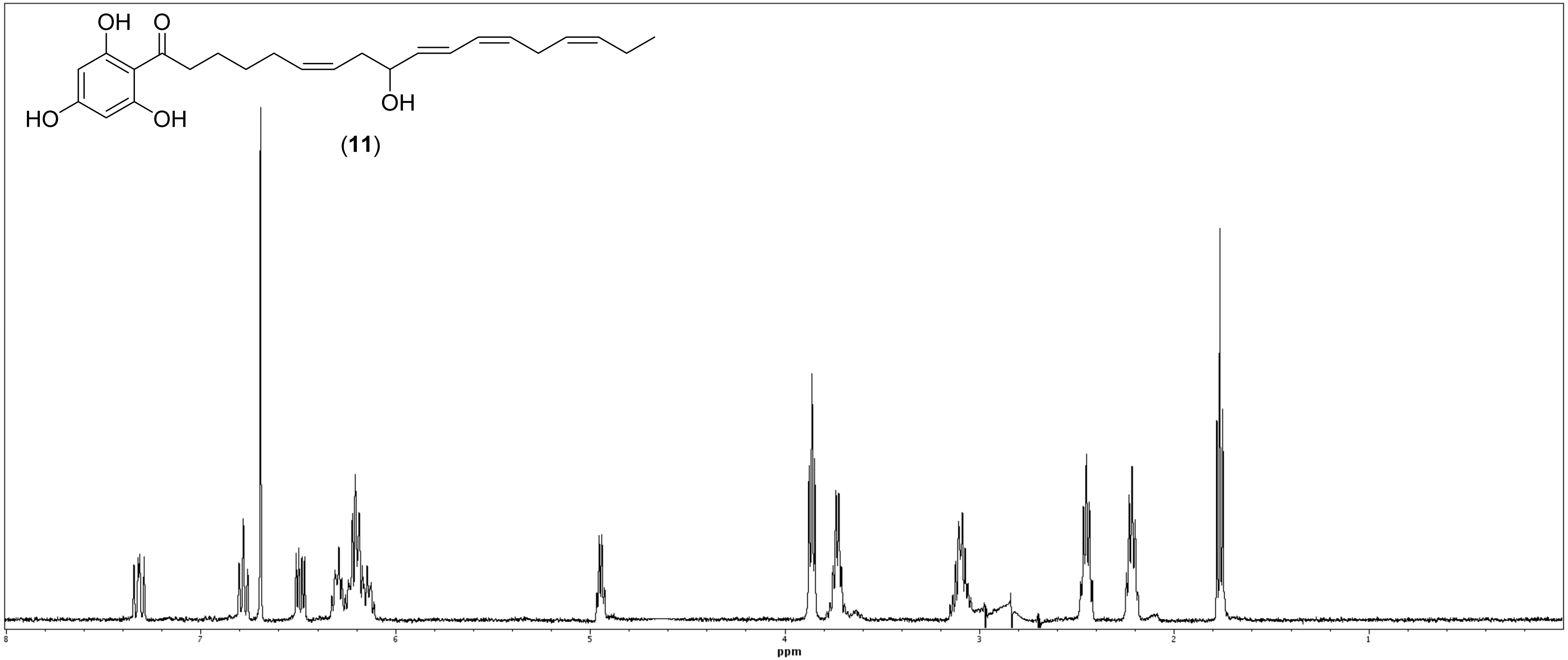

Figure S13. WET1D Proton NMR spectrum (500 MHz, 75% CH_3CN/D_2O) of compound eluting at 3.42 min (**11**) (*C. retroflexa*).

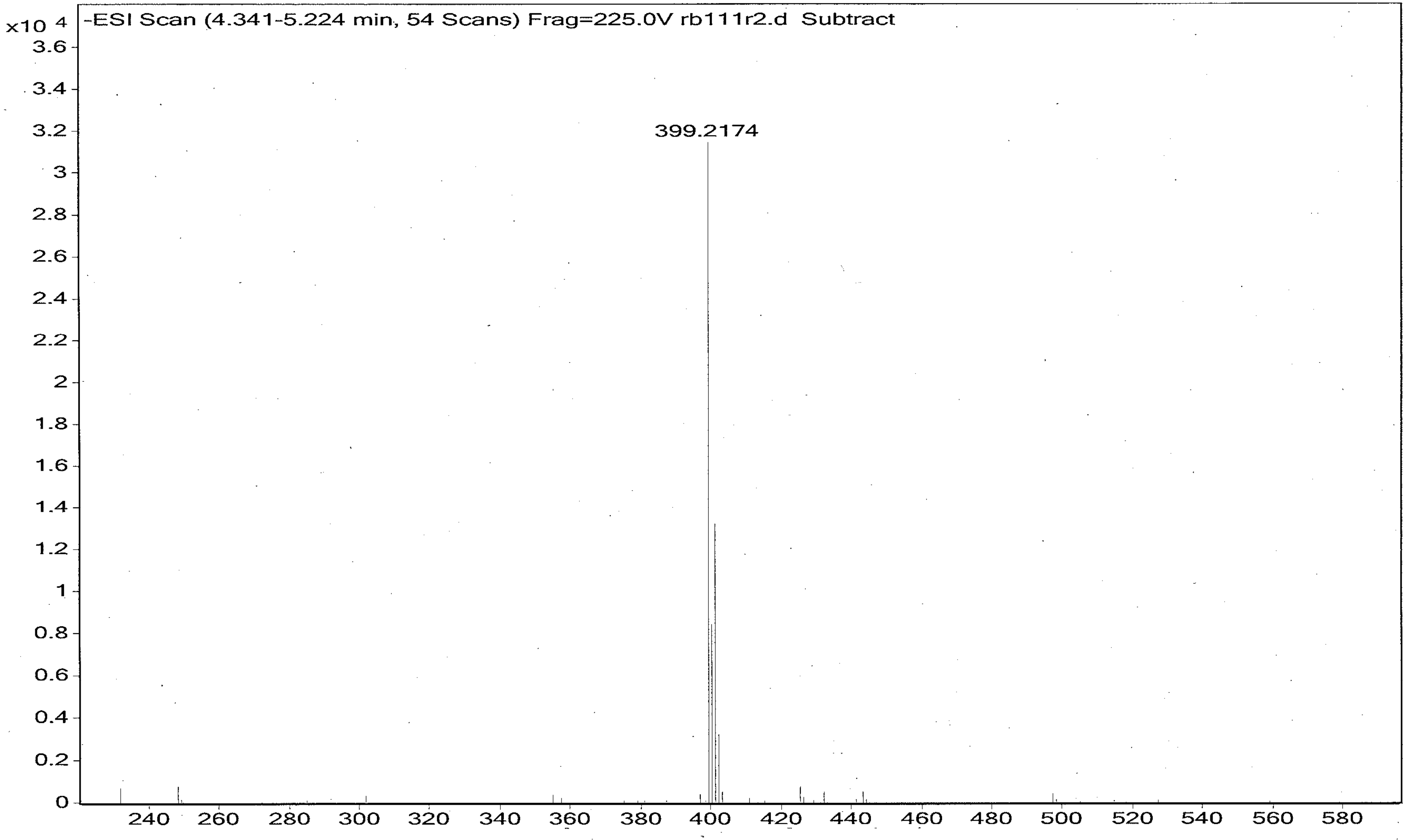

Figure S14. High resolution negative ESI-MS of compound eluting at 3.42 min (**11**) from HPLC-MS (*C. retroflexa*).

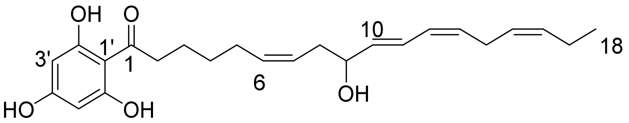

(**11**)

Position	δ_H (*J* in Hz)
1	
2	3.86, t (7.5)
3	2.45, p (7.5)
4	2.21, p (7.5)
5	3.10, dt (7.5, 9.5)
6	6.10–6.34, m
7	6.10–6.34, m
8	3.73, dd (7.0, 7.5)
9	4.95, dt (14.0, 7.0)
10	6.48, dd (15.0, 7.0)
11	7.32, dd (15.0, 11.0)
12	6.78, dd (11.0, 10.5)
13	6.10–6.34, m
14	SS
15	6.10–6.34, m
16	6.10–6.34, m
17	SS
18	1.76, t (7.5)
1′	
2′	
3′	6.69, s
4′	
5′	6.69, s
6′	
9-OH	ND
2′-OH	ND
4′-OH	ND
6′-OH	ND

Referenced to D_2O (δ_H 4.64 ppm); SS Signal suppressed; ND Not Detected.

Figure S15. NMR data for compound eluting at 3.42 min (**11**) (*C. retroflexa*).

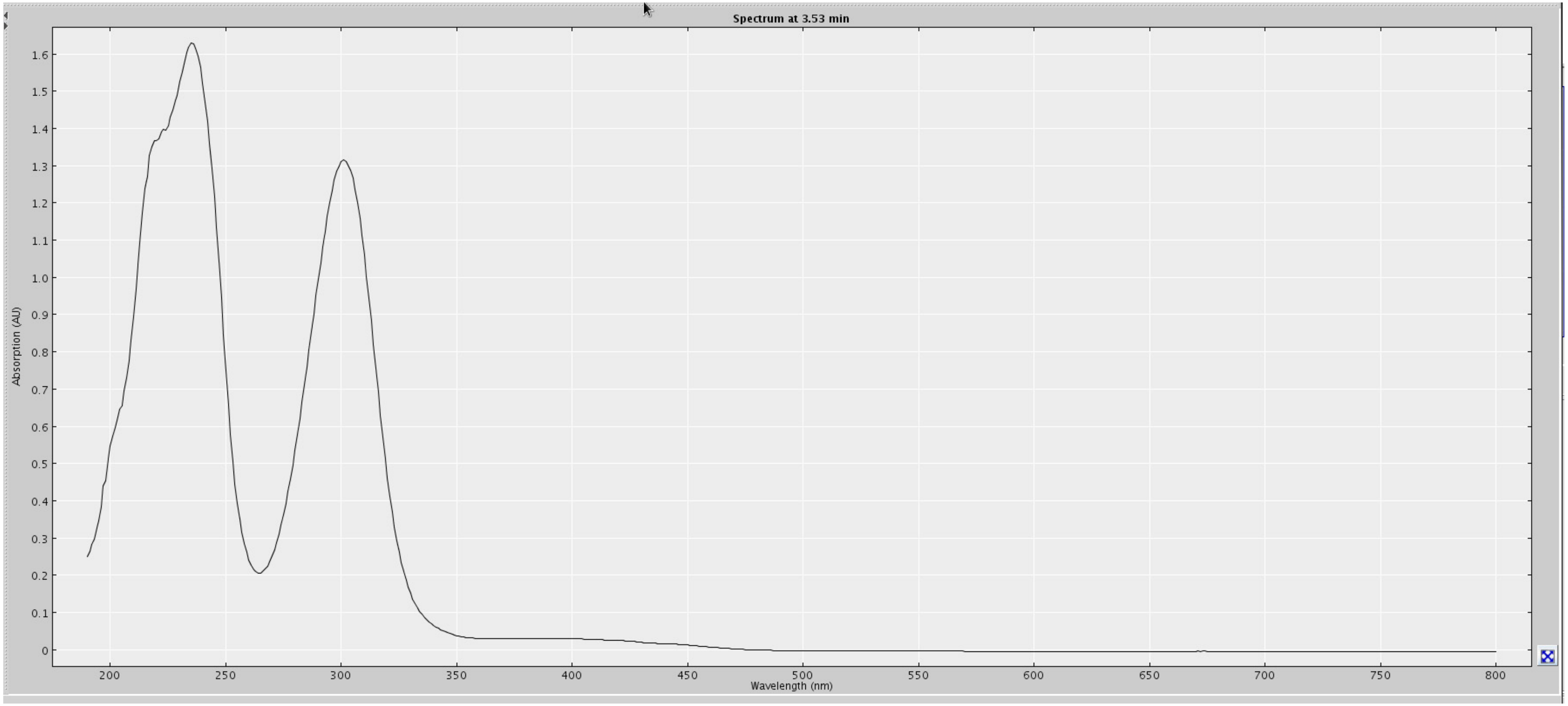

Figure S16. Extracted UV profile of compound eluting at 3.55 min (**1**) from HPLC-NMR (*S. decipiens*).

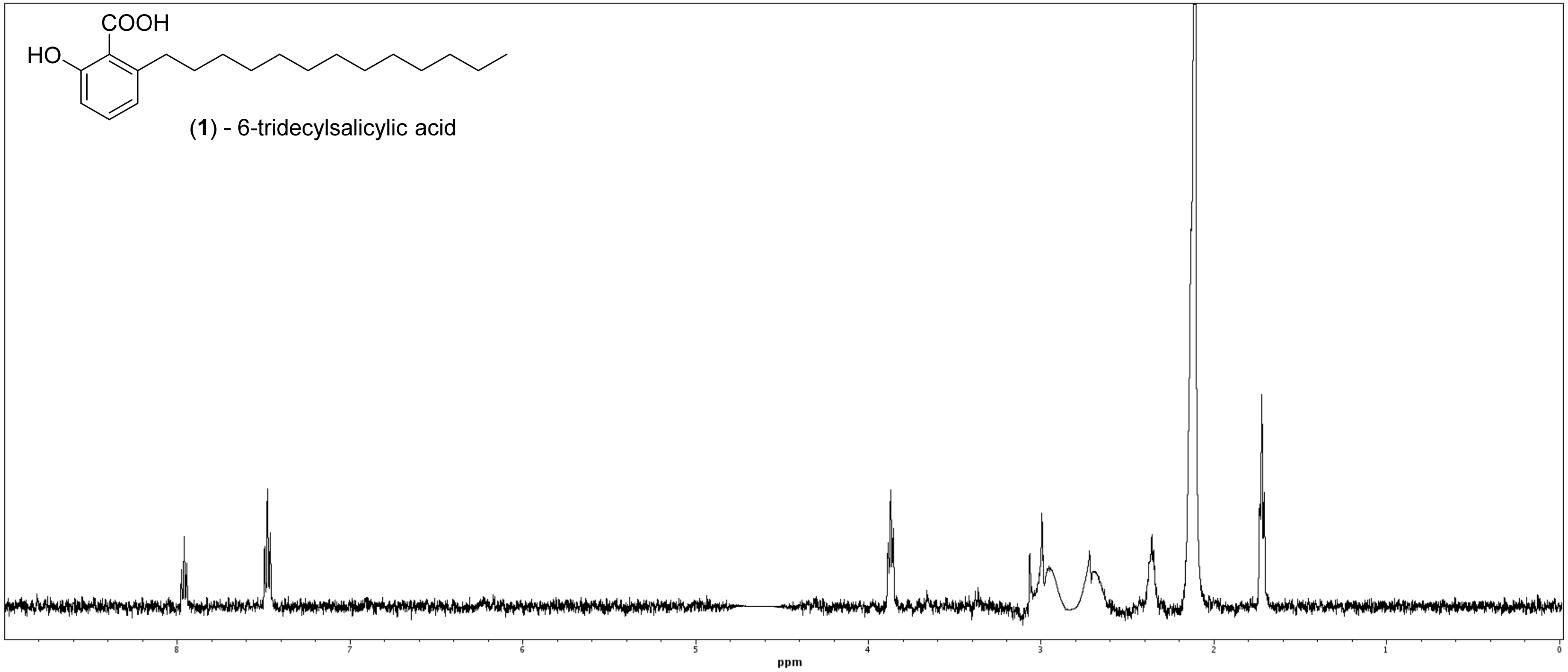

Figure S17. WET1D Proton NMR spectrum (500 MHz, 75% CH_3CN/D_2O) of compound eluting at 3.55 min (**1**) (*S. decipiens*).

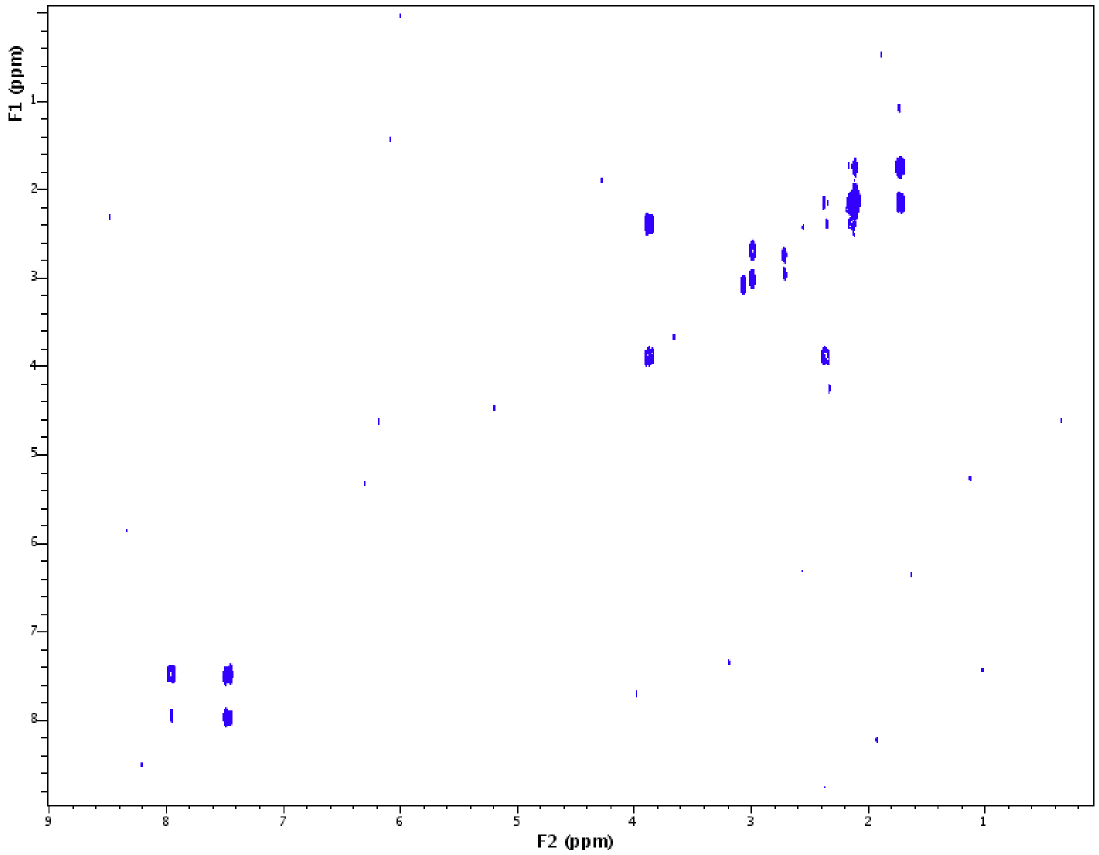

Figure S18. gCOSY NMR spectrum (500 MHz, 75% CH_3CN/D_2O) of compound eluting at 3.55 min (**1**) (*S. decipiens*).

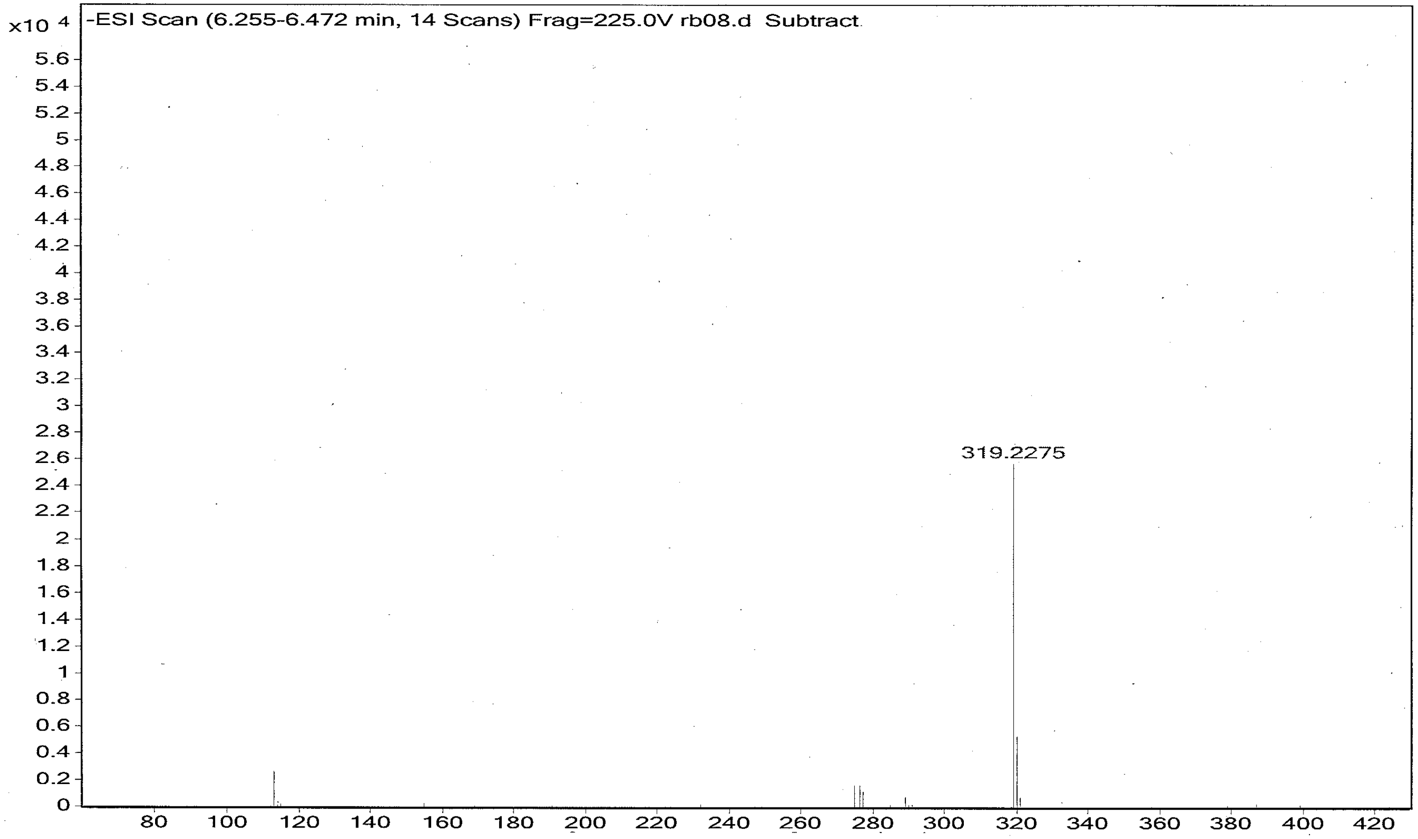

Figure S19. High resolution negative ESI-MS of compound eluting at 3.55 min (**1**) from HPLC-MS (*S. decipiens*).

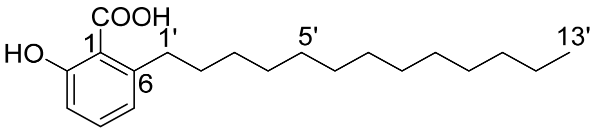

(**1**) - 6-tridecylsalicylic acid

Position	δ_H (*J* in Hz)	gCOSY
1		
2		
3	7.48, d (8.0)	4
4	7.96, dd (7.5, 8.0)	3, 5
5	7.47, d (7.5)	4
6		
7		
1′	3.87, t (8.5)	2′
2′	2.36, m	1′
3′	2.11, m	
4′	2.11, m	
5′	2.11, m	
6′	2.11, m	
7′	2.11, m	
8′	2.11, m	
9′	2.11, m	
10′	2.11, m	
11′	2.11, m	
12′	2.11, m	13′
13′	1.72, t (7.5)	12′
2-OH	ND	
7-OH	ND	

Referenced to 75% CH_3CN/D_2O; ND Not Detected.

Figure S20. NMR data for compound eluting at 3.55 min (**1**) (*S. decipiens*).

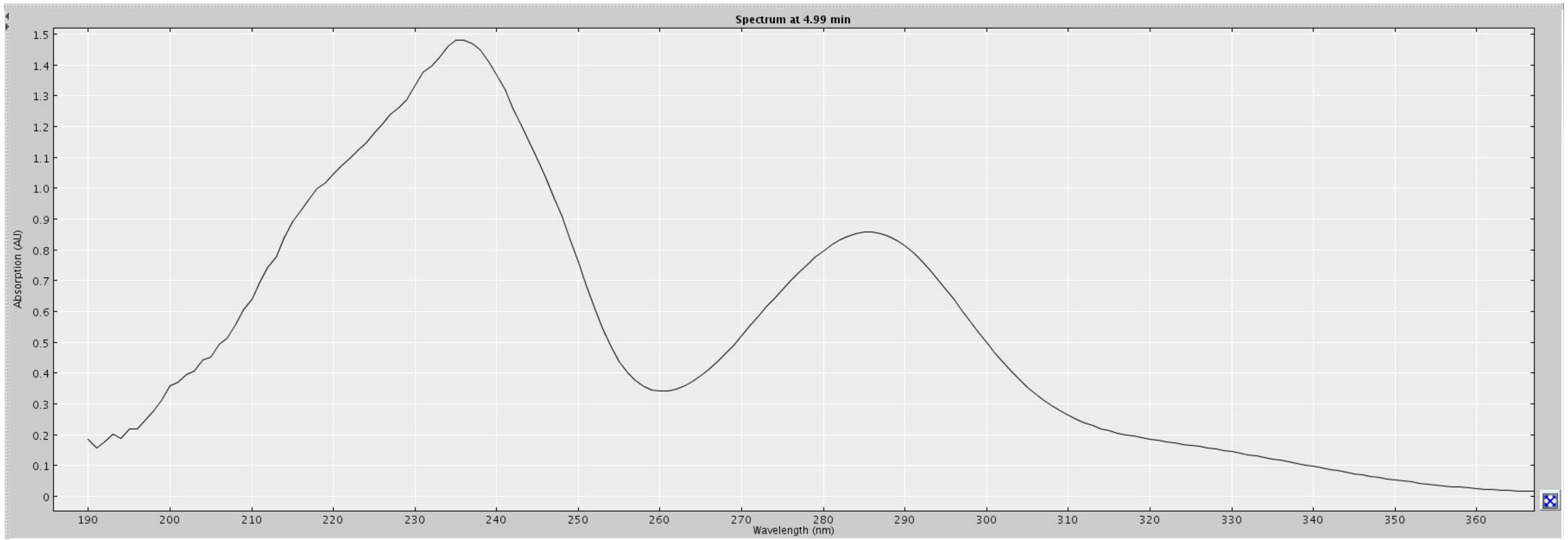

Figure S21. Extracted UV profile of compound eluting at 4.45 min (**16**) from HPLC-NMR (*C. retroflexa*).

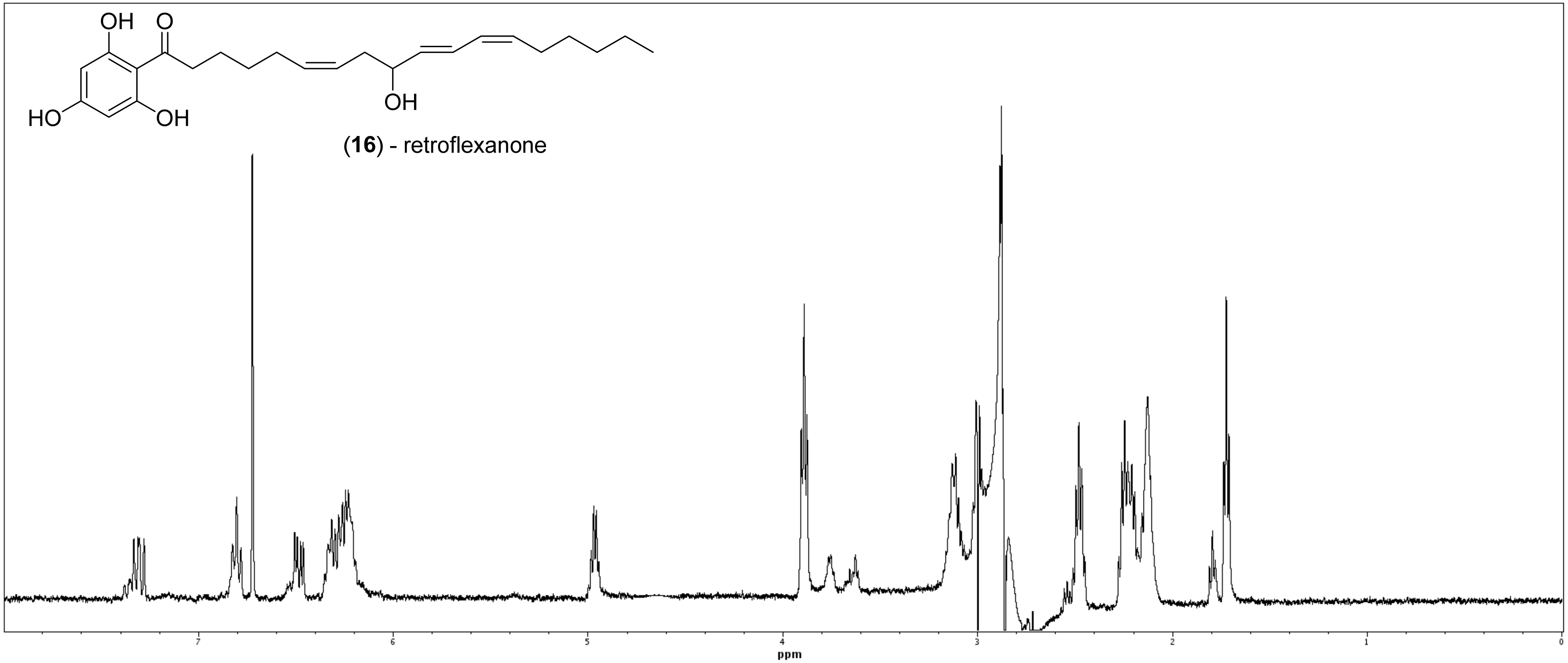

Figure S22. WET1D Proton NMR spectrum (500 MHz, 75% CH_3CN/D_2O) of compound eluting at 4.45 min (**16**) (*C. retroflexa*).

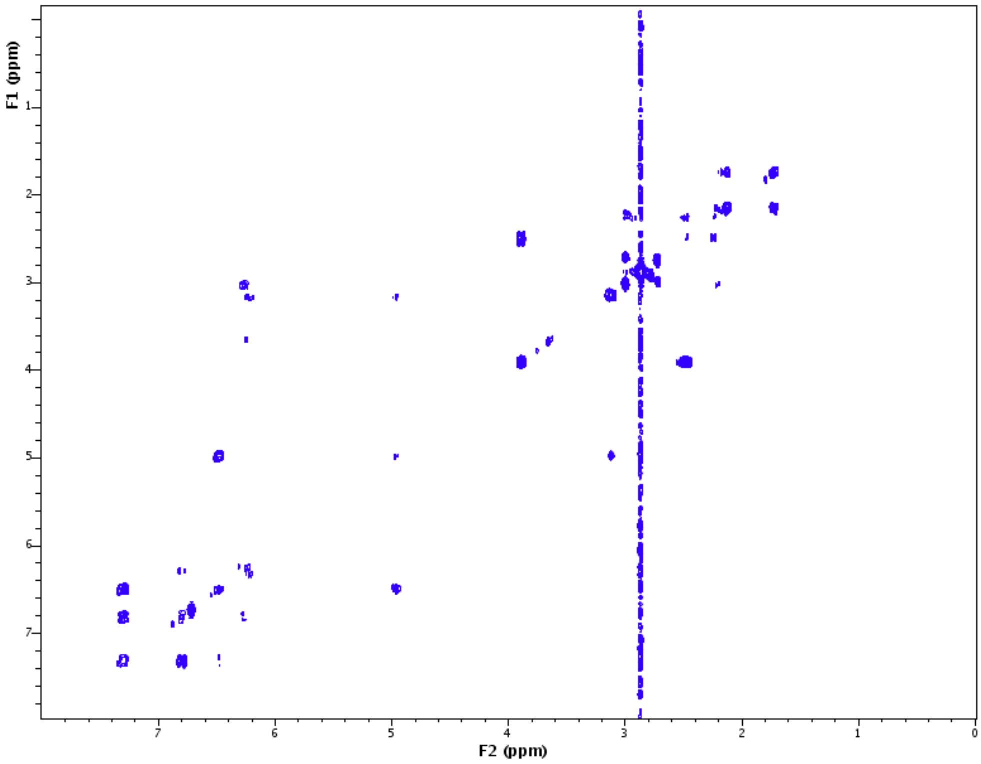

Figure S23. gCOSY NMR spectrum (500 MHz, 75% CH_3CN/D_2O) of compound eluting at 4.45 min (**16**) (*C. retroflexa*).

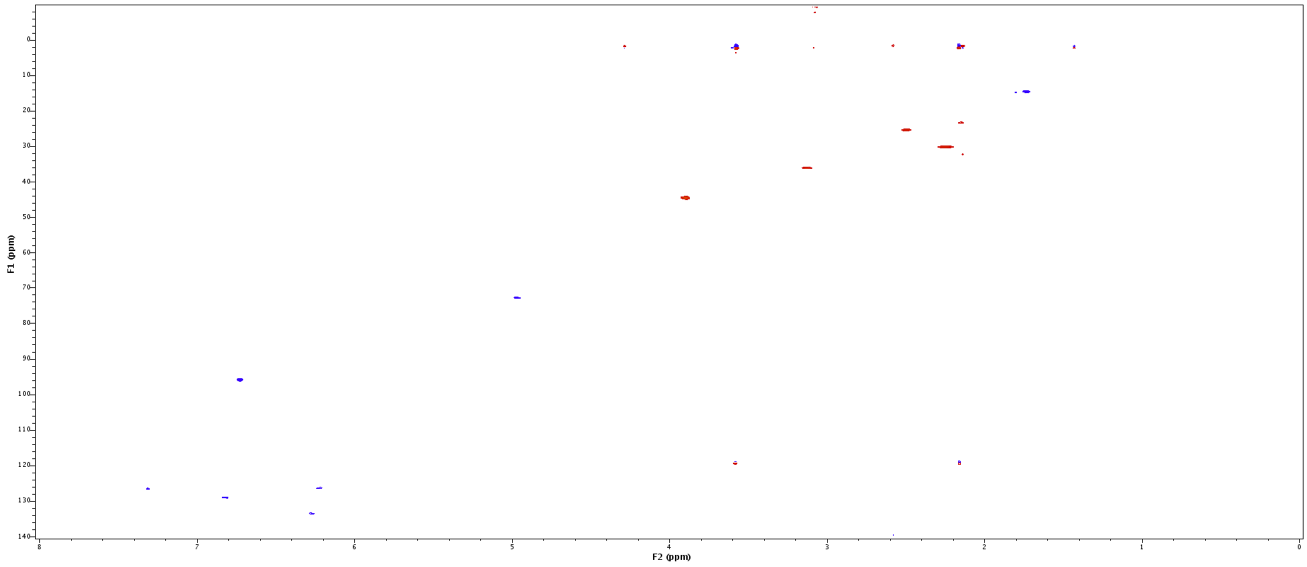

Figure S24. HSQCAD NMR spectrum (500 MHz, 75% CH_3CN/D_2O) of compound eluting at 4.45 min (**16**) (*C. retroflexa*).

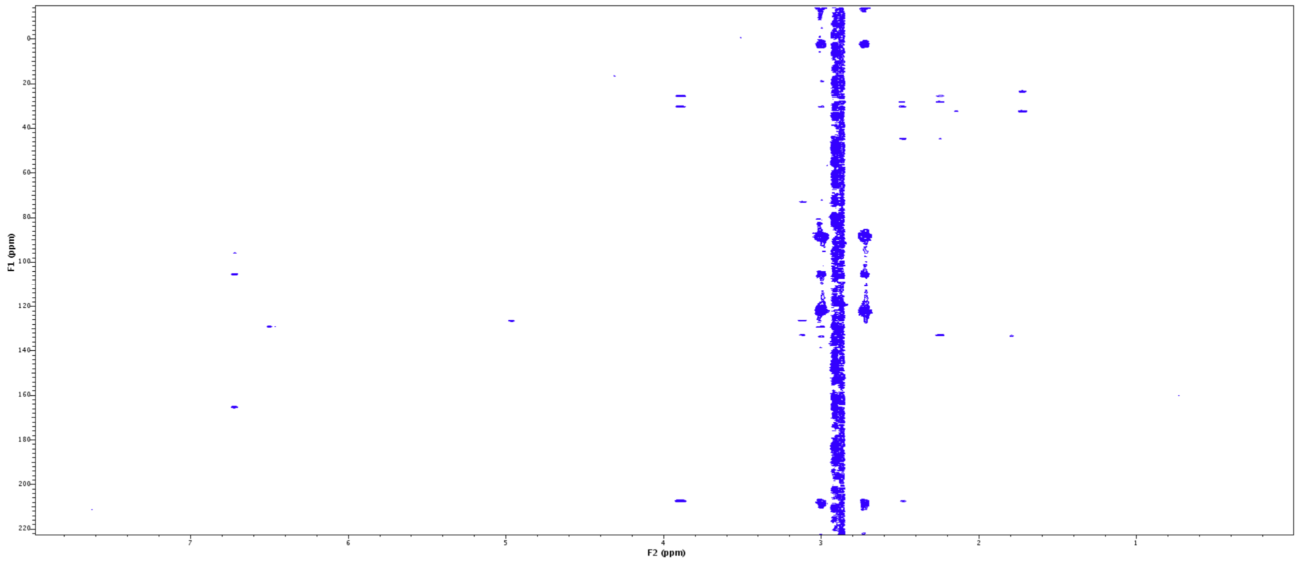

Figure S25. gHMBCAD NMR spectrum (500 MHz, 75% CH_3CN/D_2O) of compound eluting at 4.45 min (**16**) (*C. retroflexa*).

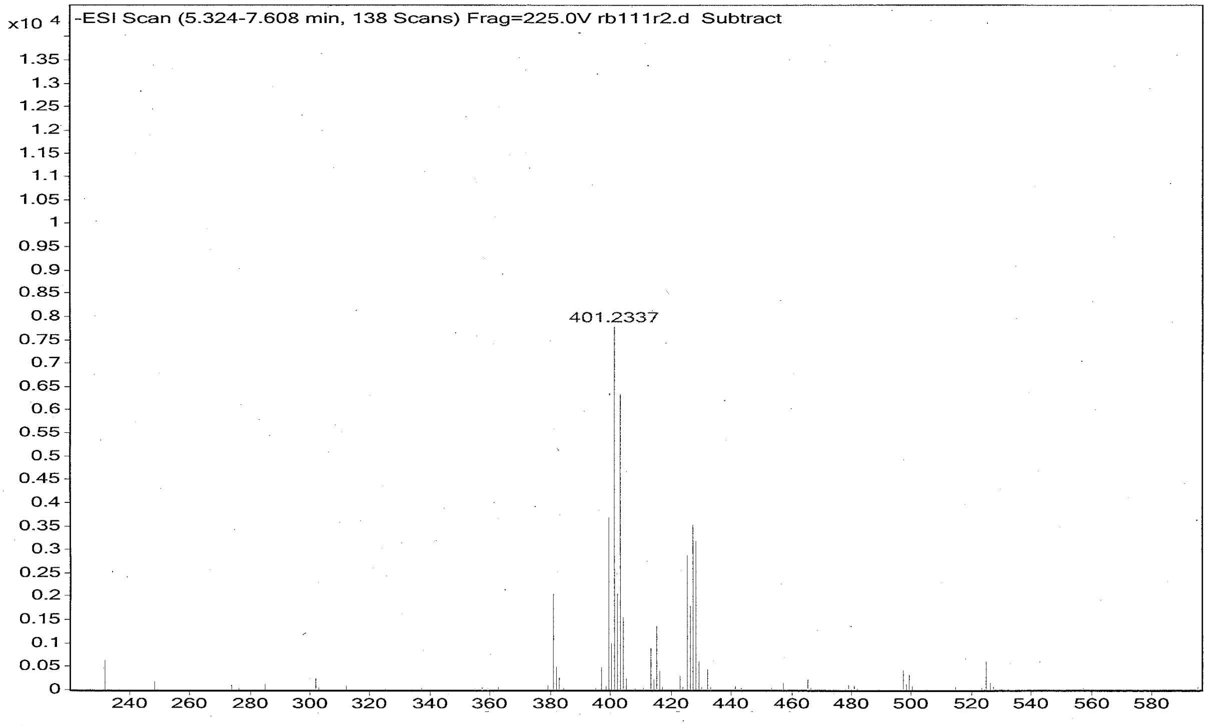

Figure S26. High resolution negative ESI-MS of compound eluting at 4.45 min (**16**) from HPLC-MS (*C. retroflexa*).

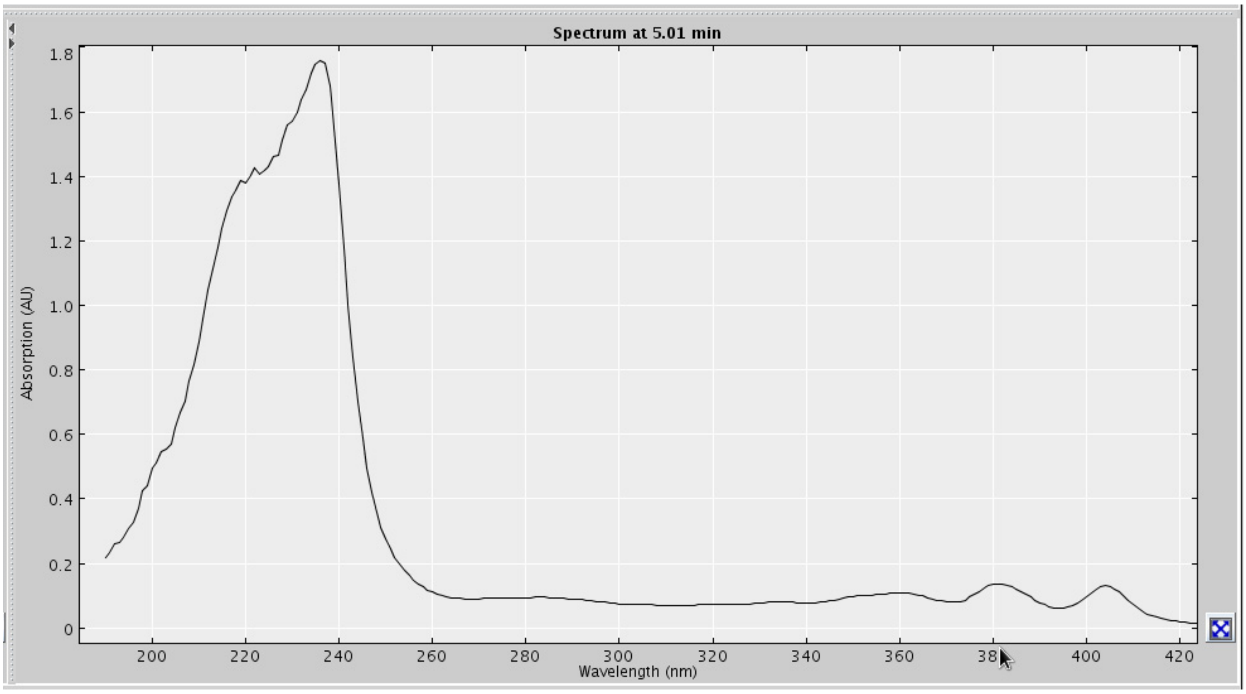

Figure S27. Extracted UV profile of compound eluting at 5.00 min from HPLC-NMR (*Laurencia* sp.).

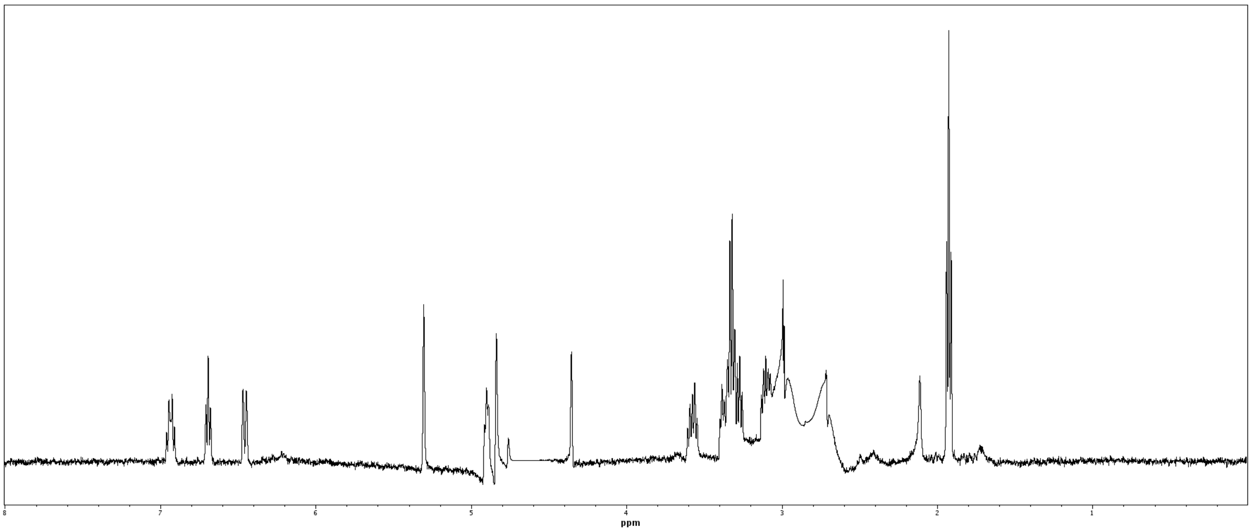

Figure S28. WET1D Proton NMR spectrum (500 MHz, 75% CH_3CN/D_2O) of compound eluting at 5.00 min (*Laurencia* sp.).

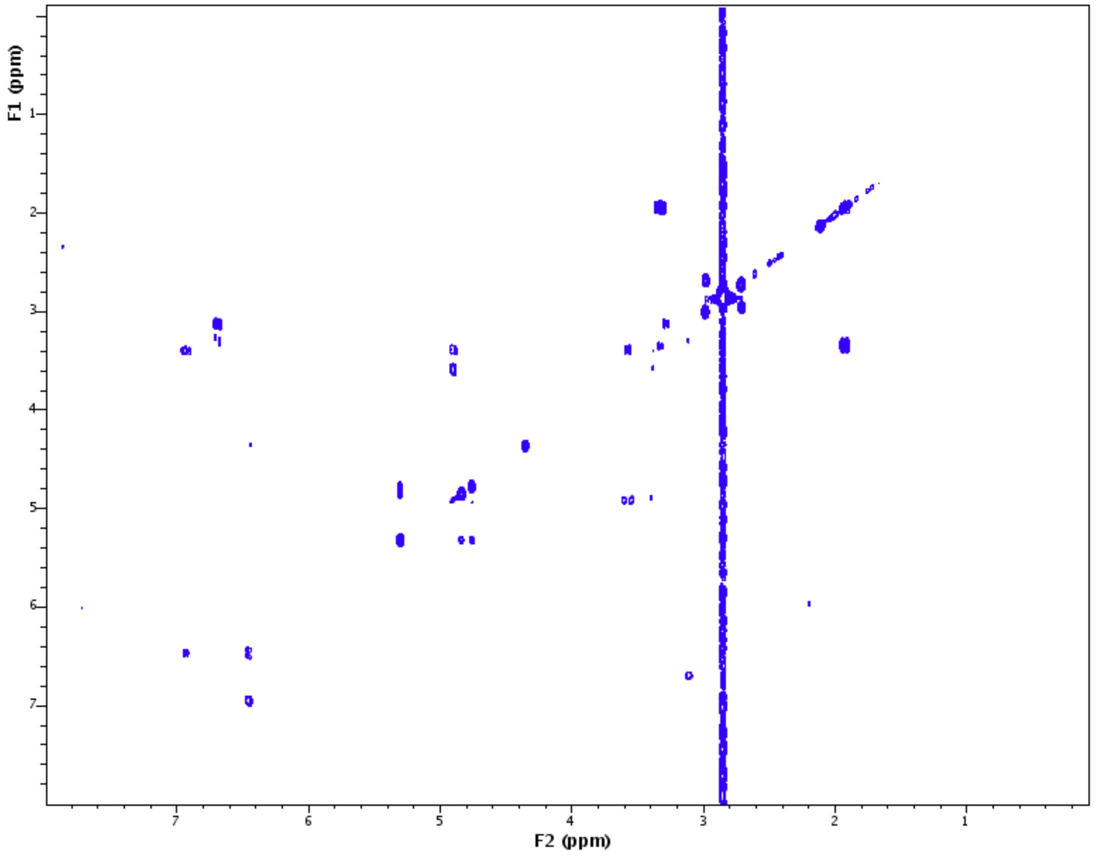

Figure S29. gCOSY NMR spectrum (500 MHz, 75% CH_3CN/D_2O) of compound eluting at 5.00 min (*Laurencia* sp.).

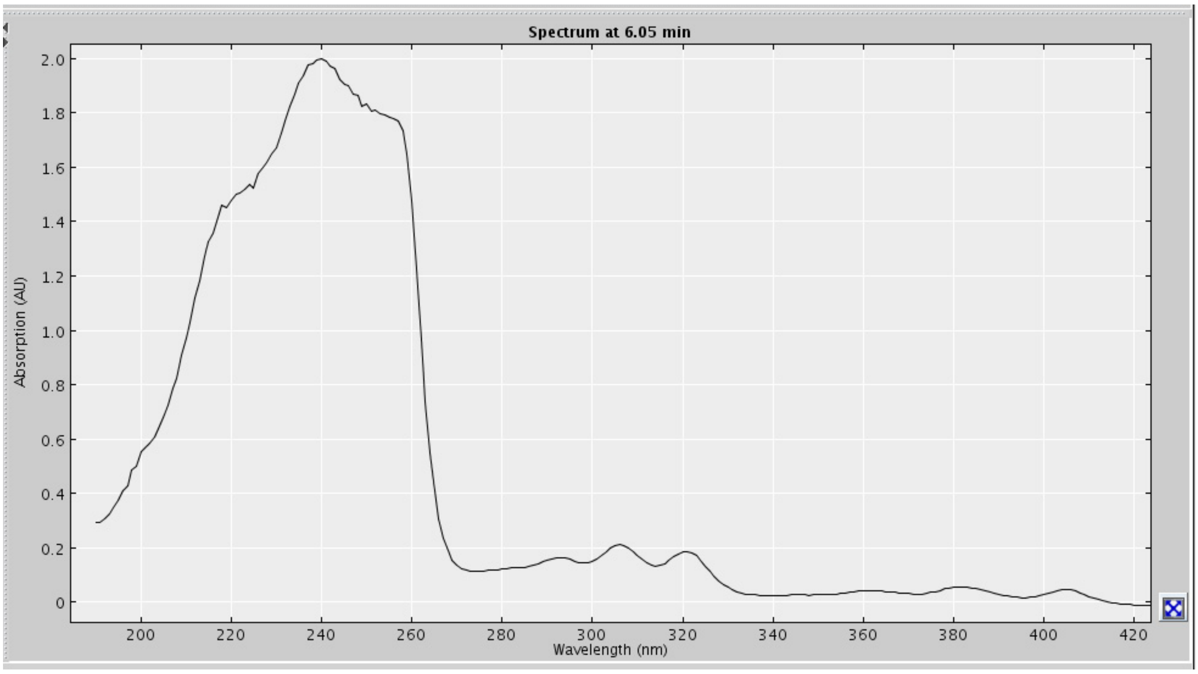

Figure S30. Extracted UV profile of compound eluting at 6.05 min from HPLC-NMR (*Laurencia* sp.).

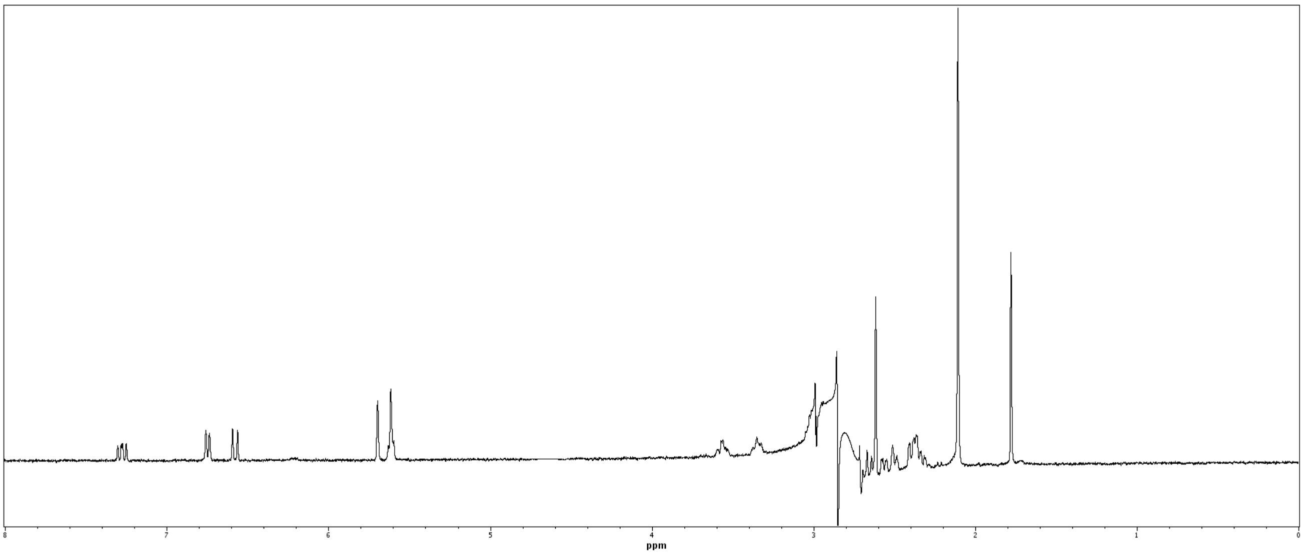

Figure S31. WET1D Proton NMR spectrum (500 MHz, 75% CH_3CN/D_2O) of compound eluting at 6.05 min (*Laurencia* sp.).

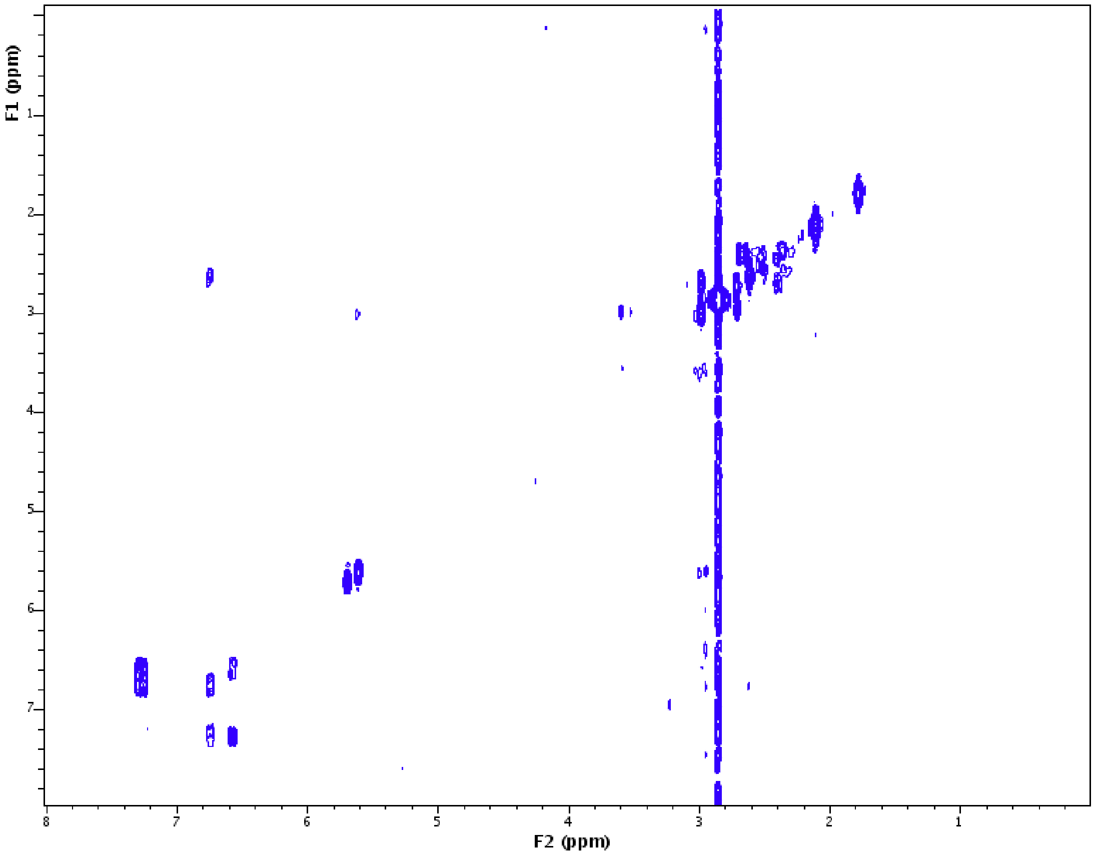

Figure S32. gCOSY NMR spectrum (500 MHz, 75% CH_3CN/D_2O) of compound eluting at 6.05 min (*Laurencia* sp.).

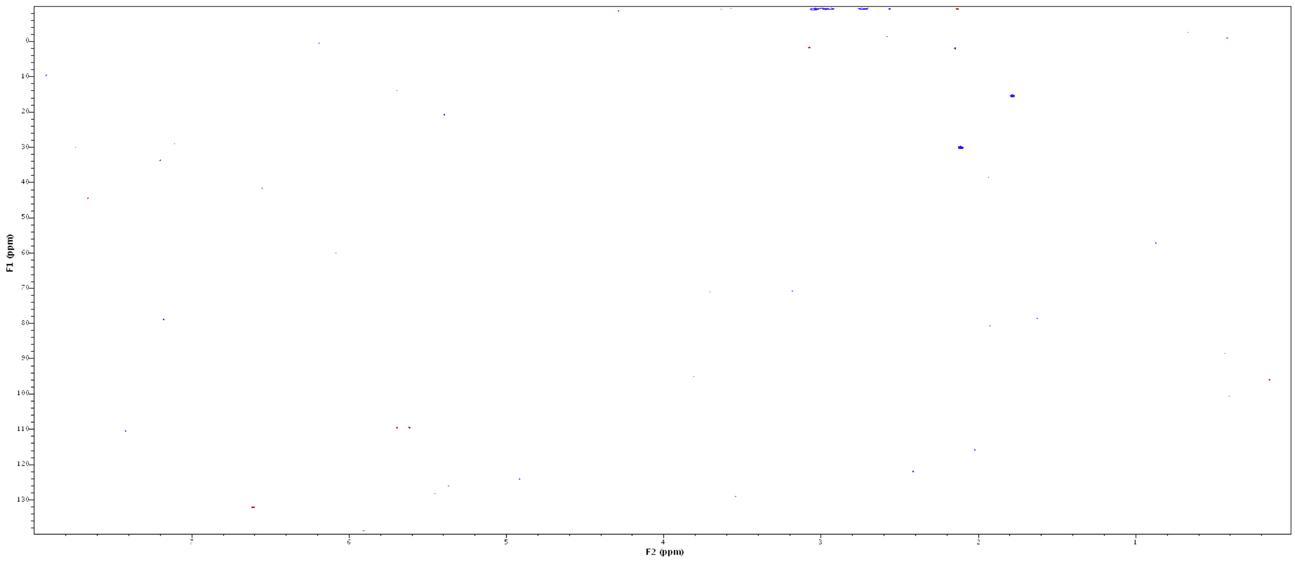

Figure S33. HSQCAD NMR spectrum (500 MHz, 75% CH_3CN/D_2O) of compound eluting at 6.05 min (*Laurencia* sp.).

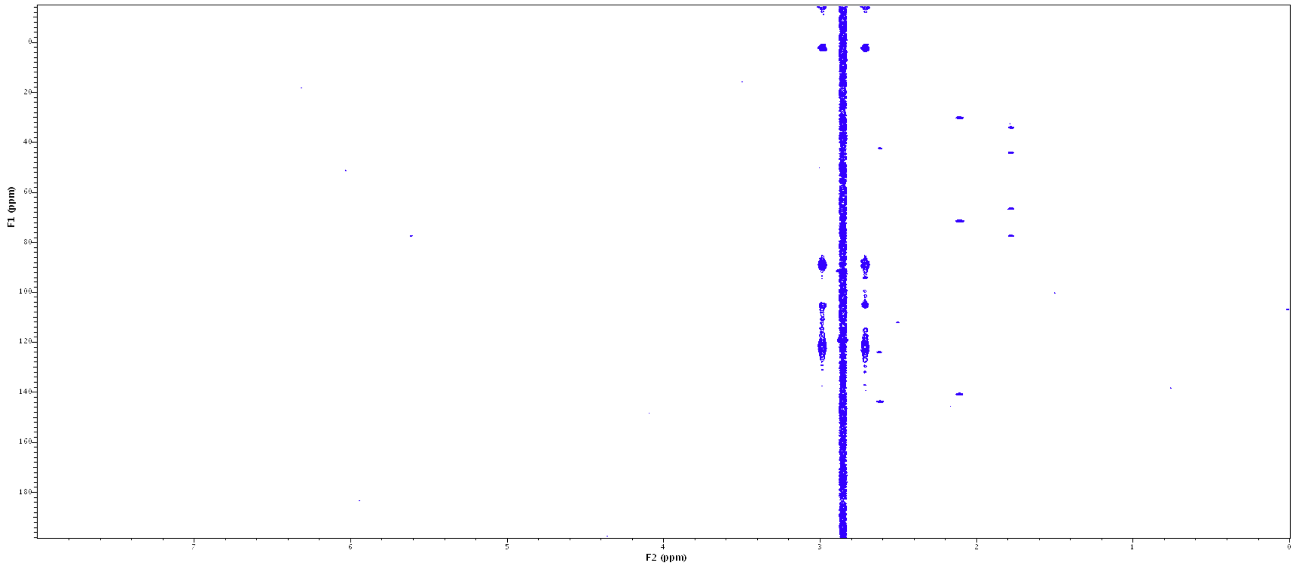

Figure S34. gHMBCAD NMR spectrum (500 MHz, 75% CH_3CN/D_2O) of compound eluting at 6.05 min (*Laurencia* sp.).

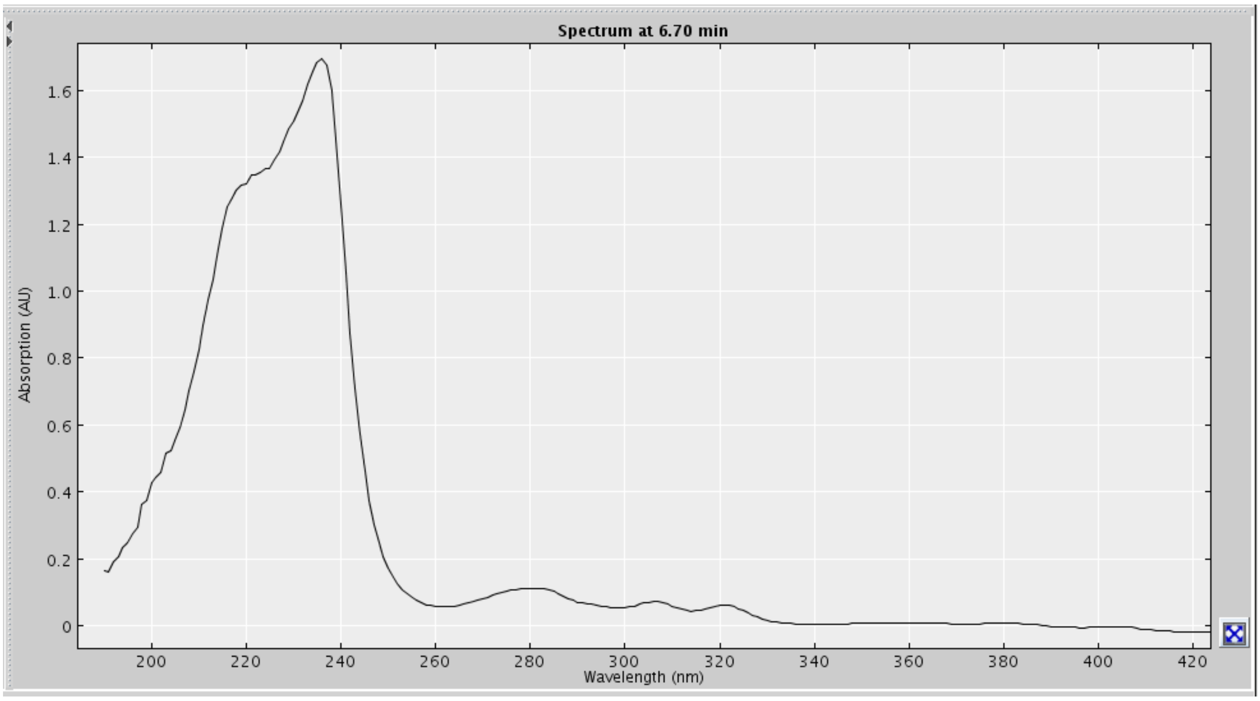

Figure S35. Extracted UV profile of compound eluting at 6.70 min from HPLC-NMR (*Laurencia* sp.).

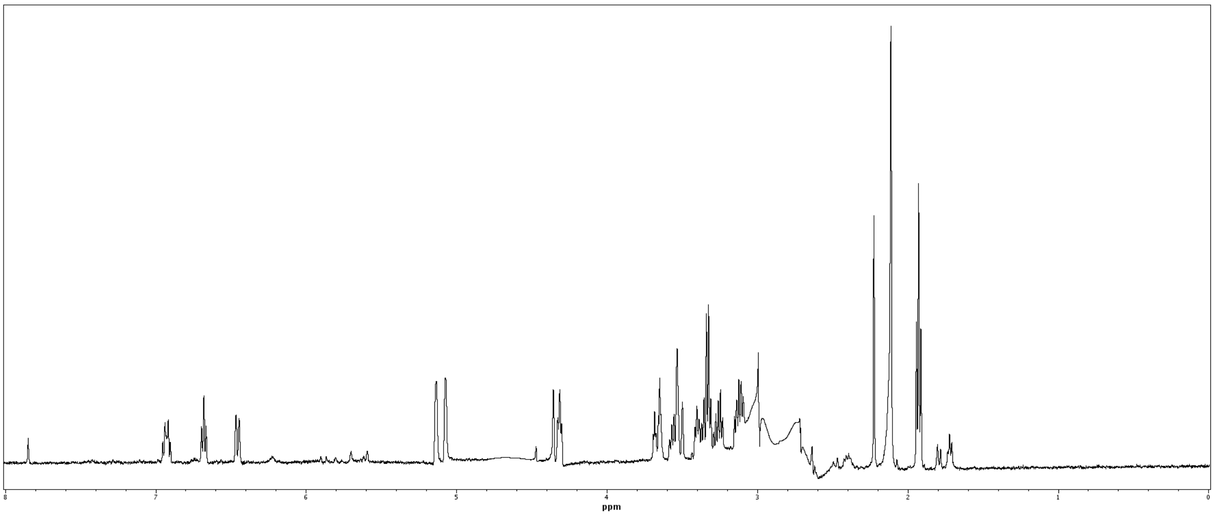

Figure S36. WET1D Proton NMR spectrum (500 MHz, 75% CH_3CN/D_2O) of compound eluting at 6.70 min (*Laurencia* sp.).

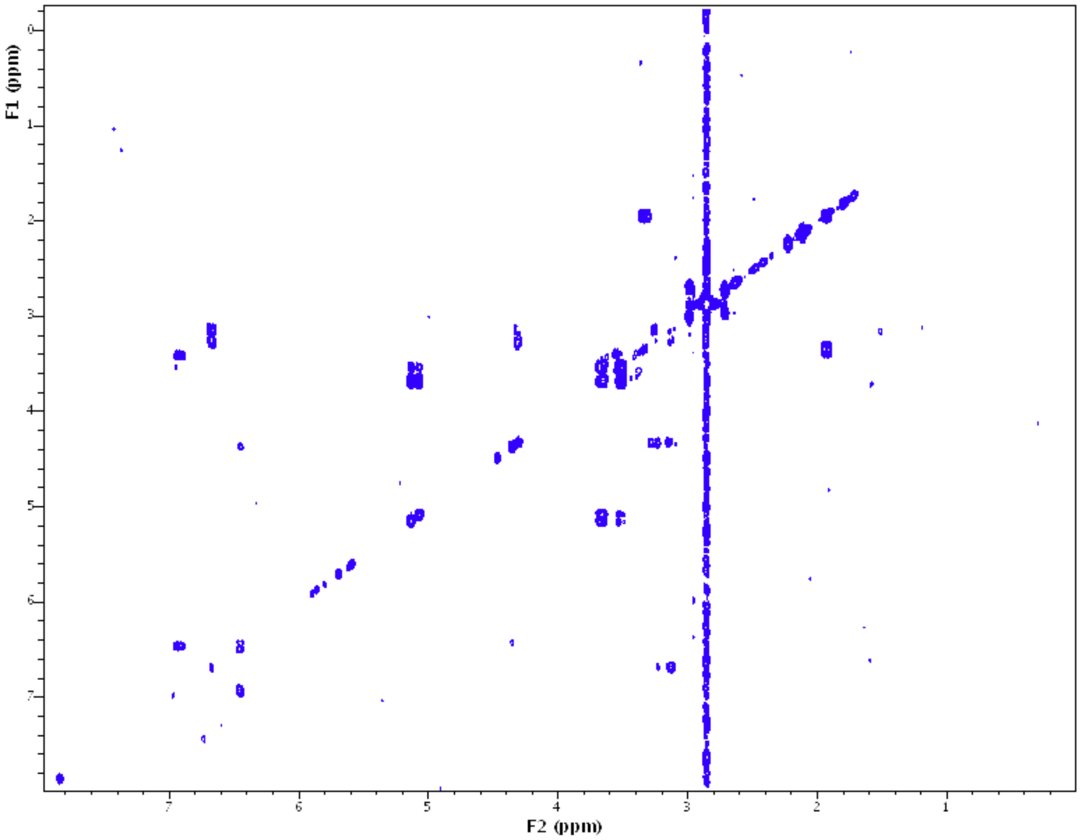

Figure S37. gCOSY NMR spectrum (500 MHz, 75% CH_3CN/D_2O) of compound eluting at 6.70 min (*Laurencia* sp.).

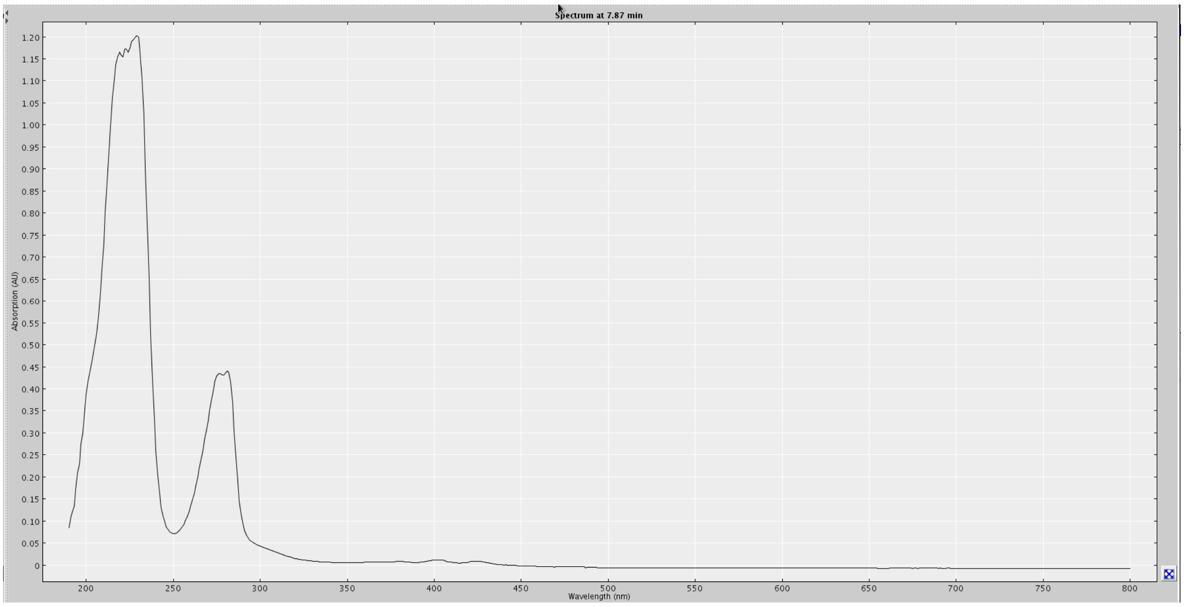

Figure S38. Extracted UV profile of compound eluting at 7.87 min (**4**) from HPLC-NMR (*S. decipiens*).

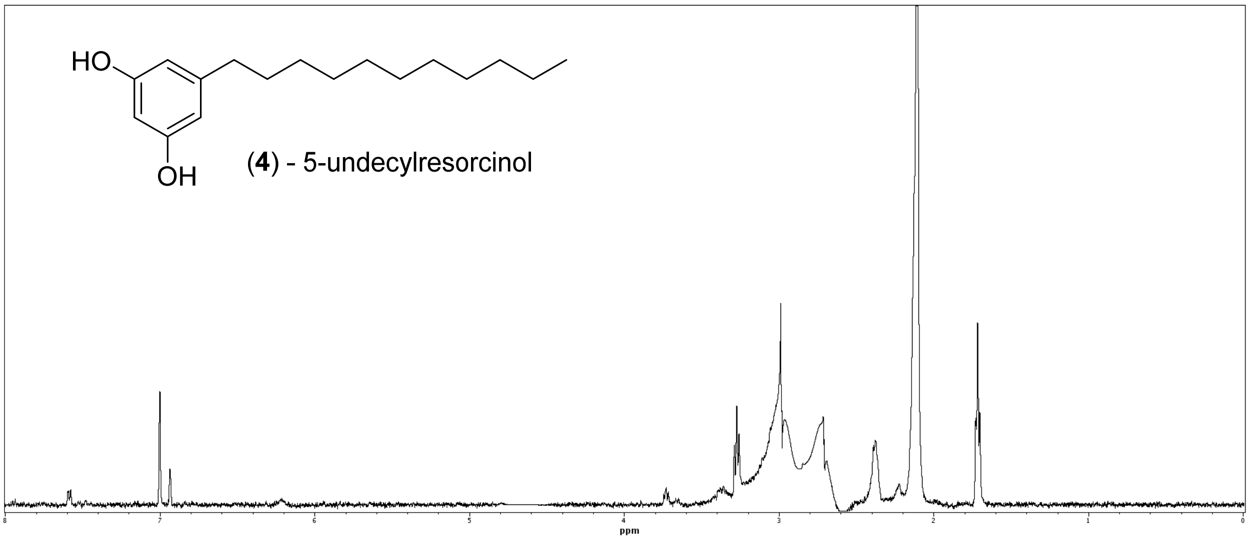

Figure S39. WET1D Proton NMR spectrum (500 MHz, 75% CH_3CN/D_2O) of compound eluting at 7.87 min (**4**) (*S. decipiens*).

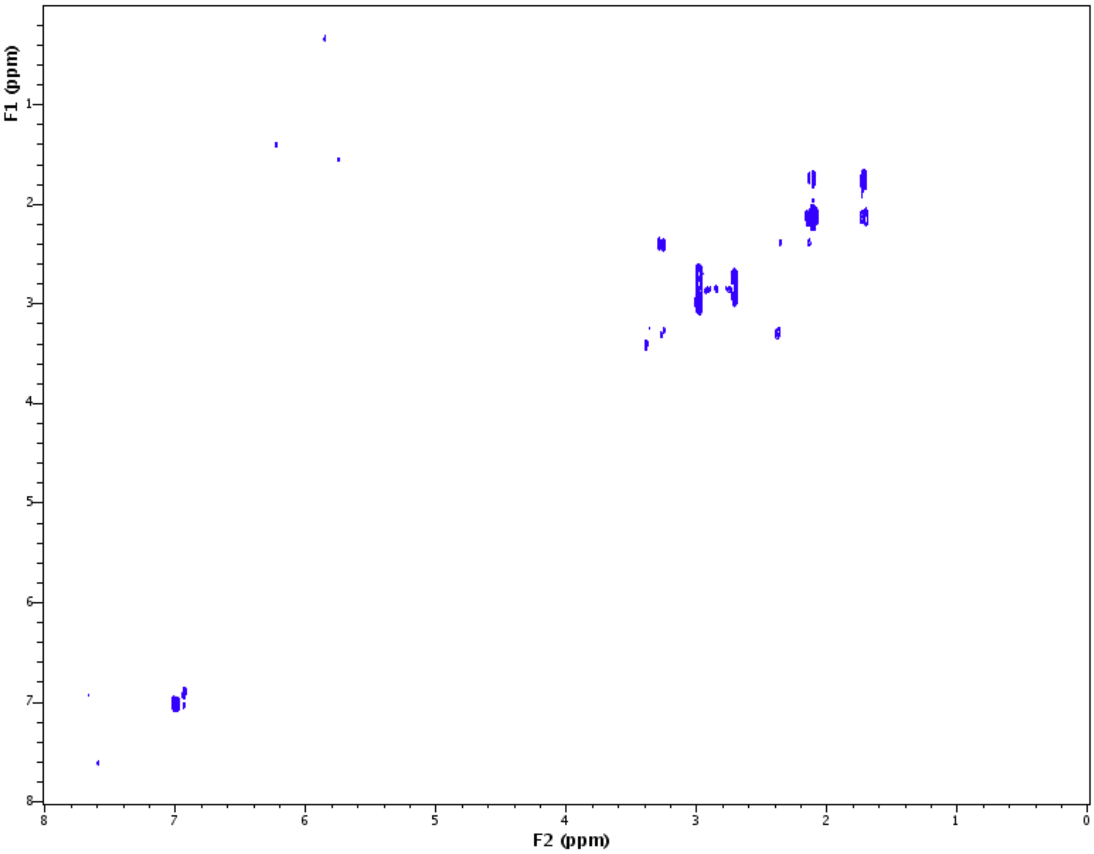

Figure S40. gCOSY NMR spectrum (500 MHz, 75% CH_3CN/D_2O) of compound eluting at 7.87 min (**4**) (*S. decipiens*).

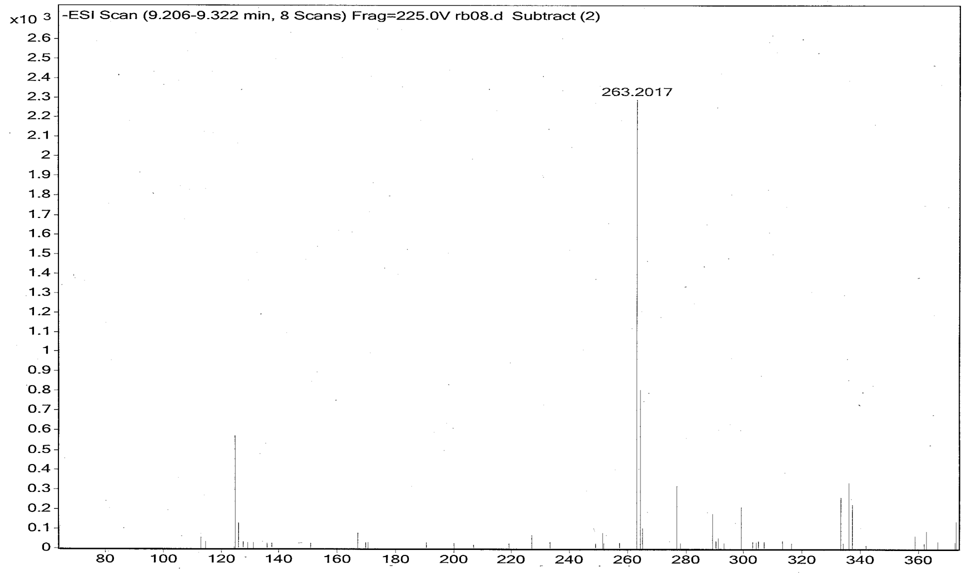

Figure S41. High resolution negative ESI-MS of compound eluting at 7.87 min (**4**) from HPLC-MS (*S. decipiens*).

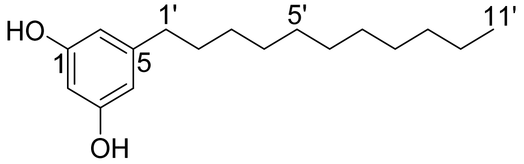

(**4**) - 5-undecylresorcinol

Position	δ_H (*J* in Hz)	gCOSY
1		
2	6.93, s	
3		
4	7.00, s	
5		
6	7.00, s	
1′	3.27, t (7.5)	2′
2′	2.38, m	1′
3′	2.11, m	
4′	2.11, m	
5′	2.11, m	
6′	2.11, m	
7′	2.11, m	
8′	2.11, m	
9′	2.11, m	
10′	2.11, m	11′
11′	1.72, t (6.5)	10′
1-OH	ND	
3-OH	ND	

Referenced to 75% CH_3CN/D_2O; ND Not Detected.

Figure S42. NMR data for compound eluting at 7.87 min (**4**) (*S. decipiens*).

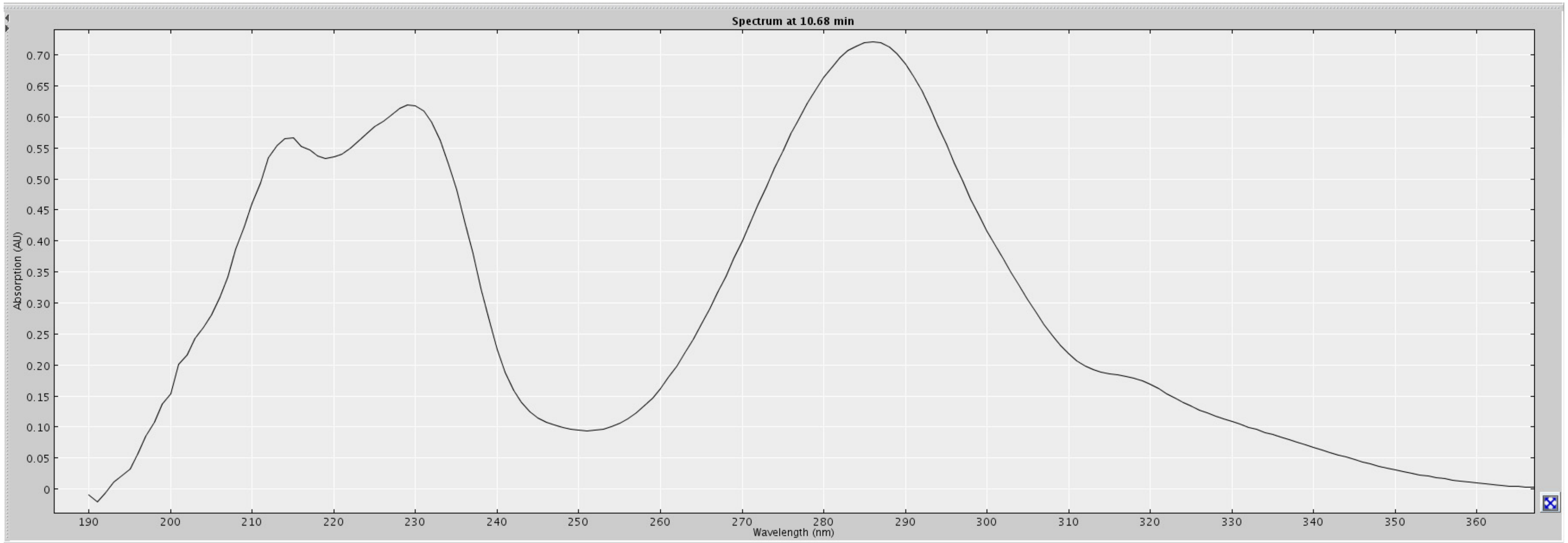

Figure S43. Extracted UV profile of compound eluting at 9.98 min (**12**) from HPLC-NMR (*C. retroflexa*).

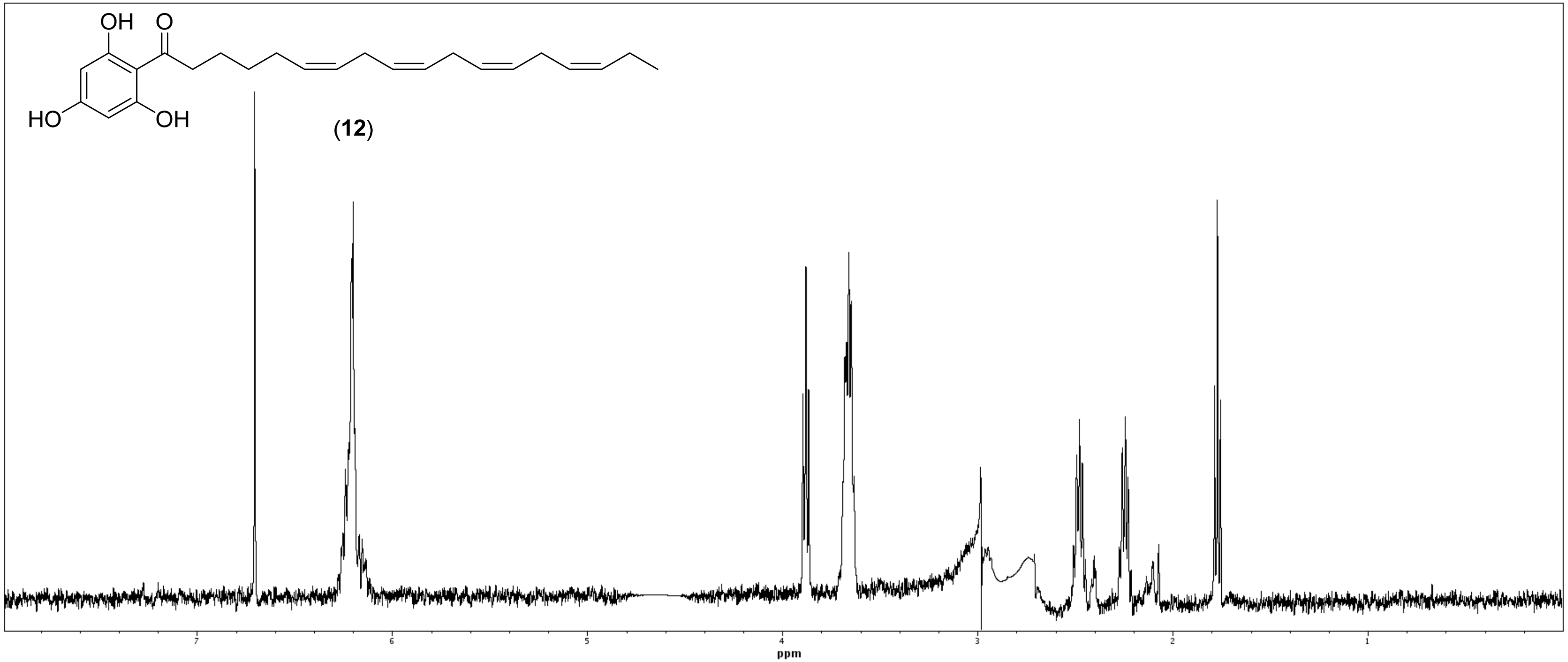

Figure S44. WET1D Proton NMR spectrum (500 MHz, 75% CH_3CN/D_2O) of compound eluting at 9.98 min (**12**) (*C. retroflexa*).

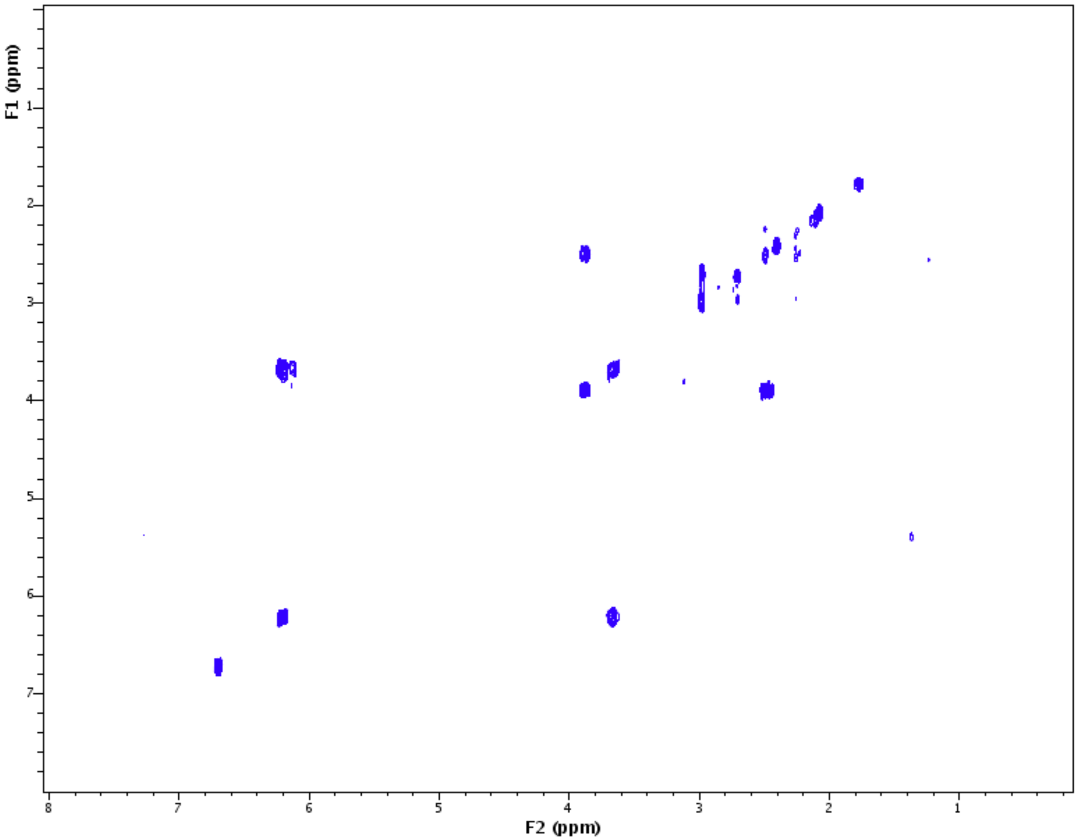

Figure S45. gCOSY NMR spectrum (500 MHz, 75% CH_3CN/D_2O) of compound eluting at 9.98 min (**12**) (*C. retroflexa*).

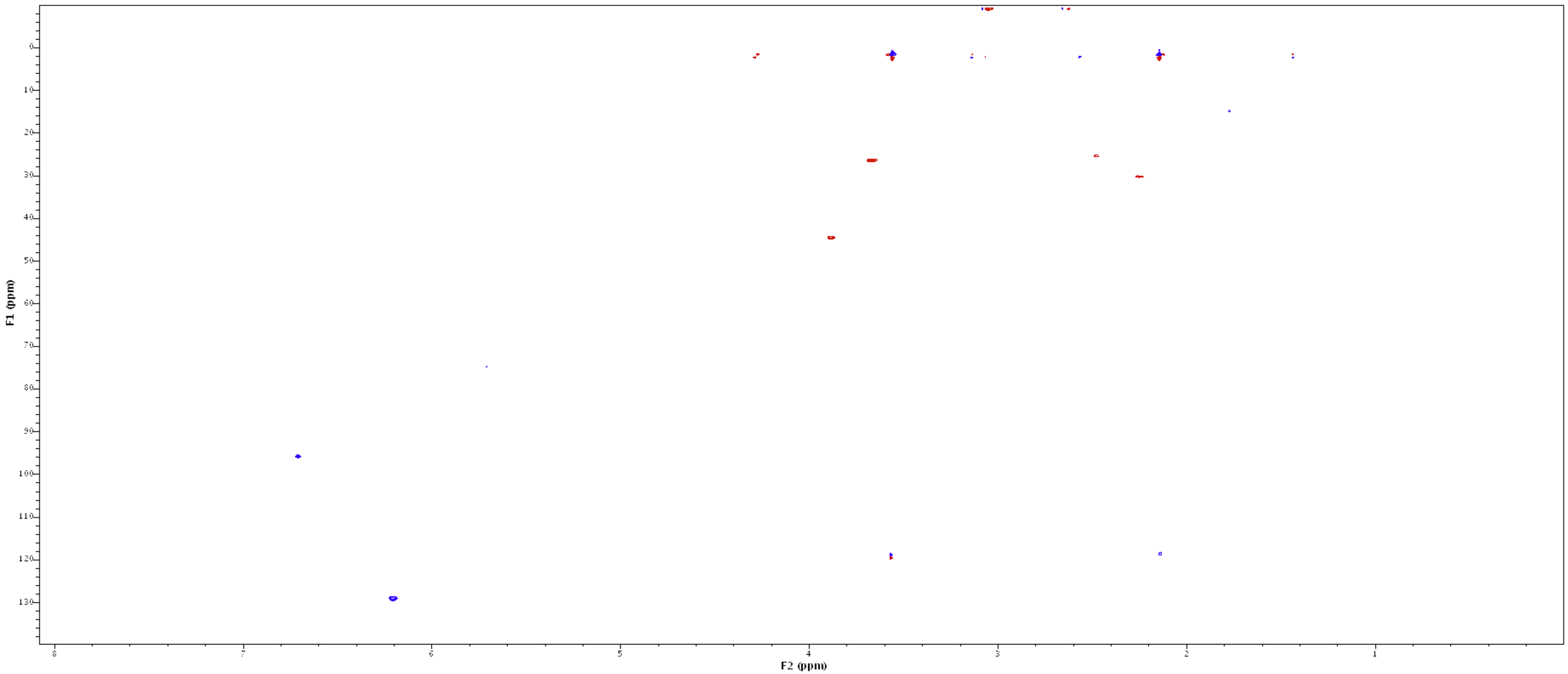

Figure S46. HSQCAD NMR spectrum (500 MHz, 75% CH_3CN/D_2O) of compound eluting at 9.98 min (**12**) (*C. retroflexa*).

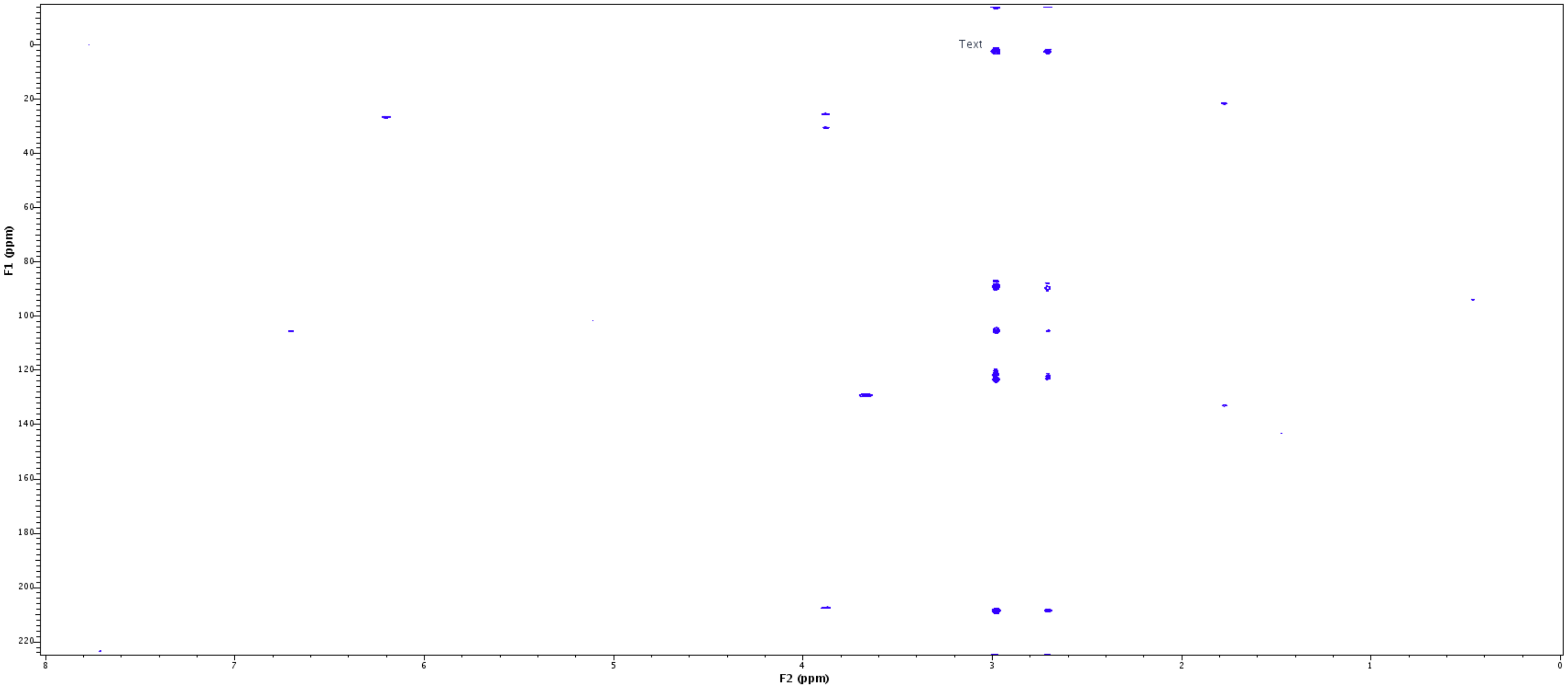

Figure S47. gHMBCAD NMR spectrum (500 MHz, 75% CH_3CN/D_2O) of compound eluting at 9.98 min (**12**) (*C. retroflexa*).

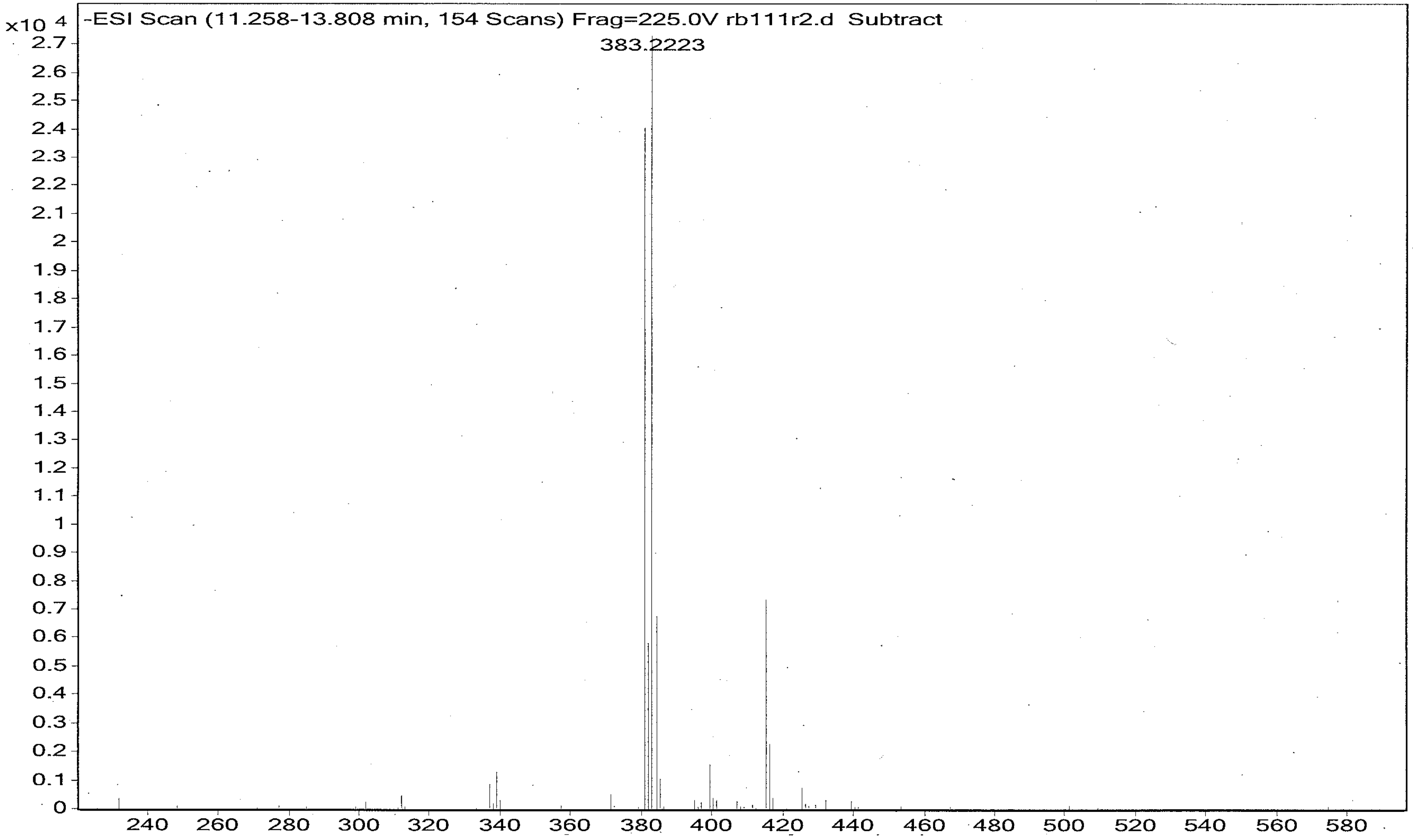

Figure S48. High resolution negative ESI-MS of compound eluting at 9.98 min (**12**) from HPLC-MS (*C. retroflexa*).

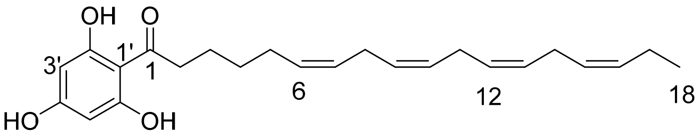

(**12**)

Position	δ_H (*J* in Hz)	δ_C, mult.[a]	gCOSY	gHMBCAD
1		207.2, s		
2	3.87, t (7.5)	44.3, t	3	1, 3, 4
3	2.46, p (7.5)	25.1, t	2, 4	5 [w]
4	2.23, p (7.5)	30.0, t	3	
5	SS	30.4, t		
6	6.18–6.28, m	128.9, d		8
7	6.18–6.28, m	128.9, d	8	
8	3.64, m	26.2, t	7, 9	6, 10
9	6.18–6.28, m	128.9, d	8	11
10	6.18–6.28, m	128.9, d	11	8
11	3.64, m	26.2, t	10, 12	9, 13
12	6.18–6.28, m	128.9, d	11	14
13	6.18–6.28, m	128.9, d	14	11
14	3.64, m	26.2, t	13, 15	12
15	6.18–6.28, m	128.9, d	14	
16	6.18–6.28, m	132.7, d		14
17	SS	21.1, t		
18	1.76, t (7.0)	14.6, q		16, 17
1′		105.0, s		
2′		164.9, s		
3′	6.69, s	95.7, d		1′, 2′, 4′, 6′
4′		164.9, s		
5′	6.69, s	95.7, d		
6′		164.9, s		
2′-OH	ND			
4′-OH	ND			
6′-OH	ND			

Referenced to D_2O (δ_H 4.64 ppm); [a] carbon assignments based on HSQCAD and gHMBCAD NMR experiments; [w] indicates weak or long range correlation; SS Signal suppressed; ND Not Detected.

Figure S49. NMR data for compound eluting at 9.98 min (12) (*C. retroflexa*).

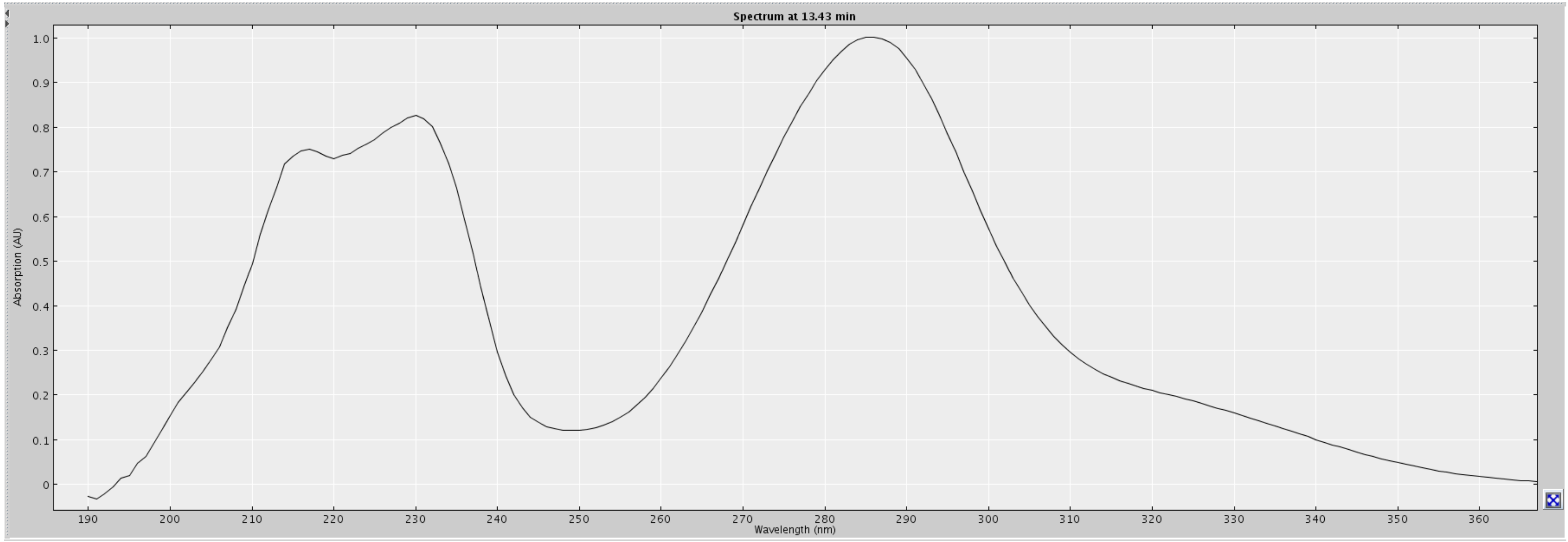

Figure S50. Extracted UV profile of compound eluting at 12.95 min (**13**) from HPLC-NMR (*C. retroflexa*).

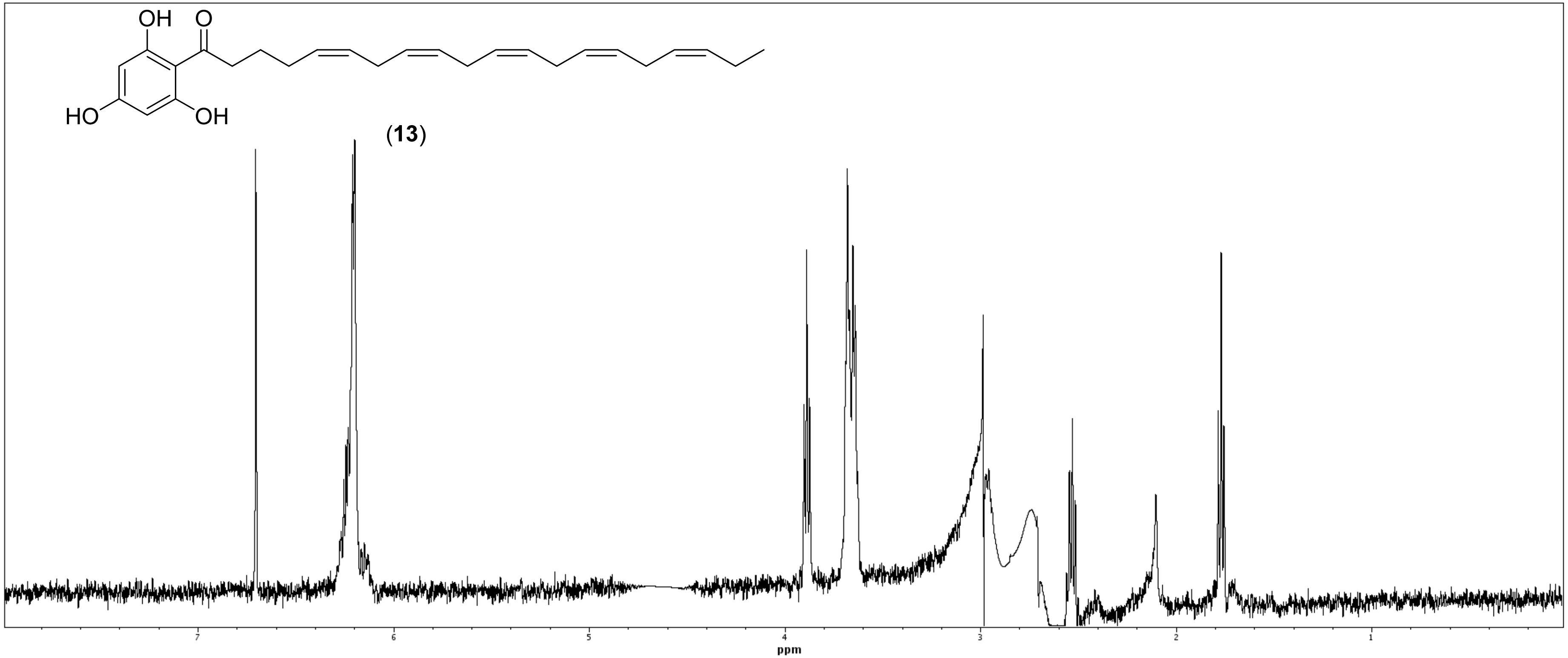

Figure S51. WET1D Proton NMR spectrum (500 MHz, 75% CH_3CN/D_2O) of compound eluting at 12.95 min (**13**) (*C. retroflexa*).

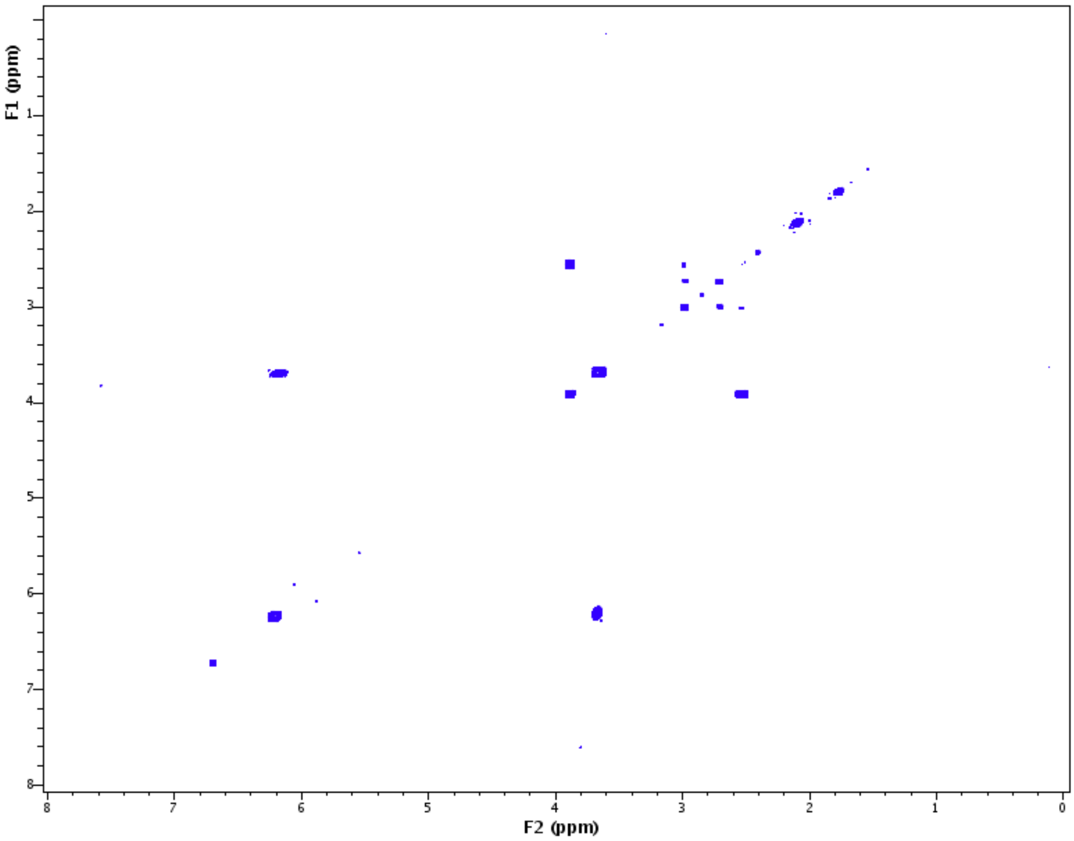

Figure S52. gCOSY NMR spectrum (500 MHz, 75% CH_3CN/D_2O) of compound eluting at 12.95 min (**13**) (*C. retroflexa*).

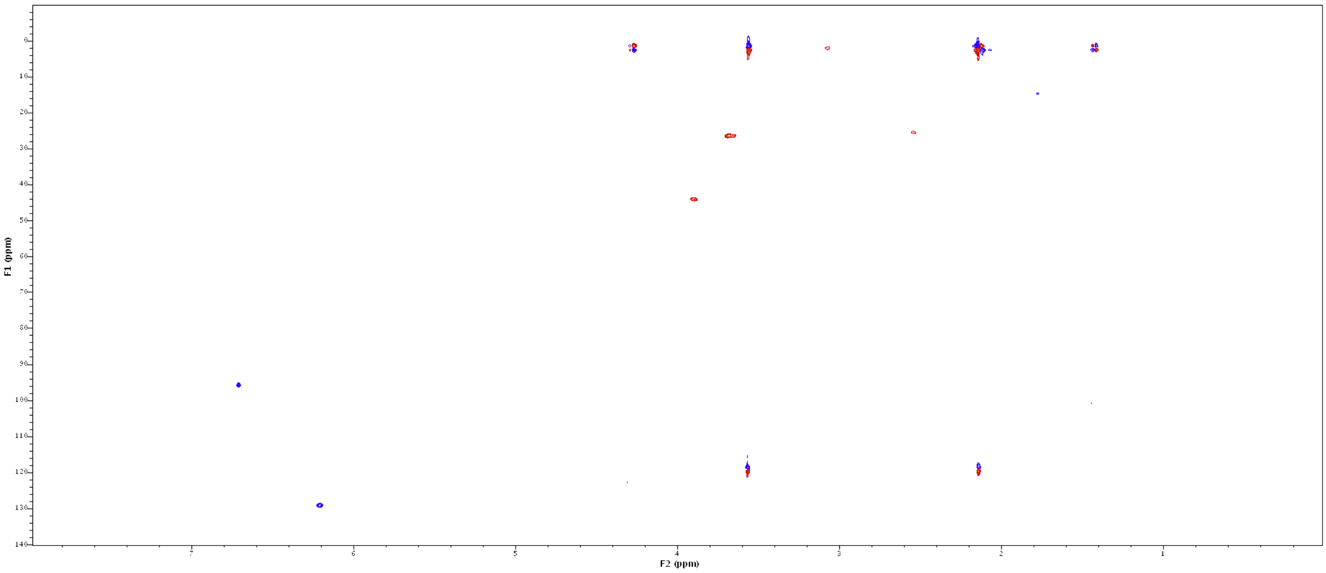

Figure S53. HSQCAD NMR spectrum (500 MHz, 75% CH_3CN/D_2O) of compound eluting at 12.95 min (**13**) (*C. retroflexa*).

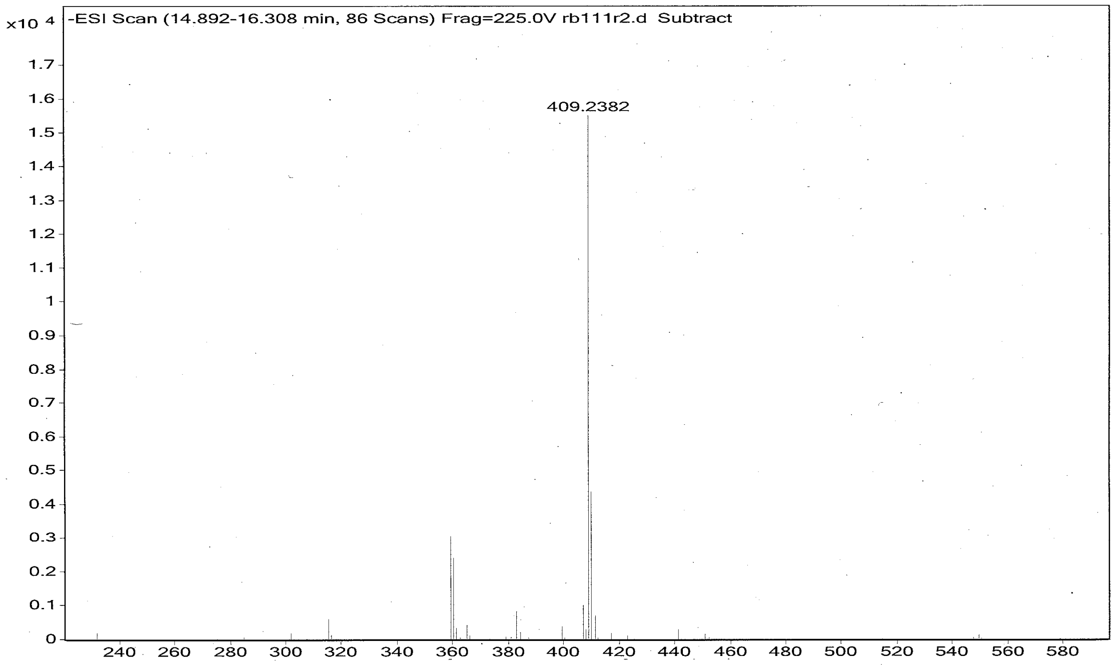

Figure S54. High resolution negative ESI-MS of compound eluting at 12.95 min (**13**) from HPLC-MS (*C. retroflexa*).

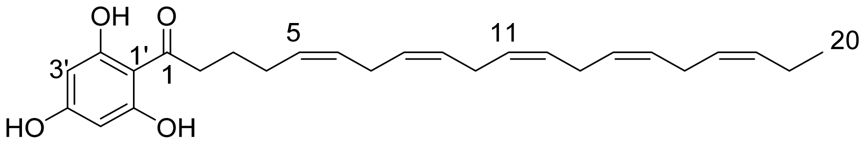

(**13**)

Position	δ_H (*J* in Hz)	δ_C, mult.[a]	gCOSY
1			
2	3.88, t (7.0)	43.8, t	3
3	2.55, m	25.3, t	2, 4
4	3.00, m	ND	3
5	6.19, m	128.9, d	
6	6.19, m	128.9, d	7
7	3.60–3.70, m	26.2, t	6, 8
8	6.19, m	128.9, d	7
9	6.19, m	128.9, d	10
10	3.60–3.70, m	26.2, t	9, 11
11	6.19, m	128.9, d	10
12	6.19, m	128.9, d	13
13	3.60–3.70, m	26.2, t	12, 14
14	6.19, m	128.9, d	13
15	6.19, m	128.9, d	16
16	3.60–3.70	26.2, t	15, 17
17	6.19, m	128.9, d	16
18	6.19, m	128.9, d	
19	SS	ND	
20	1.76, t (7.5)	14.4, q	
1′		ND	
2′		ND	
3′	6.69, s	95.6, d	
4′		ND	
5′	6.69, s	95.6, d	
6′		ND	
2′-OH	ND		
4′-OH	ND		
6′-OH	ND		

Referenced to D_2O (δ_H 4.64 ppm); [a] carbon assignments based on HSQCAD NMR experiments; SS Signal suppressed; ND Not Detected.

Figure S55. NMR data for compound eluting at 12.95 min (**13**) (*C. retroflexa*).

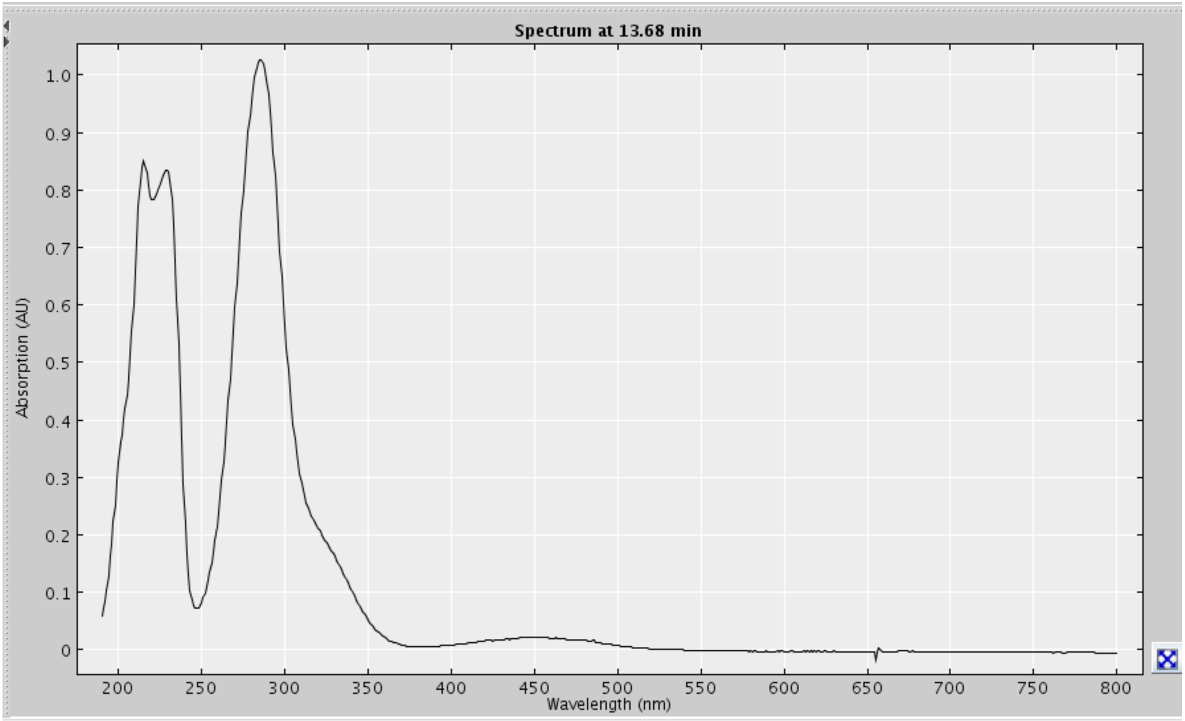

Figure S56. Extracted UV profile of compound eluting at 13.65 min (**17**) from HPLC-NMR (*S. cf. fallax*).

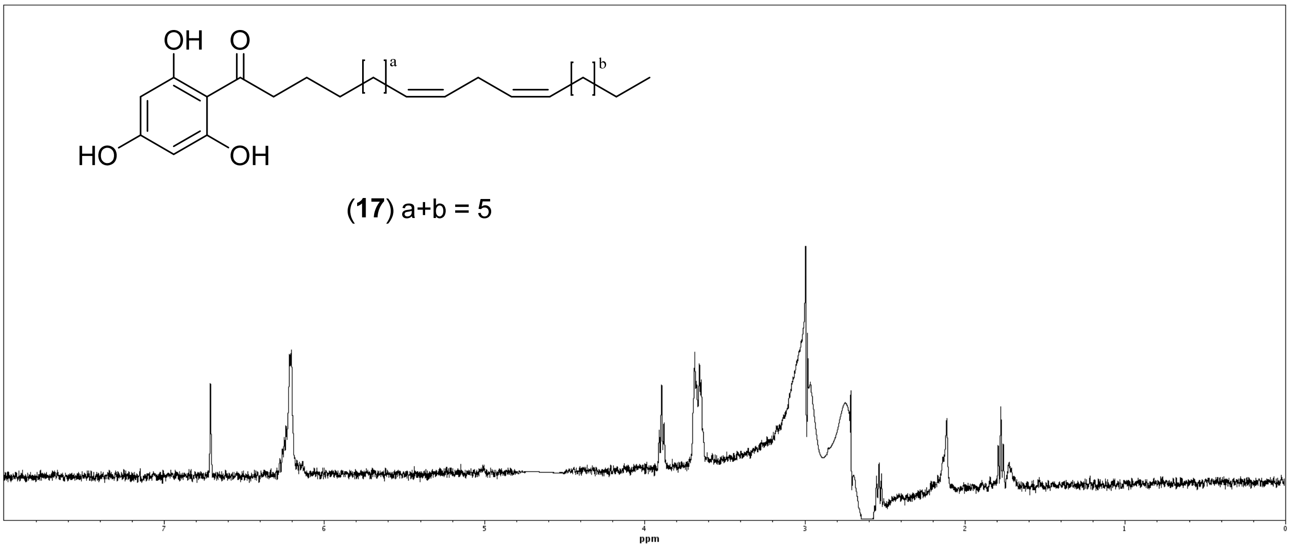

Figure S57. WET1D Proton NMR spectrum (500 MHz, 75% CH_3CN/D_2O) of compound eluting at 13.65 min (**17**) (*S. cf. fallax*).

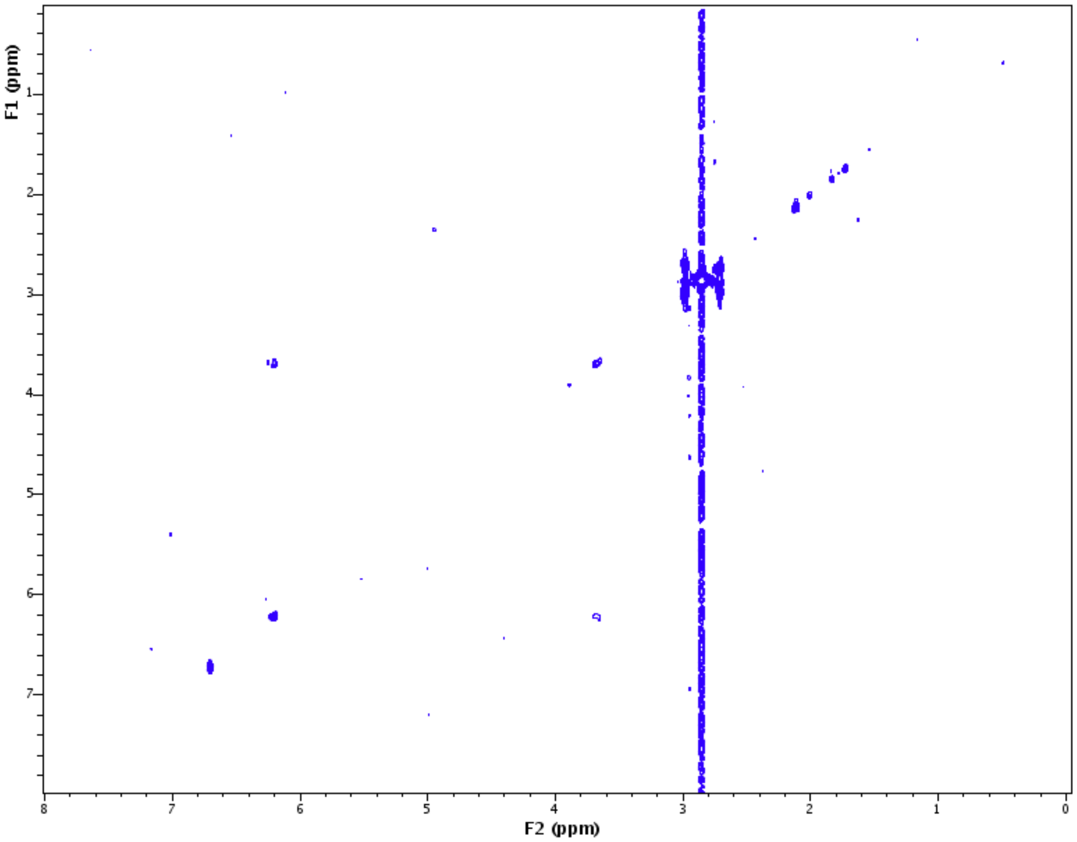

Figure S58. gCOSY NMR spectrum (500 MHz, 75% CH_3CN/D_2O) of compound eluting at 13.65 min (**17**) (*S. cf. fallax*).

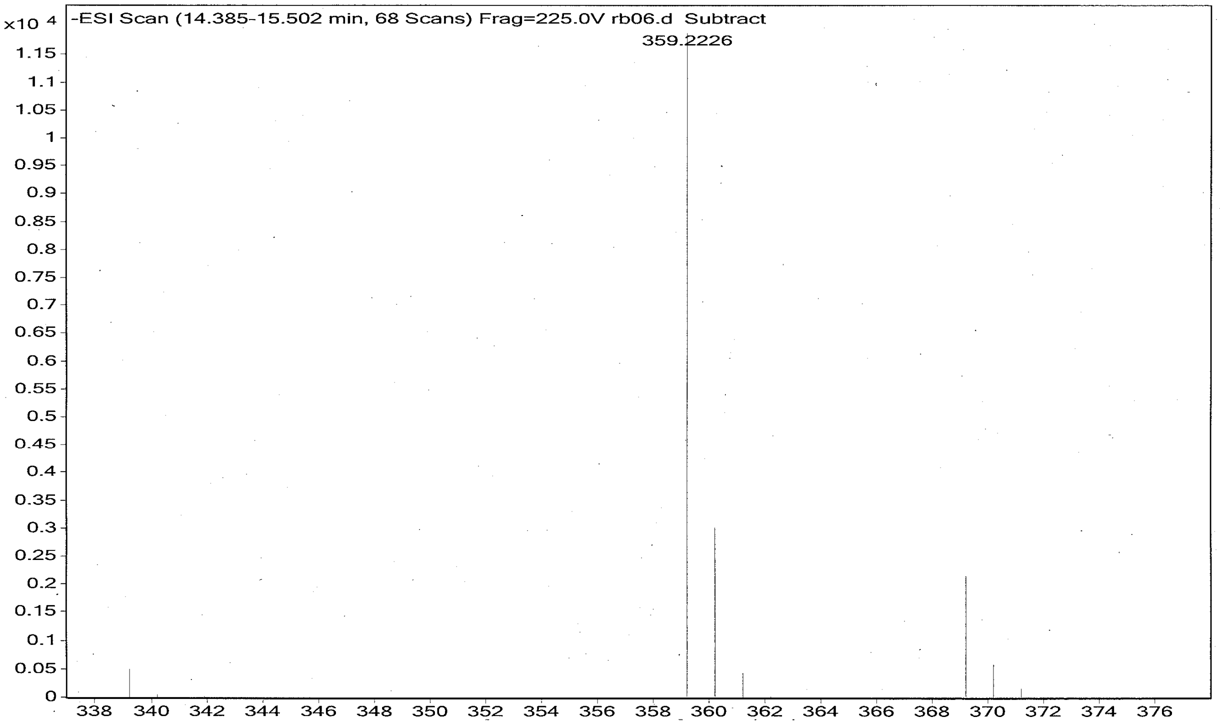

Figure S59. High resolution negative ESI-MS of compound eluting at 13.65 min (**17**) from HPLC-MS (*S. cf. fallax*).

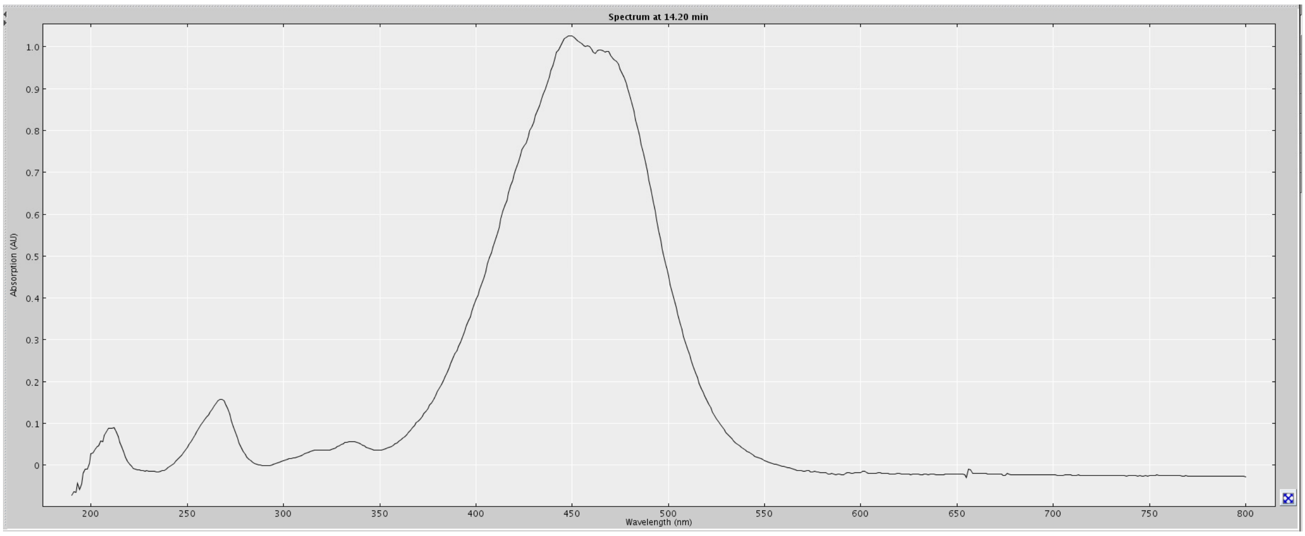

Figure S60. Extracted UV profile of compound eluting at 14.53 min (**5**) from HPLC-NMR (*H. pseudospicata*).

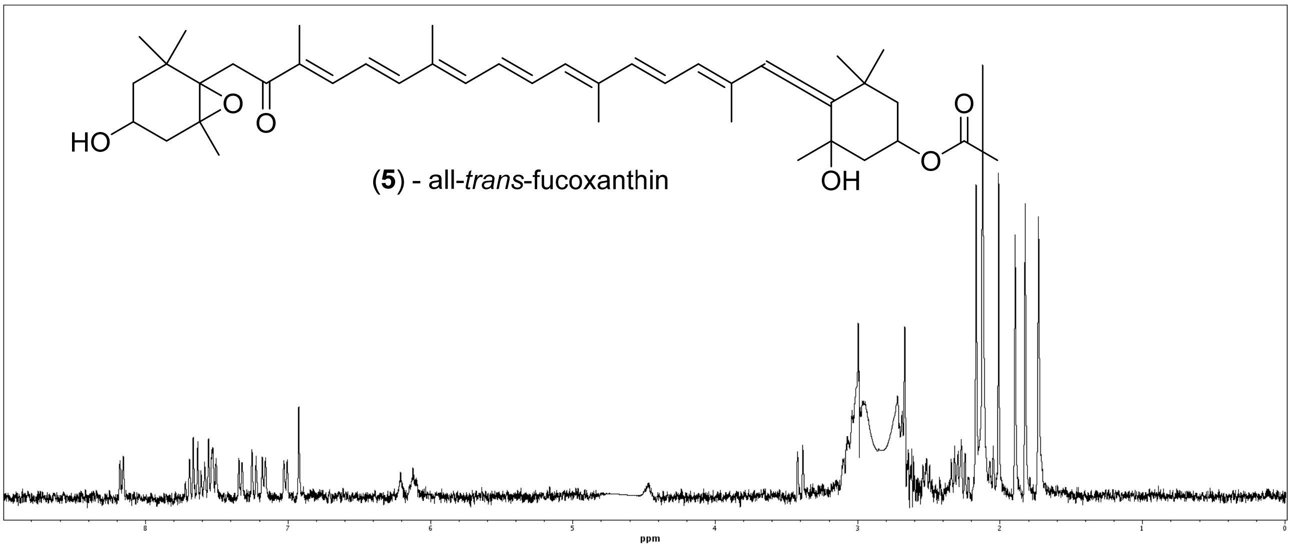

Figure S61. WET1D Proton NMR spectrum (500 MHz, 75% CH_3CN/D_2O) of compound eluting at 14.53 min (**5**) (*H. pseudospicata*).

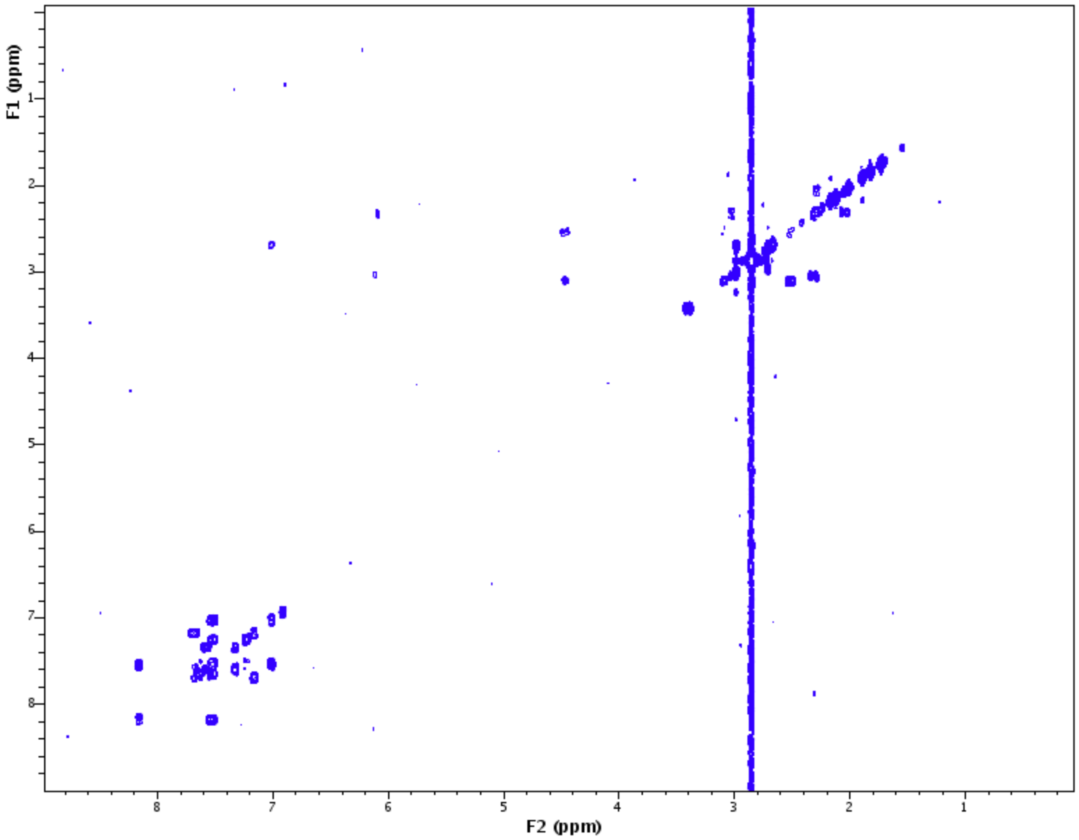

Figure S62. gCOSY NMR spectrum (500 MHz, 75% CH_3CN/D_2O) of compound eluting at 14.53 min (**5**) (*H. pseudospicata*).

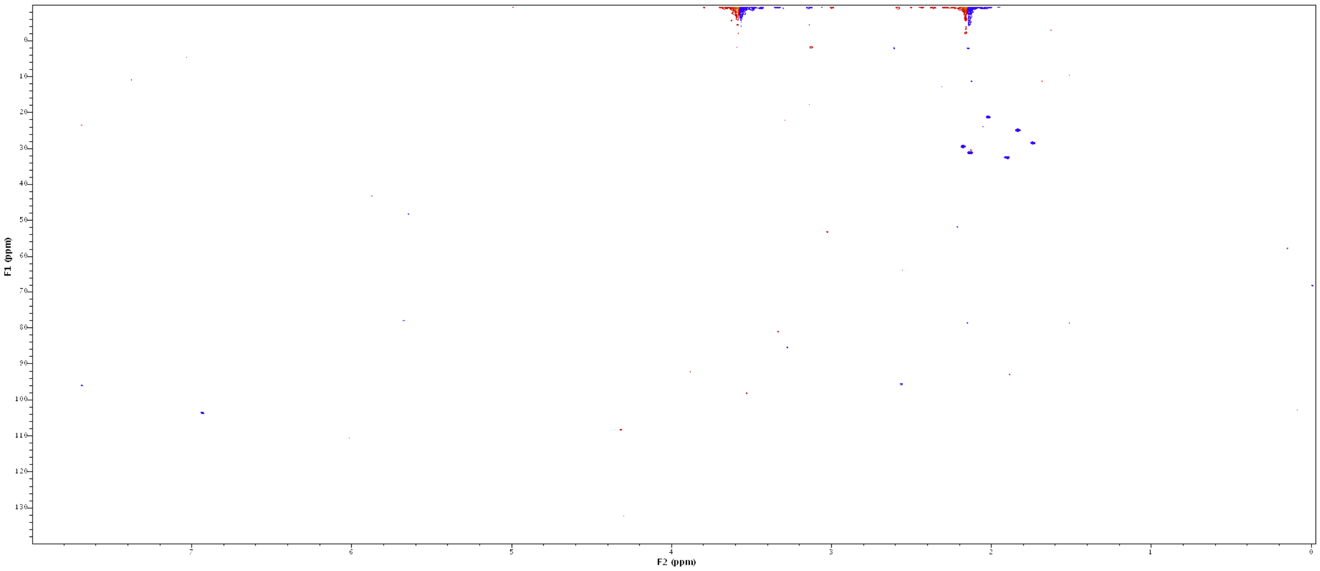

Figure S63. HSQCAD NMR spectrum (500 MHz, 75% CH_3CN/D_2O) of compound eluting at 14.53 min (**5**) (*H. pseudospicata*).

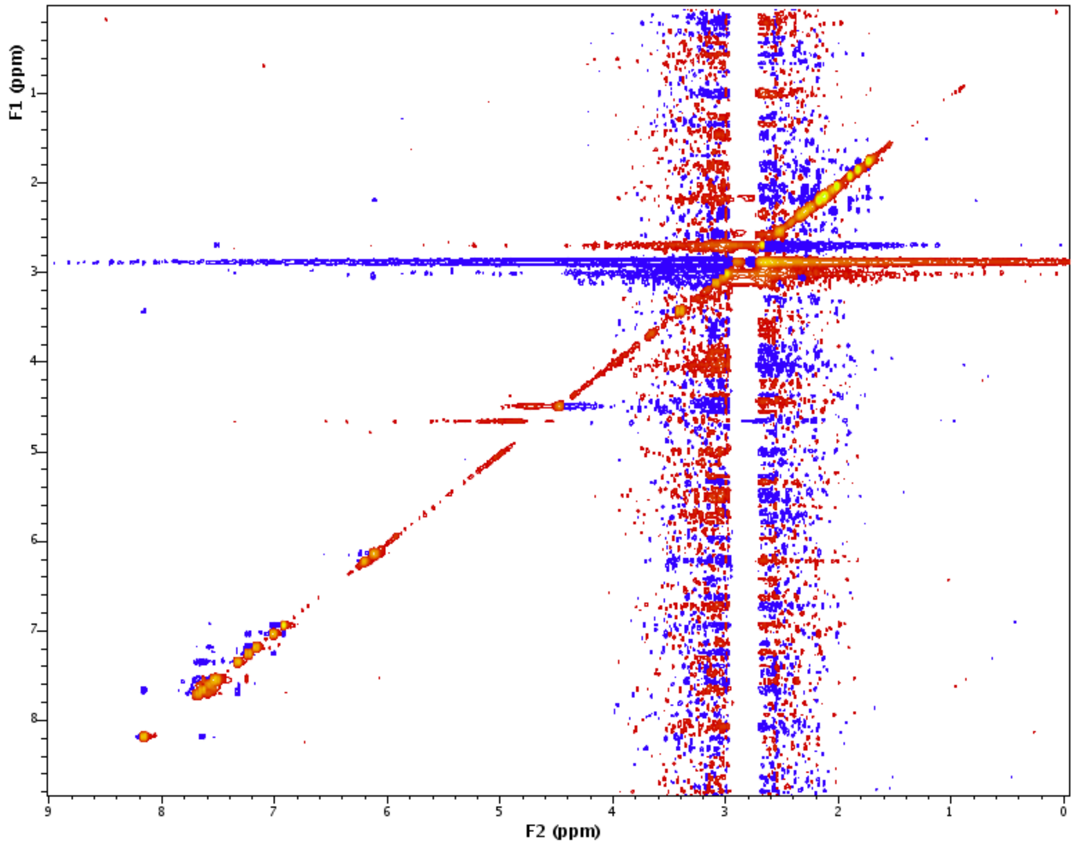

Figure S64. ROESYAD NMR spectrum (500 MHz, 75% CH_3CN/D_2O) of compound eluting at 14.53 min (**5**) (*H. pseudospicata*).

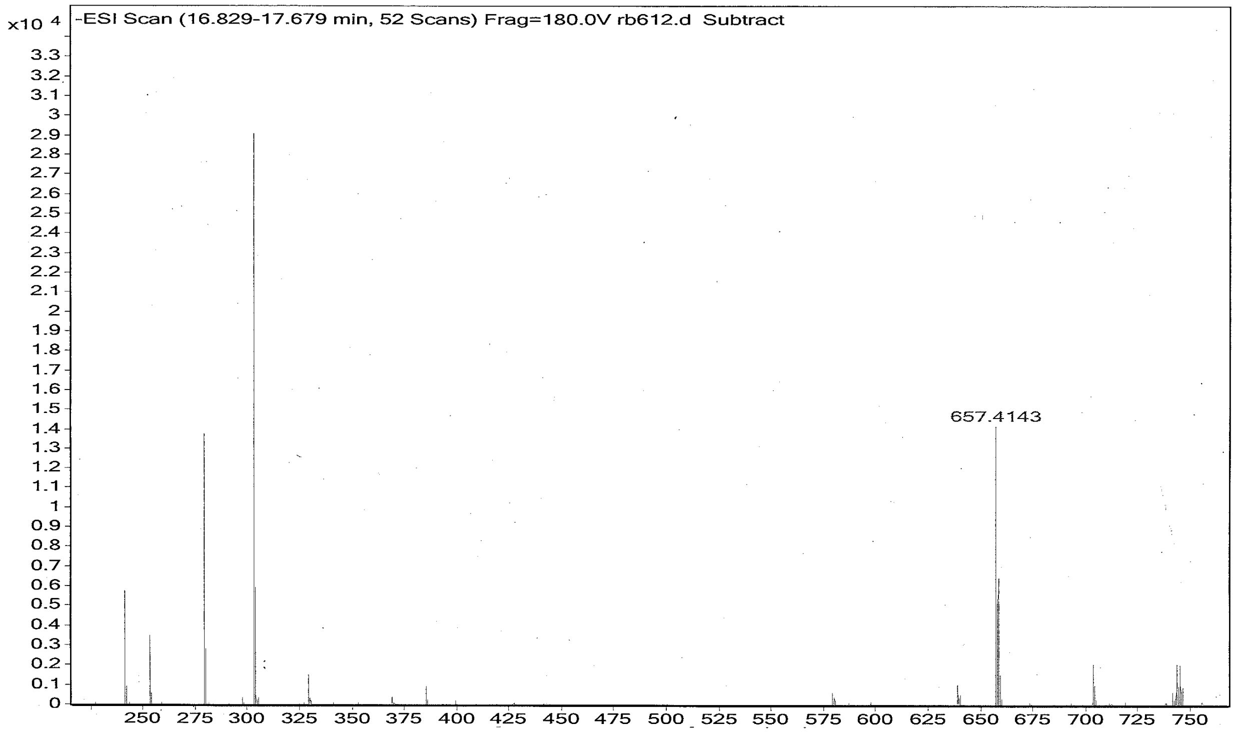

Figure S65. High resolution negative ESI-MS of compound eluting at 14.53 min (**5**) from HPLC-MS (*H. pseudospicata*).

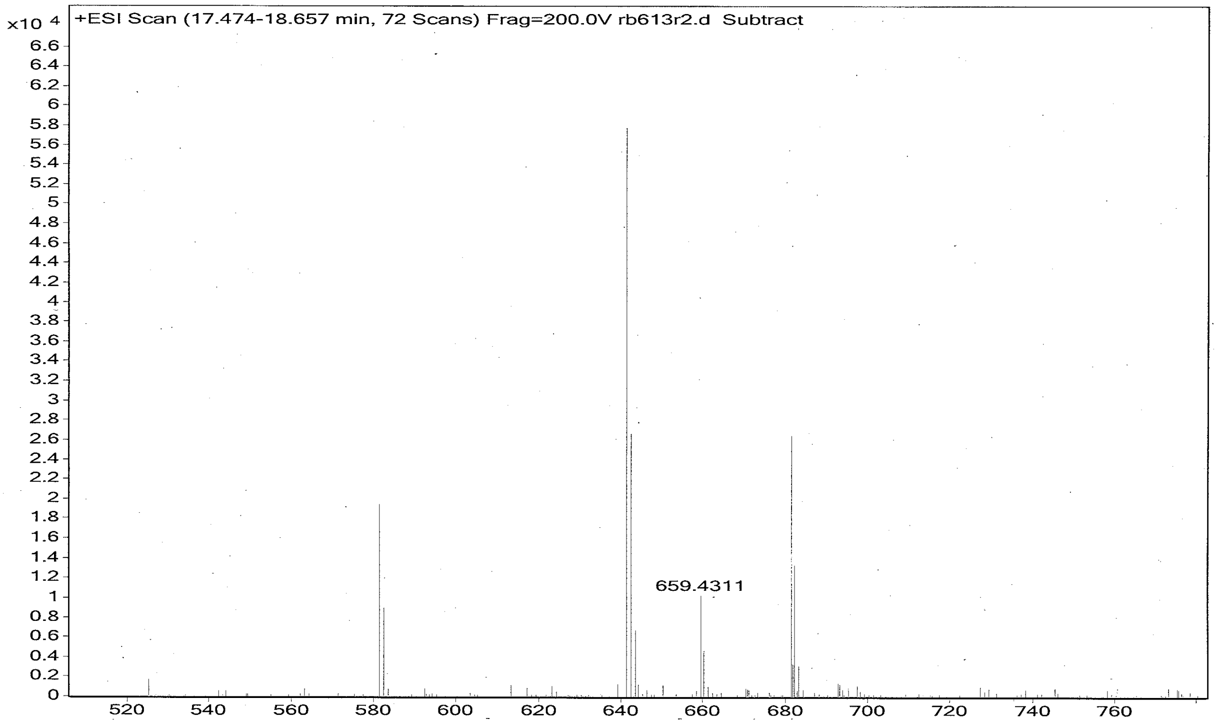

Figure S66. High resolution positive ESI-MS of compound eluting at 14.53 min (**5**) from HPLC-MS (*H. pseudospicata*).

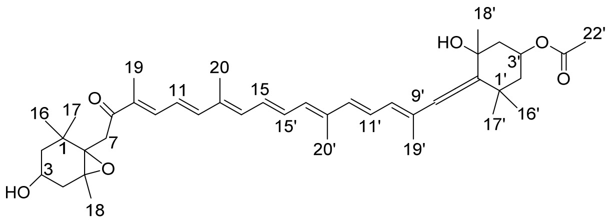

Position	δ_C[a], mult.	δ_H (*J* in Hz)	gCOSY	Roesyad
1	ND			
2a	ND	SS		
2b		SS		
3	ND	4.46, m		
4a	ND	SS		
4b		SS		
5	ND			
6	ND			
7a	ND	3.40, d (18.5)		
7b		SS		
8	ND			
9	ND			
10	ND	8.16, d (10.5)	11	7a, 12

Figure S67. *Cont.*

11	ND	7.54, m	10, 12	
12	ND	7.65, m		10, 14
13	ND			
14	ND	7.33, d (10.5)	15	12, 15′
15	ND	7.58, m	14	14′
16	24.7, CH_3	1.82, s		
17	28.2, CH_3	1.73, s		
18	21.0, CH_3	2.01, s		
19	ND	SS		
20	ND	SS		
1′	ND			
2a′	ND	2.29, m	2b′	16′
2b′		2.04[b]		
3′	ND	6.12, m	2a′, 4b′	
4a′	ND	2.51, m	4b′	
4b′		3.02[b]		
5′	ND			
6′	ND			
7′	ND			
8′	103.4, CH	6.92, s		10′
9′	ND			
10′	ND	7.01, d (11.5)	11′, 19′	8′, 12′
11′	ND	7.53, m	10′, 12′	19′
12′	ND	7.23, d (15.0)		10′, 14′
13′	ND			
14′	ND	7.16, d (12.0)	15′	
15′	ND	7.69, m	14′	
16′	29.2, CH_3	2.16, s		
17′	32.3, CH_3	1.89, s		
18′	30.9, CH_3	2.12, s		
19′	ND	2.67[b]		
20′	ND	SS		
21′	ND			
22′	ND	SS		
3-OH		ND		
5′-OH		ND		

Referenced to 75% CH_3CN/D_2O; [a] Carbon assignments based on HSQCAD NMR experiment; [b] Proton assignment based on gCOSY experiment; ND Not Detected; SS Signal suppressed.

Figure S67. NMR data for compound eluting at 14.53 min (**5**) (*H. pseudospicata*).

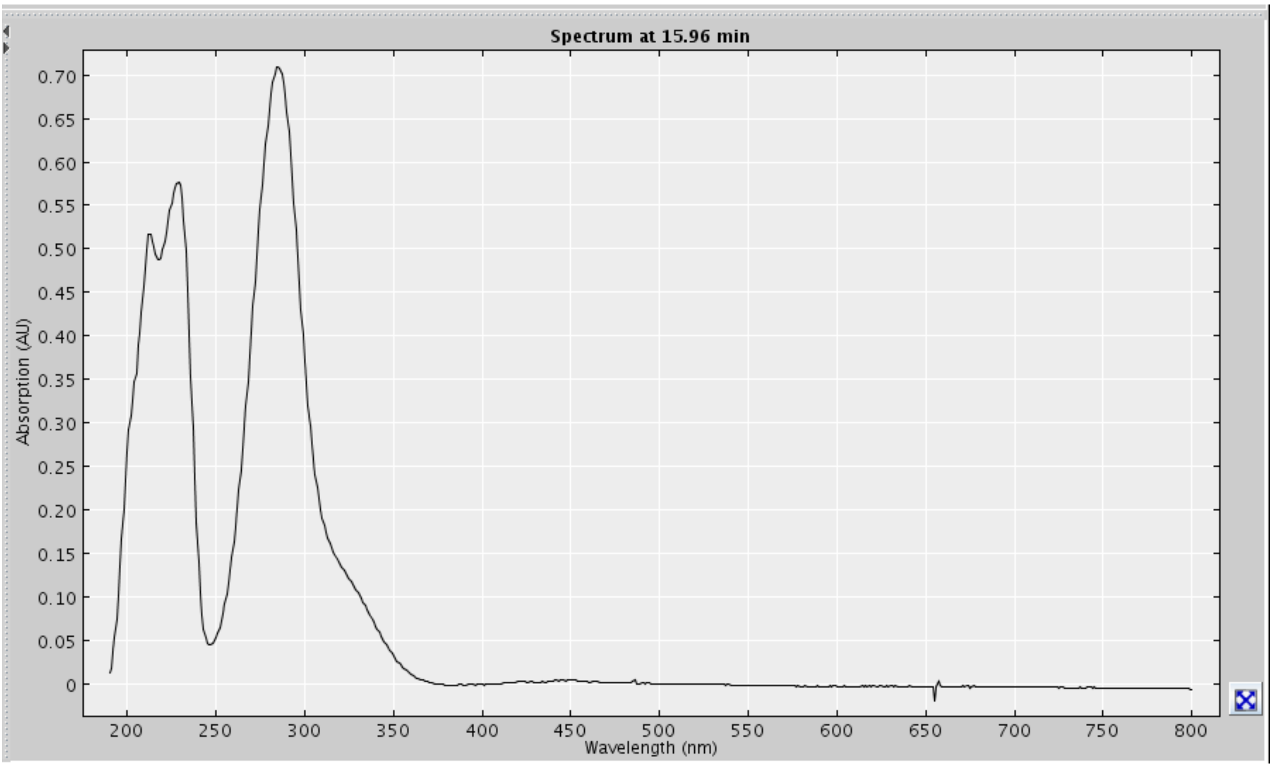

Figure S68. Extracted UV profile of compound eluting at 15.50 min (**20**) from HPLC-NMR (*S. cf. fallax*).

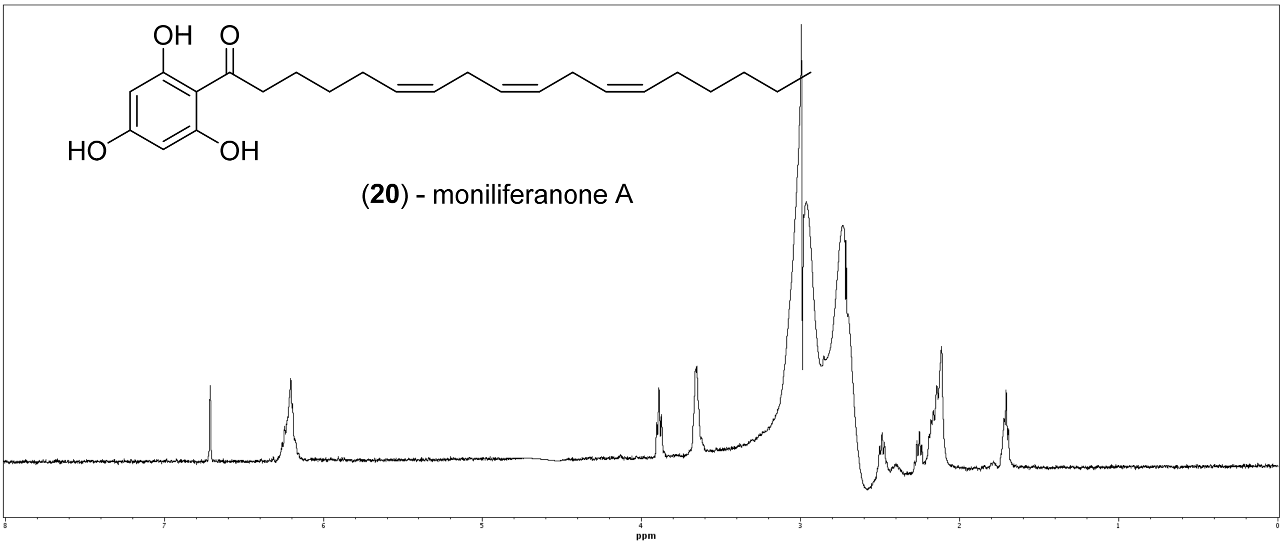

Figure S69. WET1D Proton NMR spectrum (500 MHz, 75% CH_3CN/D_2O) of compound eluting at 15.50 min (**20**) (*S. cf. fallax*).

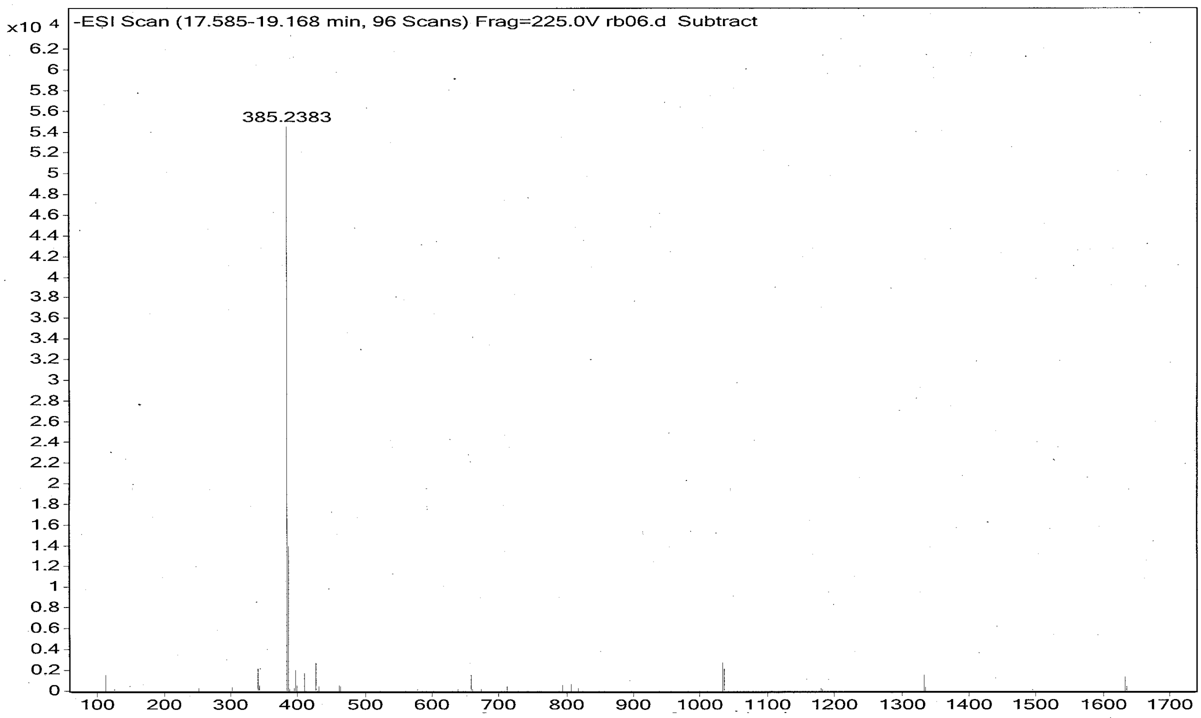

Figure S70. High resolution negative ESI-MS of compound eluting at 15.50 min (**20**) from HPLC-MS (*S. cf. fallax*).

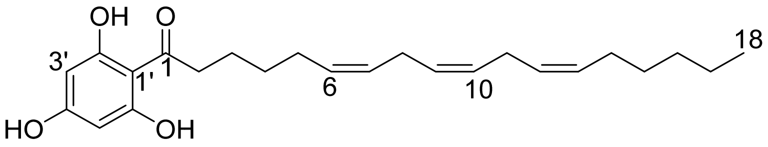

(**20**) - moniliferanone A

Position	δ_H (*J* in Hz)
1	
2	3.88, t (7.5)
3	2.48, p (7.5)
4	2.25, p (7.5)
5	SS
6	6.20, m
7	6.20, m
8	3.65, m
9	6.20, m
10	6.20, m
11	3.65, m
12	6.20, m
13	6.20, m
14	SS
15	2.11–2.16, m
16	2.11–2.16, m
17	2.11–2.16, m
18	1.71, t (7.0)
1′	
2′	
3′	6.71, s
4′	
5′	6.71, s
6′	
1′-OH	ND
4′-OH	ND
6′-OH	ND

Referenced to 75% CH_3CN/D_2O; SS Signal suppressed; ND Not Detected.

Figure S71. NMR data for compound eluting at 15.50 min (**20**) (*S. cf. fallax*).

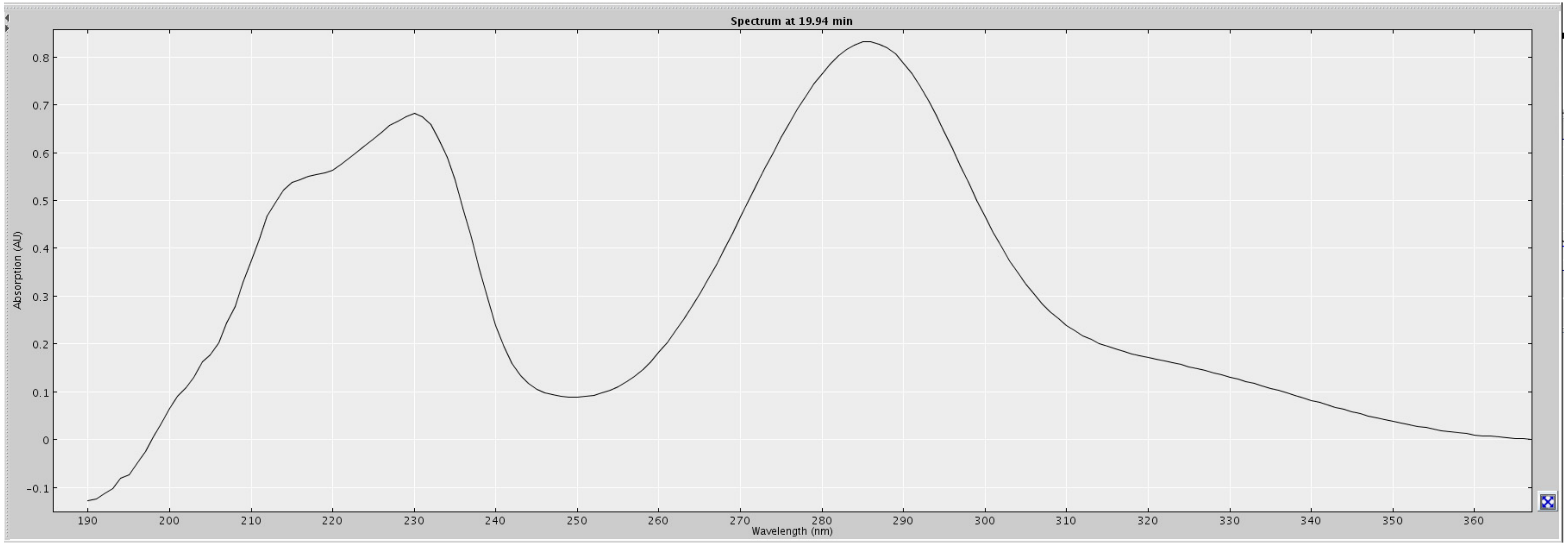

Figure S72. Extracted UV profile of compound eluting at 20.15 min (**21**) from HPLC-NMR (*C. retroflexa*).

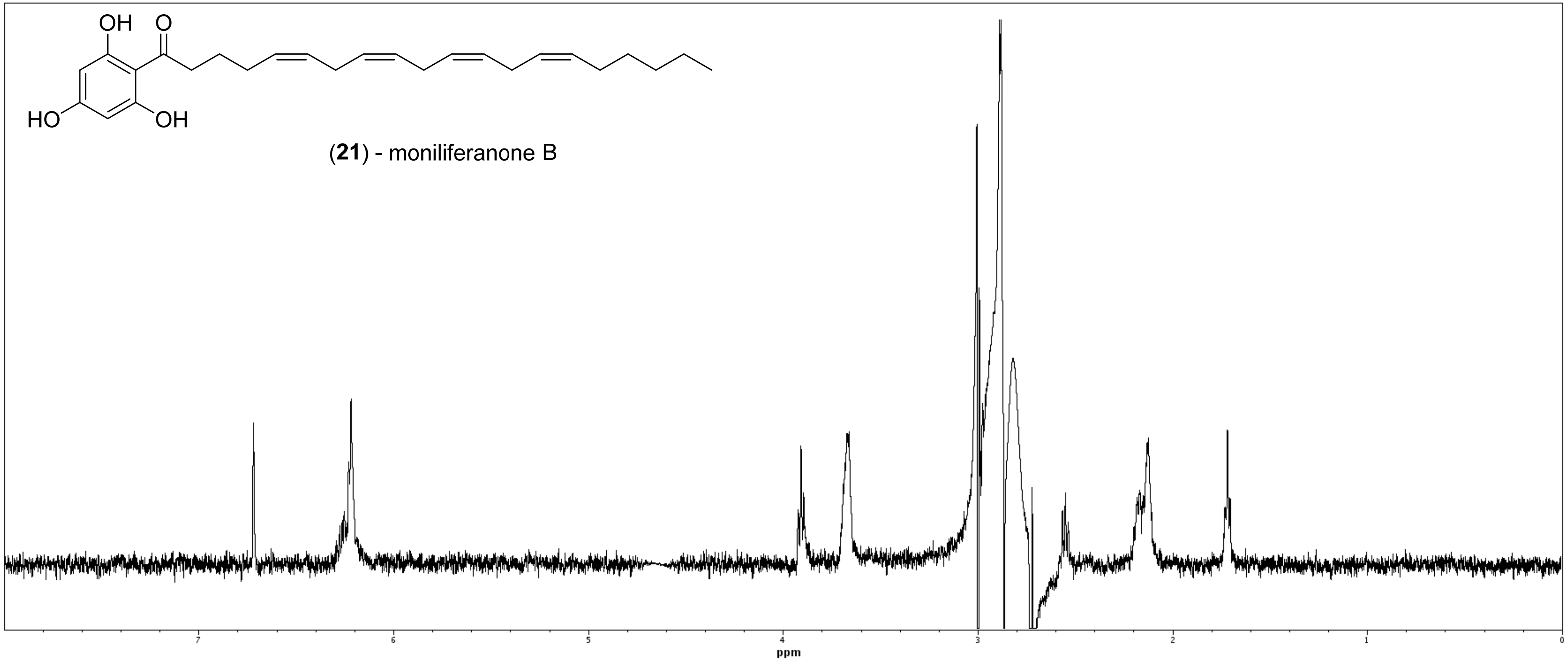

Figure S73. WET1D Proton NMR spectrum (500 MHz, 75% CH_3CN/D_2O) of compound eluting at 20.15 min (**21**) (*C. retroflexa*).

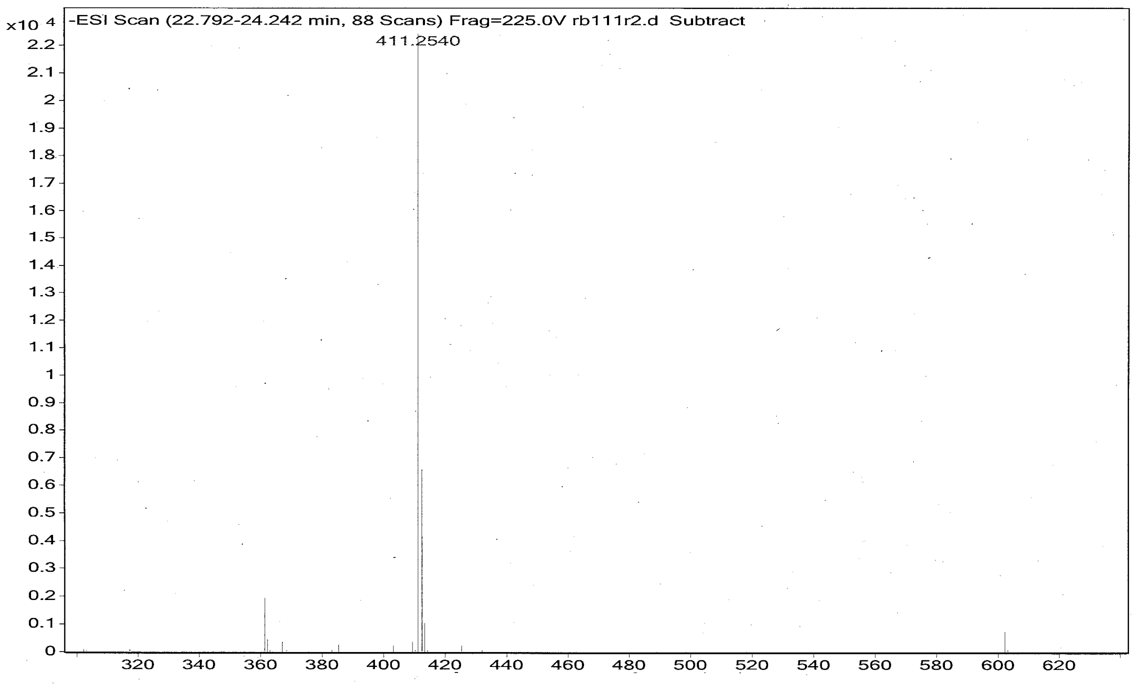

Figure S74. High resolution negative ESI-MS of compound eluting at 20.15 min (**21**) from HPLC-MS (*C. retroflexa*).

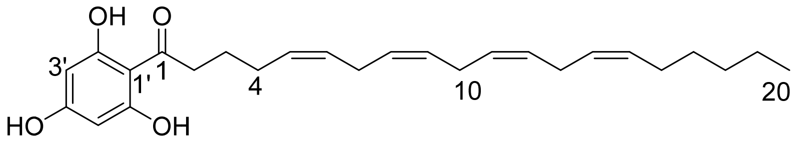

(**21**) - moniliferanone B

Position	δ_H (*J* in Hz)
1	
2	3.91, t (7.0)
3	2.55 , m
4	SS
5	6.16–6.24, m
6	6.16–6.24, m
7	3.66, m
8	6.16–6.24, m
9	6.16–6.24, m
10	3.66, m
11	6.16–6.24, m
12	6.16–6.24, m
13	3.66, m
14	6.16–6.24, m
15	6.16–6.24, m
16	SS
17	SS
18	SS
19	SS
20	1.72, t (7.0)
1′	
2′	
3′	6.72, s
4′	
5′	6.72, s
6′	
2′-OH	ND
4′-OH	ND
6′-OH	ND

Referenced to D_2O (δ_H 4.64 ppm); SS Signal suppressed; ND Not Detected.

Figure S75. NMR data for compound eluting at 20.15 min (**21**) (*C. retroflexa*).

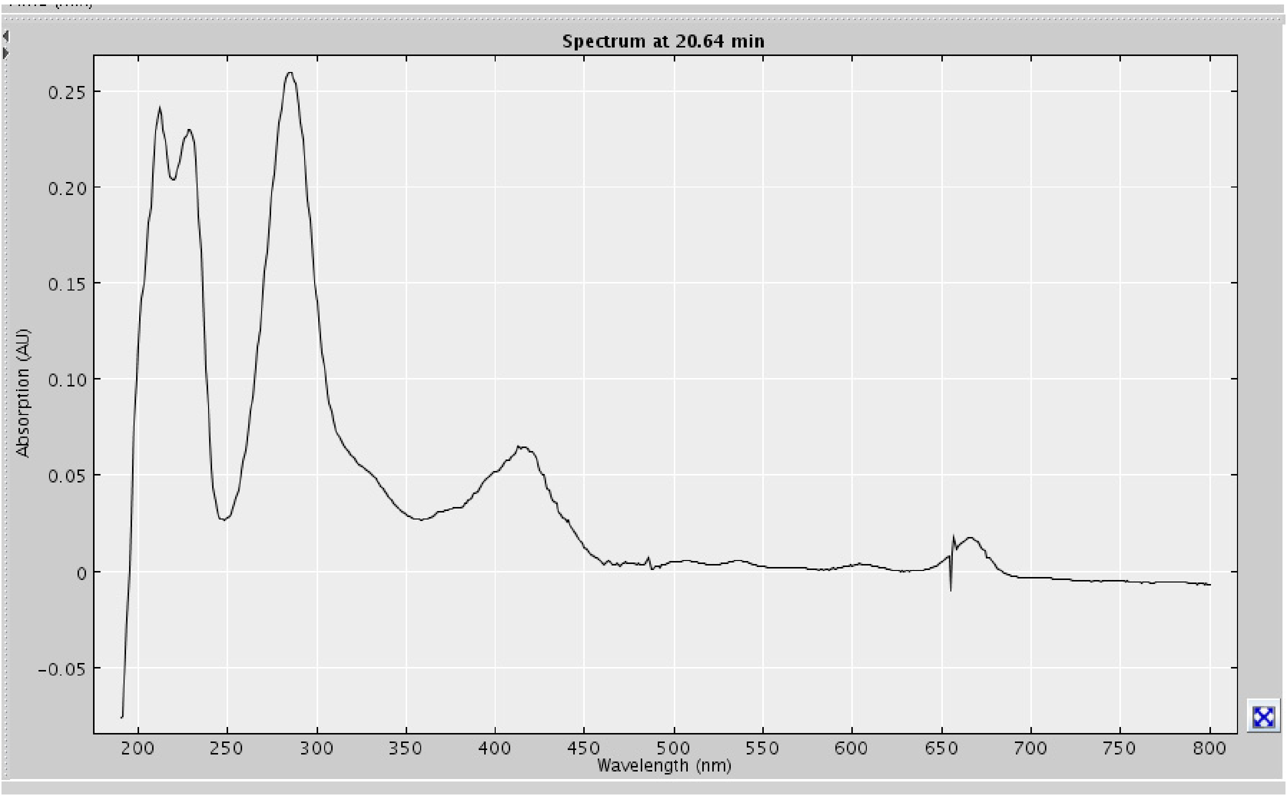

Figure S76. Extracted UV profile of compound eluting at 21.62 min (**14**) from HPLC-NMR (*S. cf. fallax*).

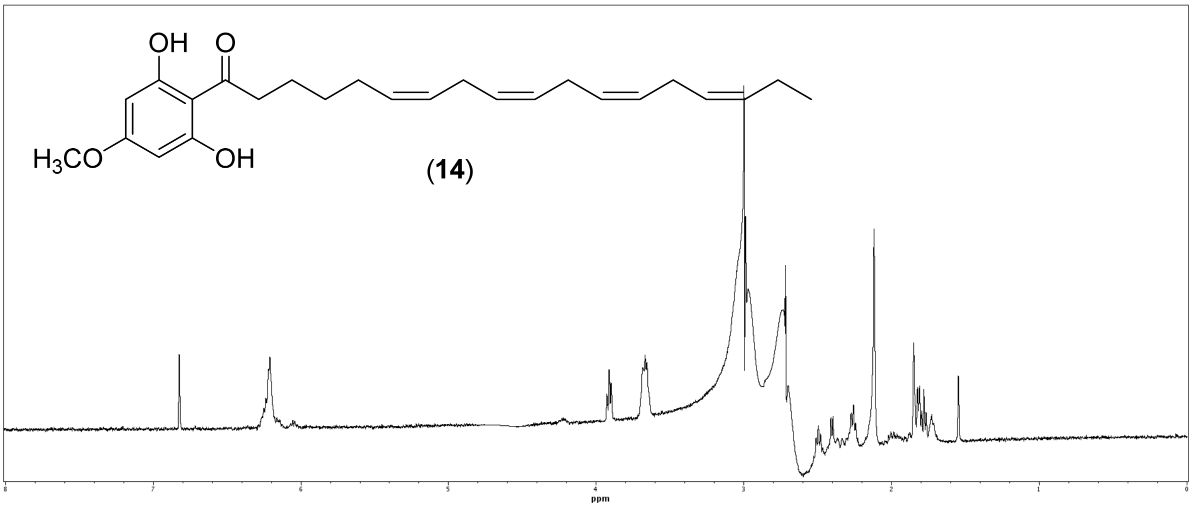

Figure S77. WET1D Proton NMR spectrum (500 MHz, 75% CH_3CN/D_2O) of compound eluting at 21.62 min (**14**) (*S. cf. fallax*).

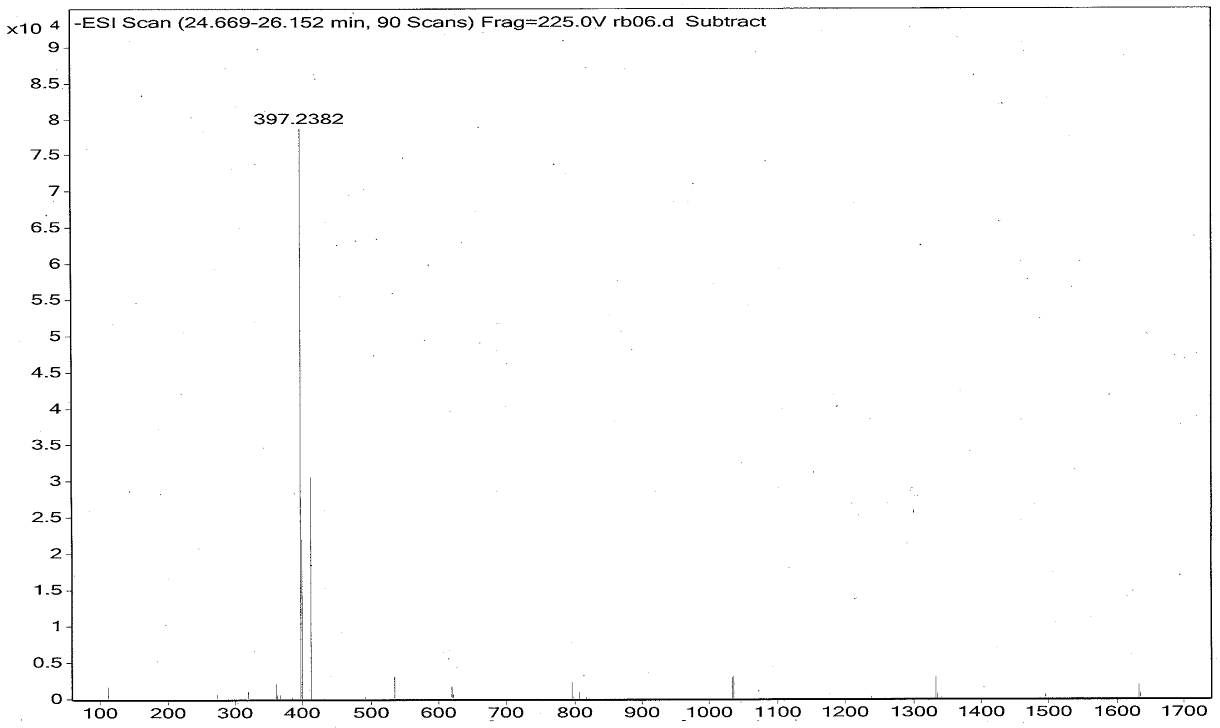

Figure S78. High resolution negative ESI-MS of compound eluting at 21.62 min (**14**) from HPLC-MS (*S. cf. fallax*).

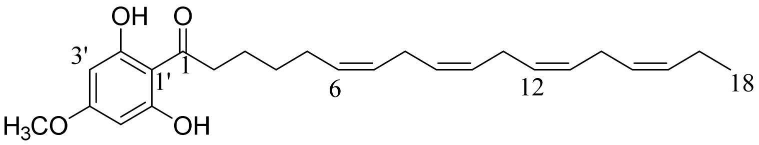

(**14**)	
**Position**	**δ_H (*J* in Hz)**
1	
2	3.91, t (8.0)
3	2.49, m
4	2.25, m
5	2.40, m *
6	6.21, m
7	6.21, m
8	3.66, m
9	6.21, m
10	6.21, m
11	3.66, m
12	6.21, m
13	6.21, m
14	3.66, m
15	6.21, m
16	6.21, m
17	SS *
18	1.78, t (7.5)
1′	
2′	
3′	6.81, s
4′	
5′	6.81, s
6′	
1′-OH	ND
4′-OCH_3	SS
6′-OH	ND

Referenced to 75% CH_3CN/D_2O; * signals interchangeable; SS Signal suppressed; ND Not Detected.

Figure S79. NMR data for compound eluting at 21.62 min (**14**) (*S. cf. fallax*).

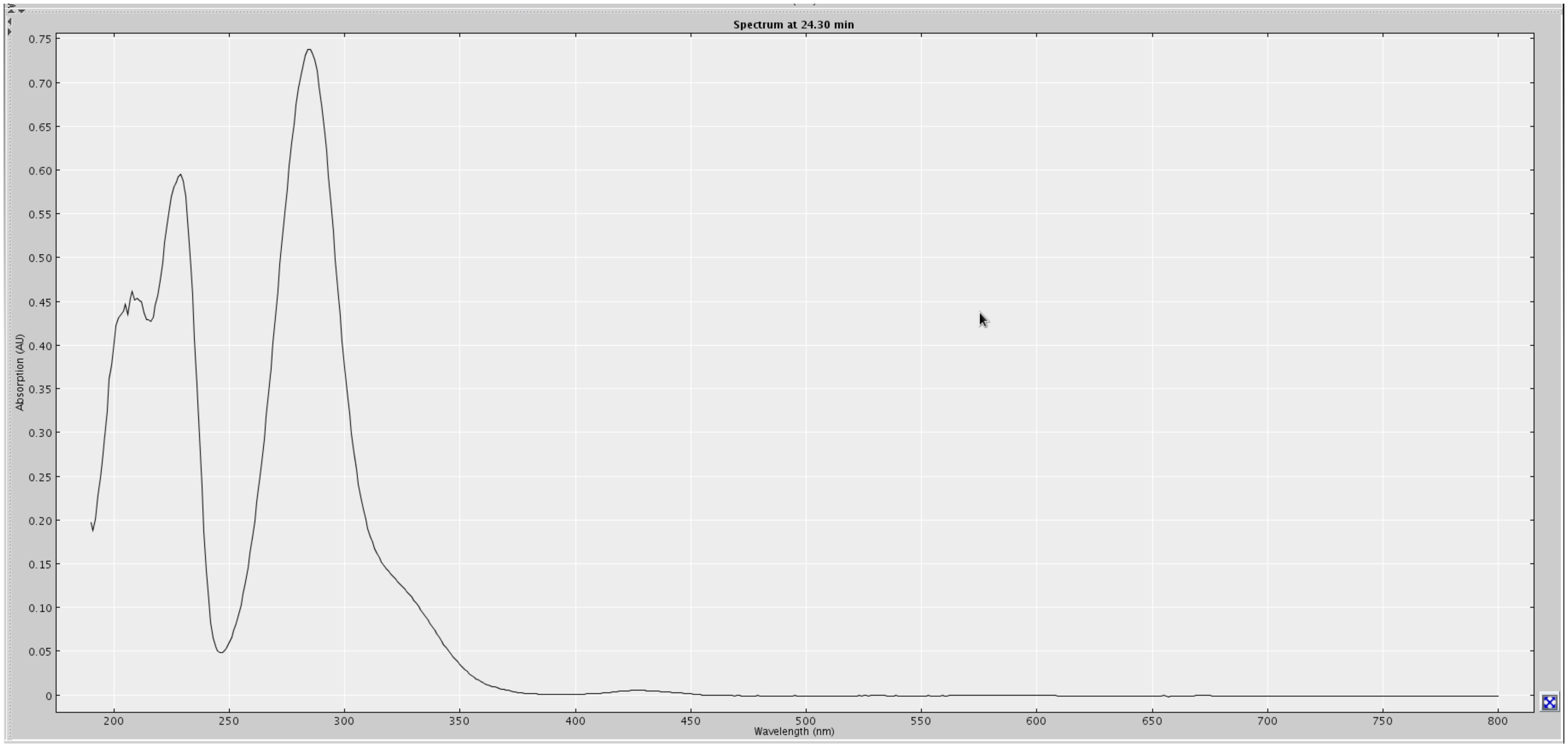

Figure S80. Extracted UV profile of compound eluting at 22.96 min (**18**) from HPLC-NMR (*C. subfarcinata*).

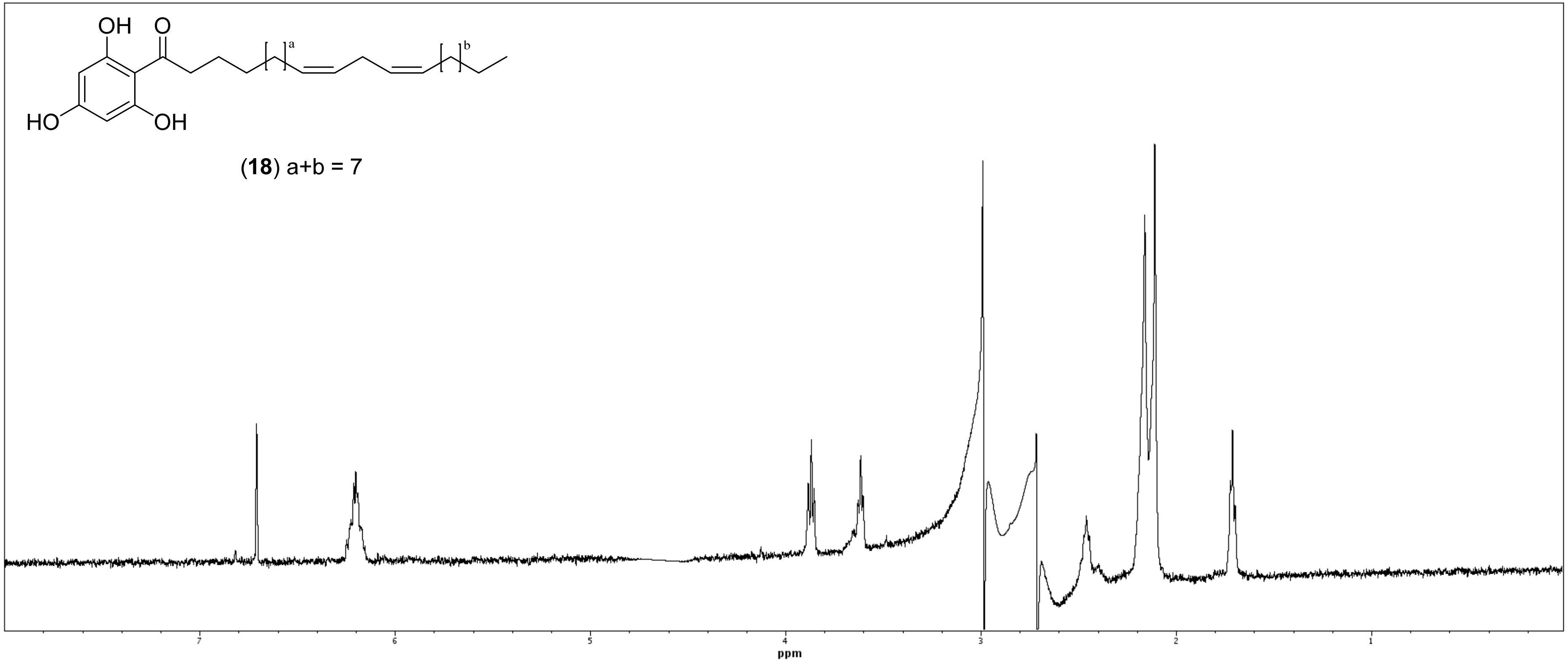

Figure S81. WET1D Proton NMR spectrum (500 MHz, 75% CH_3CN/D_2O) of compound eluting at 22.96 min (**18**) (*C. subfarcinata*).

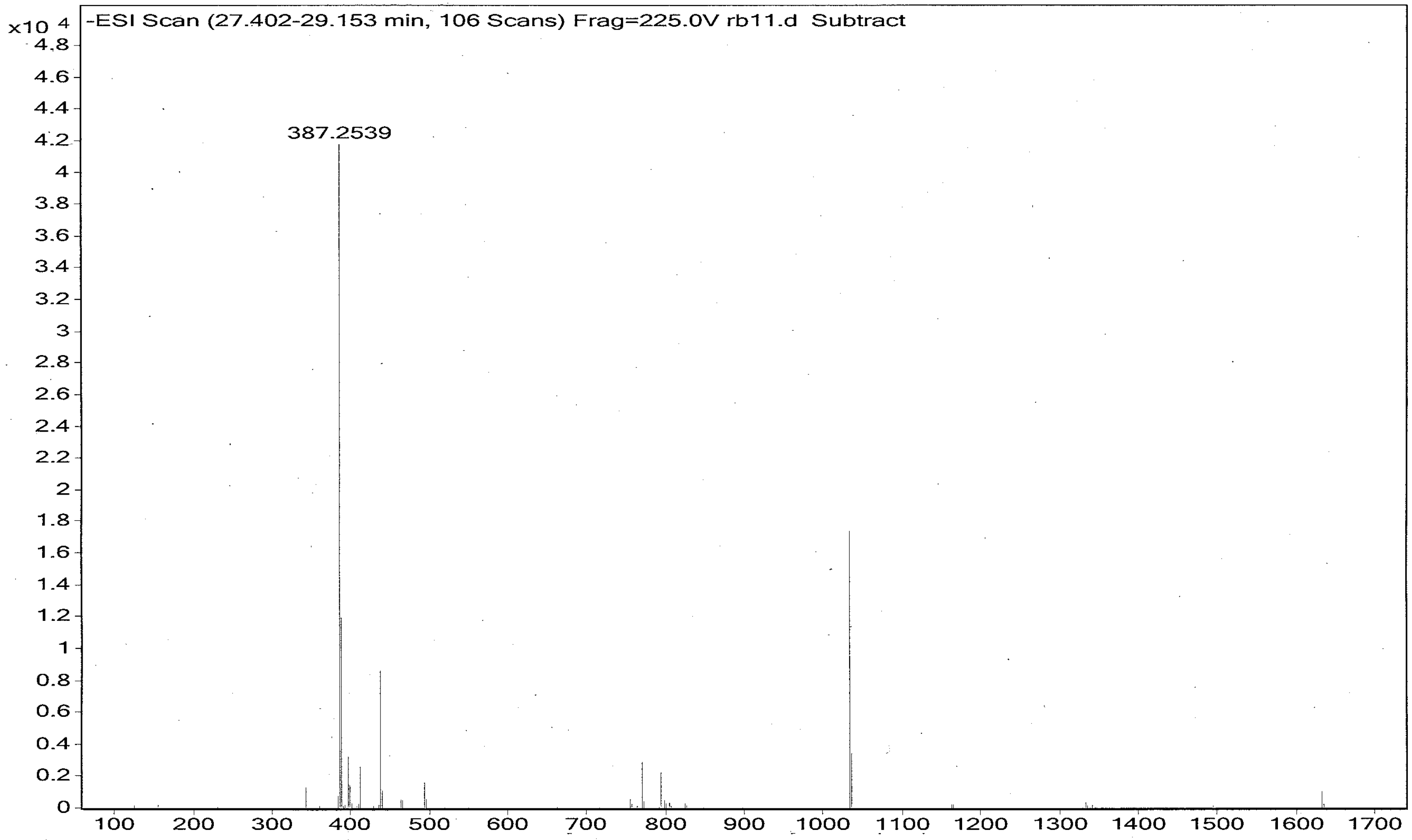

Figure S82. High resolution negative ESI-MS of compound eluting at 22.96 min (**18**) from HPLC-MS (*C. subfarcinata*).

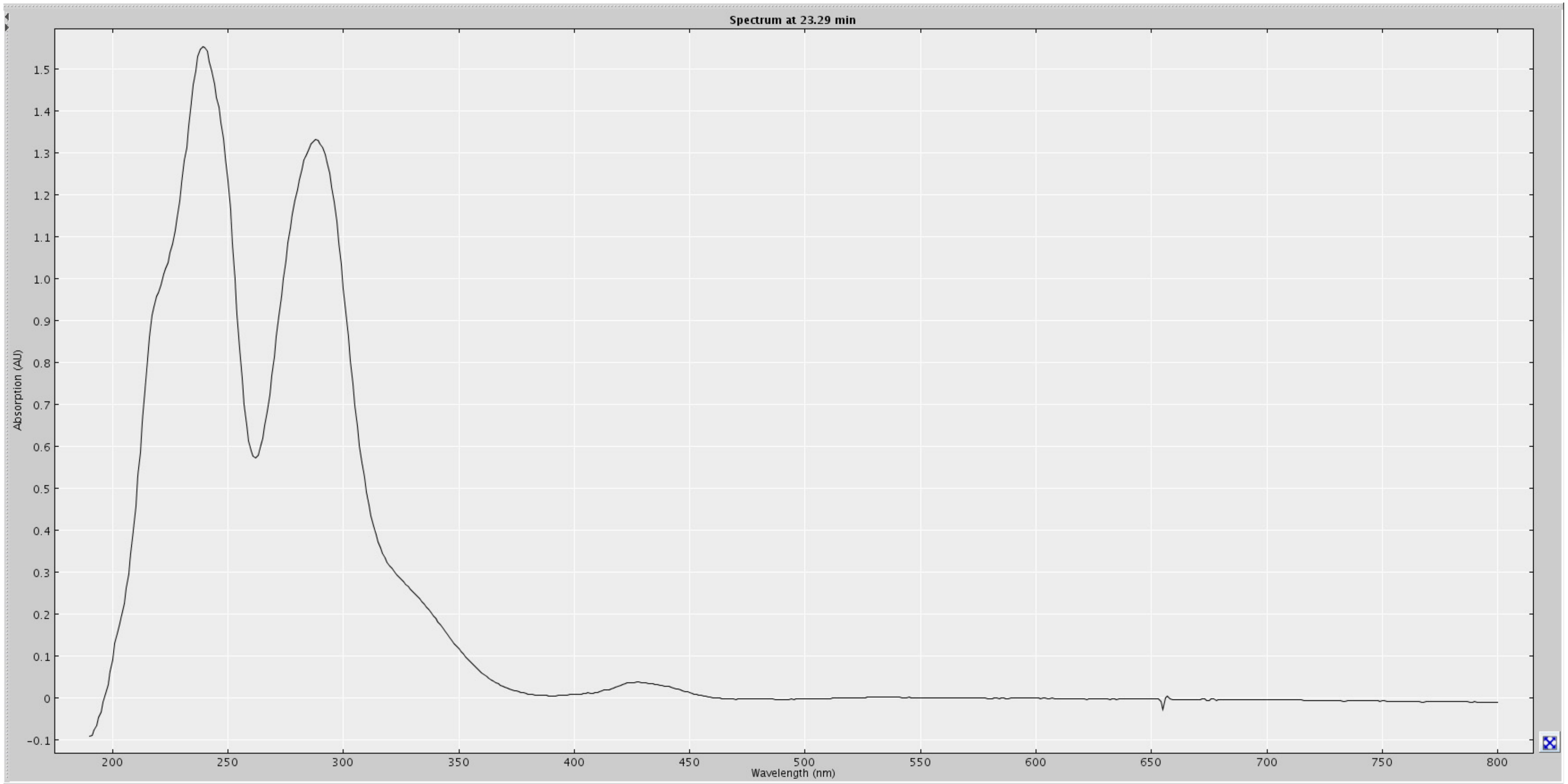

Figure S83. Extracted UV profile of compound eluting at 23.16 min from HPLC-NMR (*C. retroflexa*).

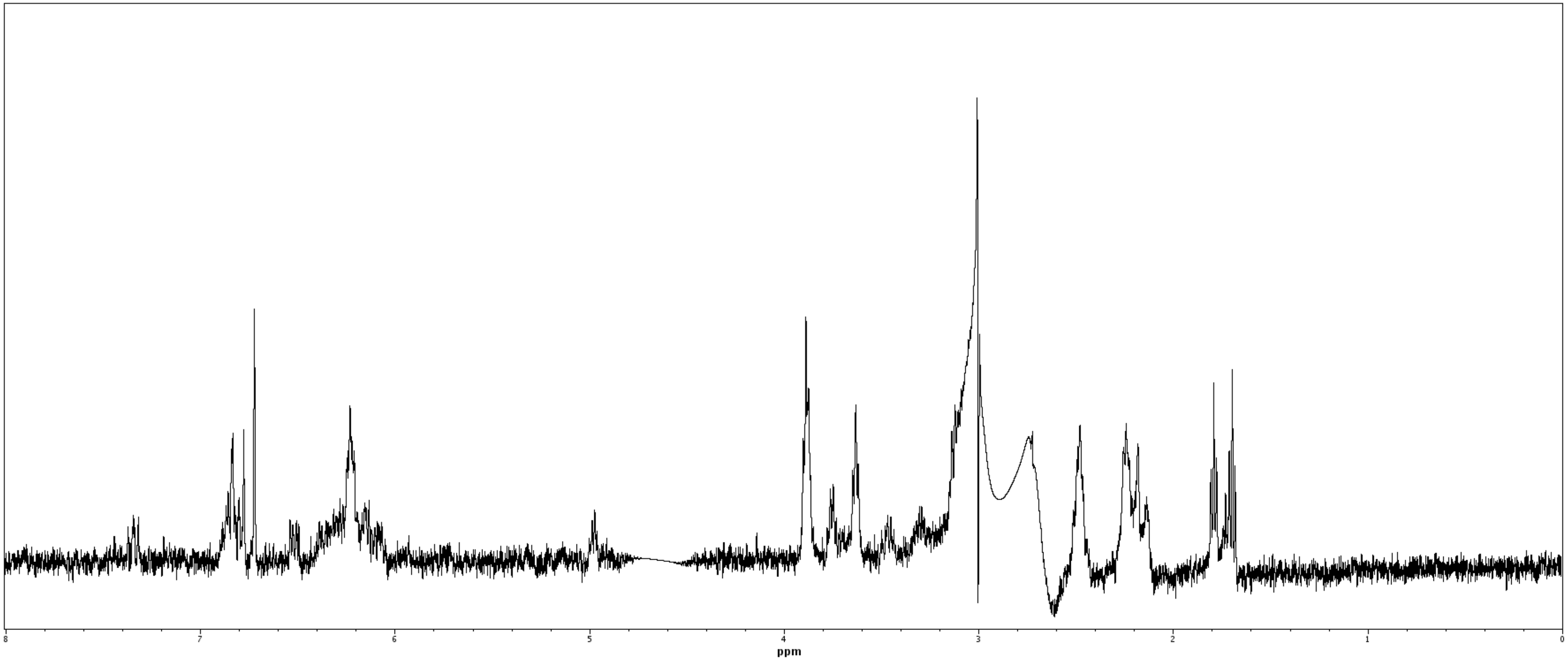

Figure S84. WET1D Proton NMR spectrum (500 MHz, 75% CH_3CN/D_2O) of compound eluting at 23.16 min (*C. retroflexa*).

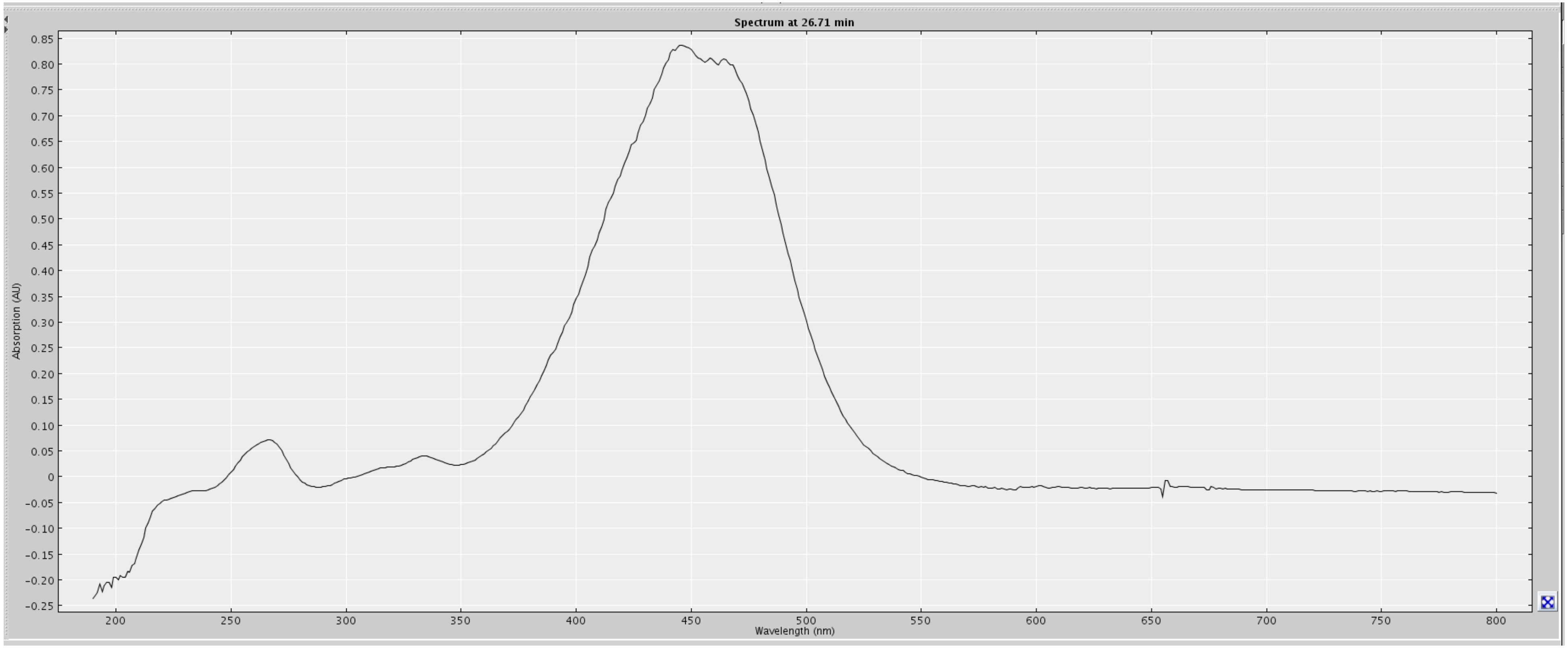

Figure S85. Extracted UV profile of compound eluting at 26.71 min from HPLC-NMR (*H. pseudospicata*).

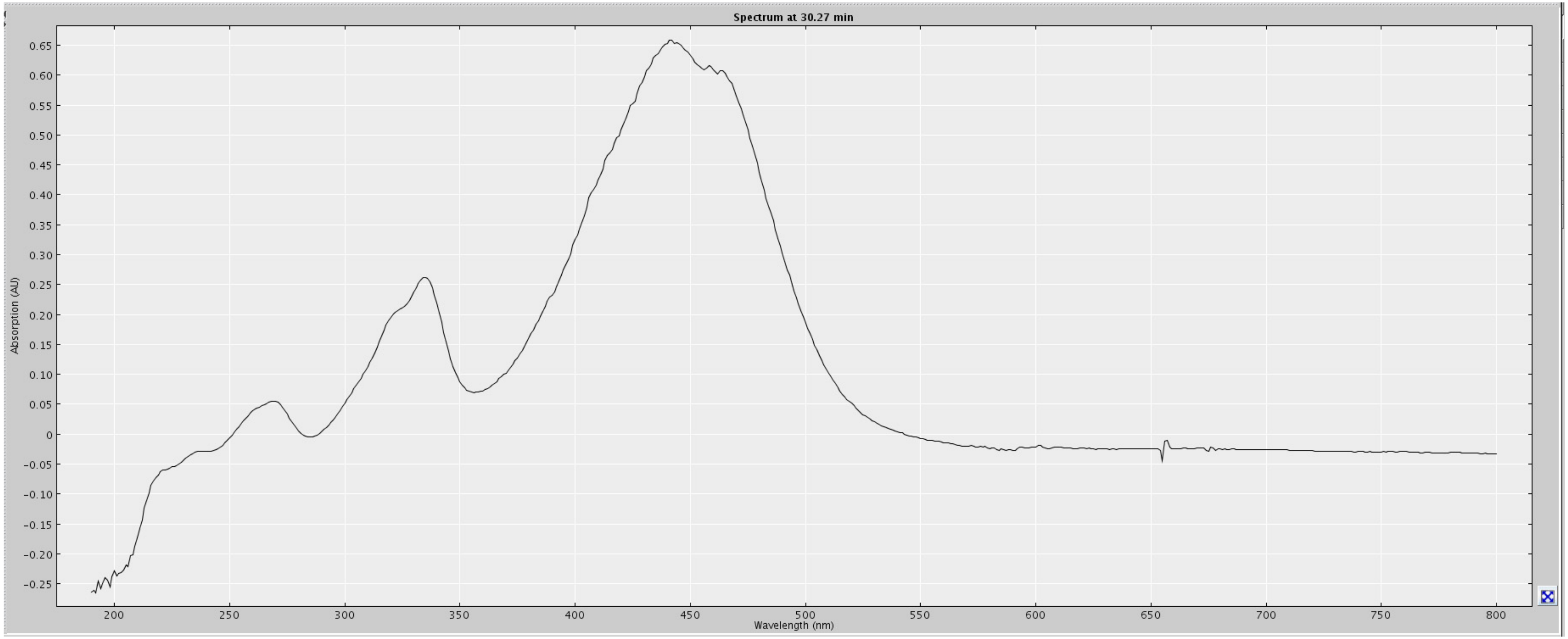

Figure S86. Extracted UV profile of compound eluting at 30.27 min from HPLC-NMR (*H. pseudospicata*).

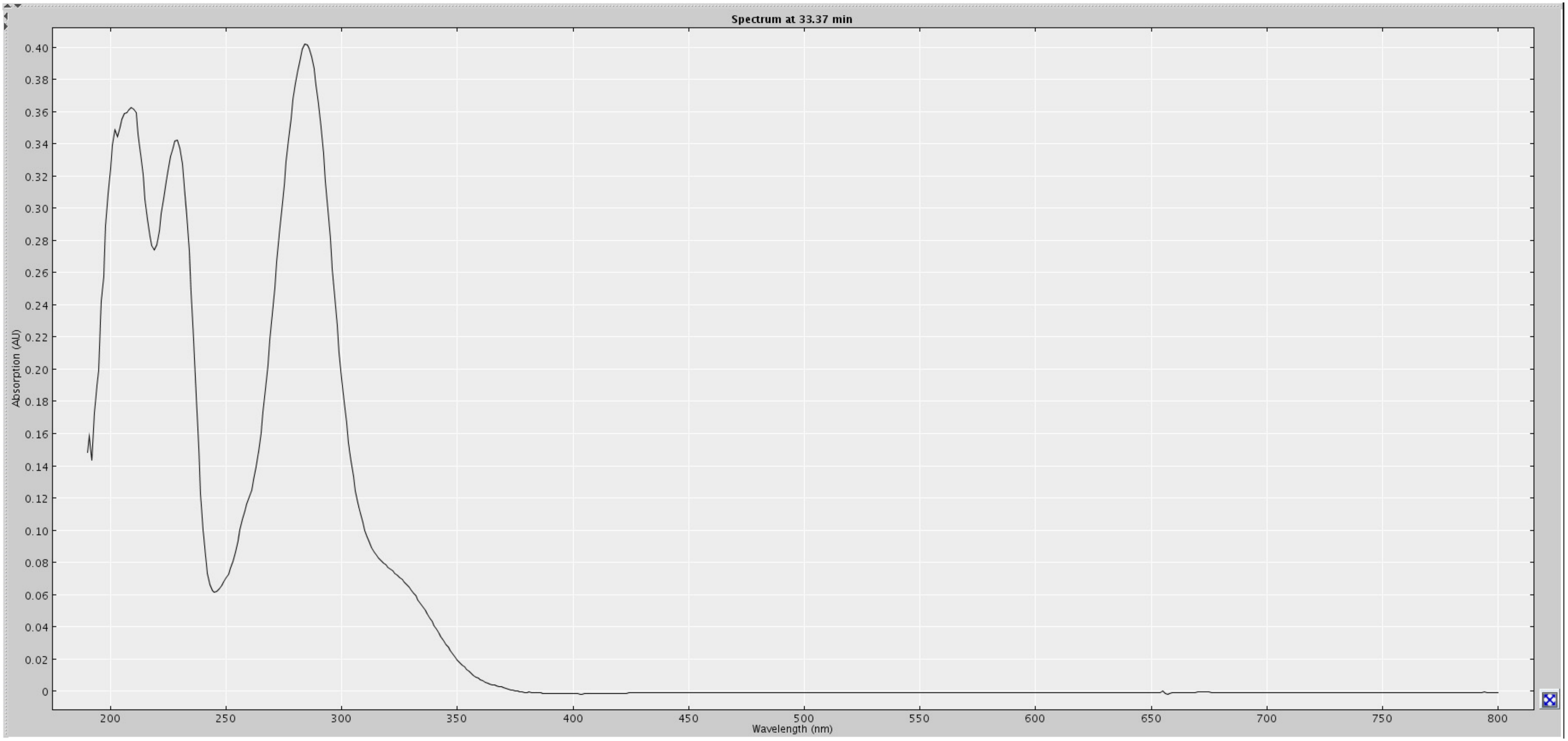

Figure S87. Extracted UV profile of compound eluting at 33.40 min (**19**) from HPLC-NMR (*C. subfarcinata*).

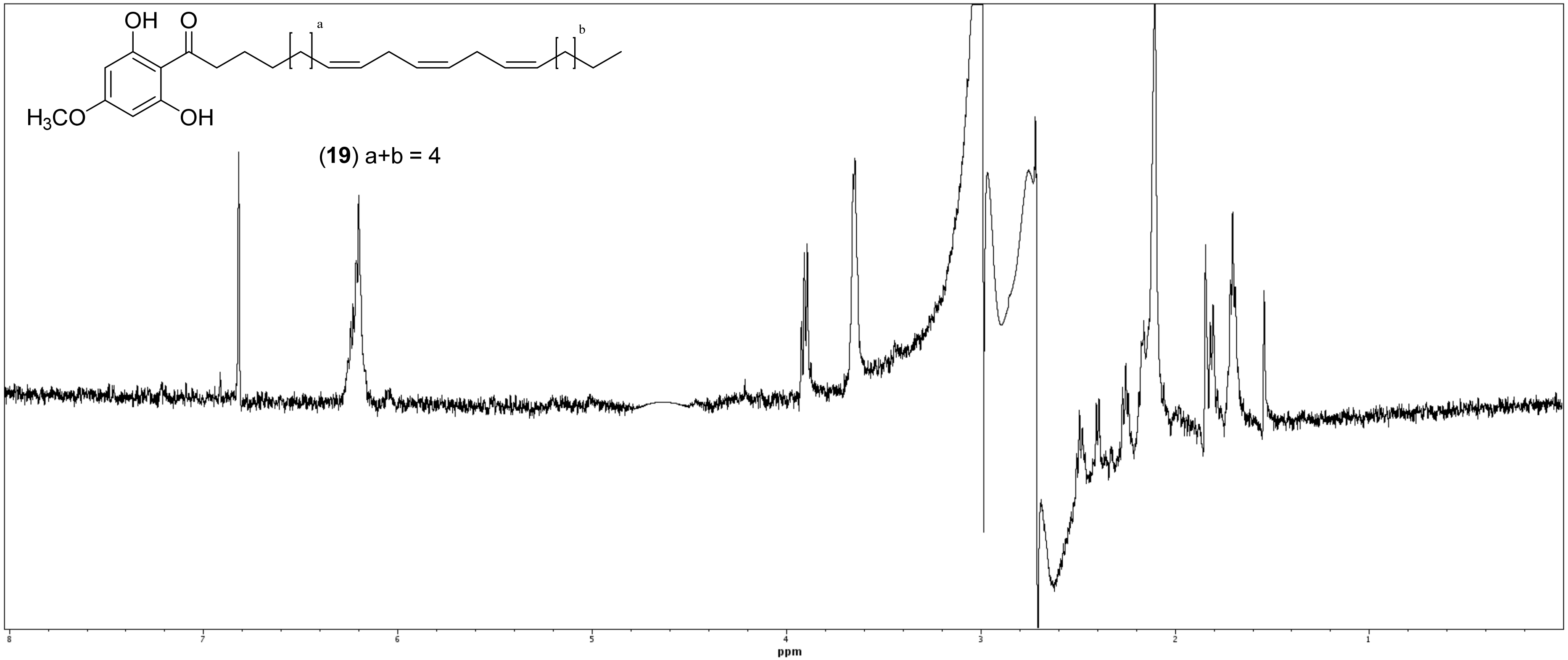

Figure S88. WET1D Proton NMR spectrum (500 MHz, 75% CH_3CN/D_2O) of compound eluting at 33.40 min (**19**) (*C. subfarcinata*).

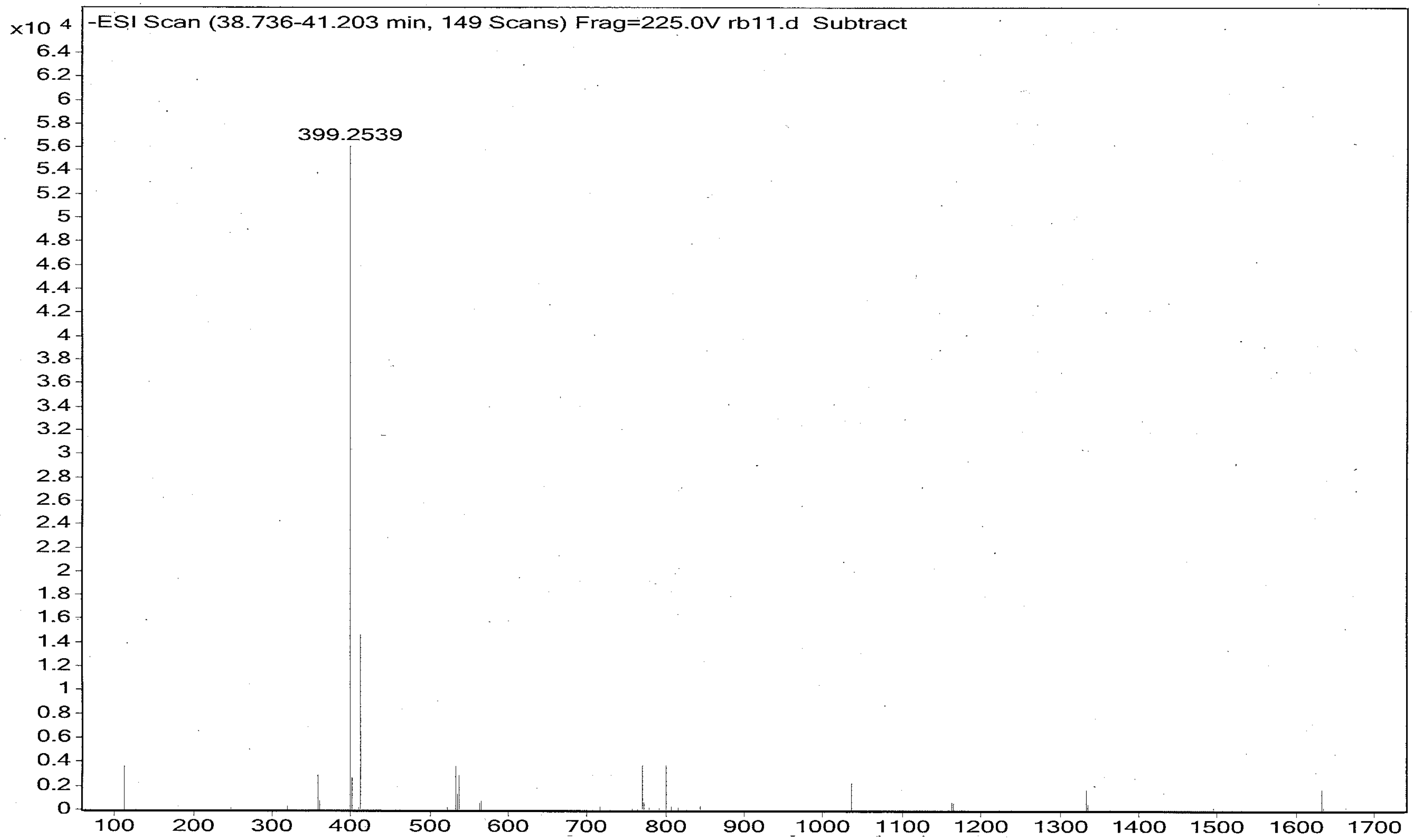

Figure S89. High resolution negative ESI-MS of compound eluting at 33.40 min (**19**) from HPLC-MS (*C. subfarcinata*).

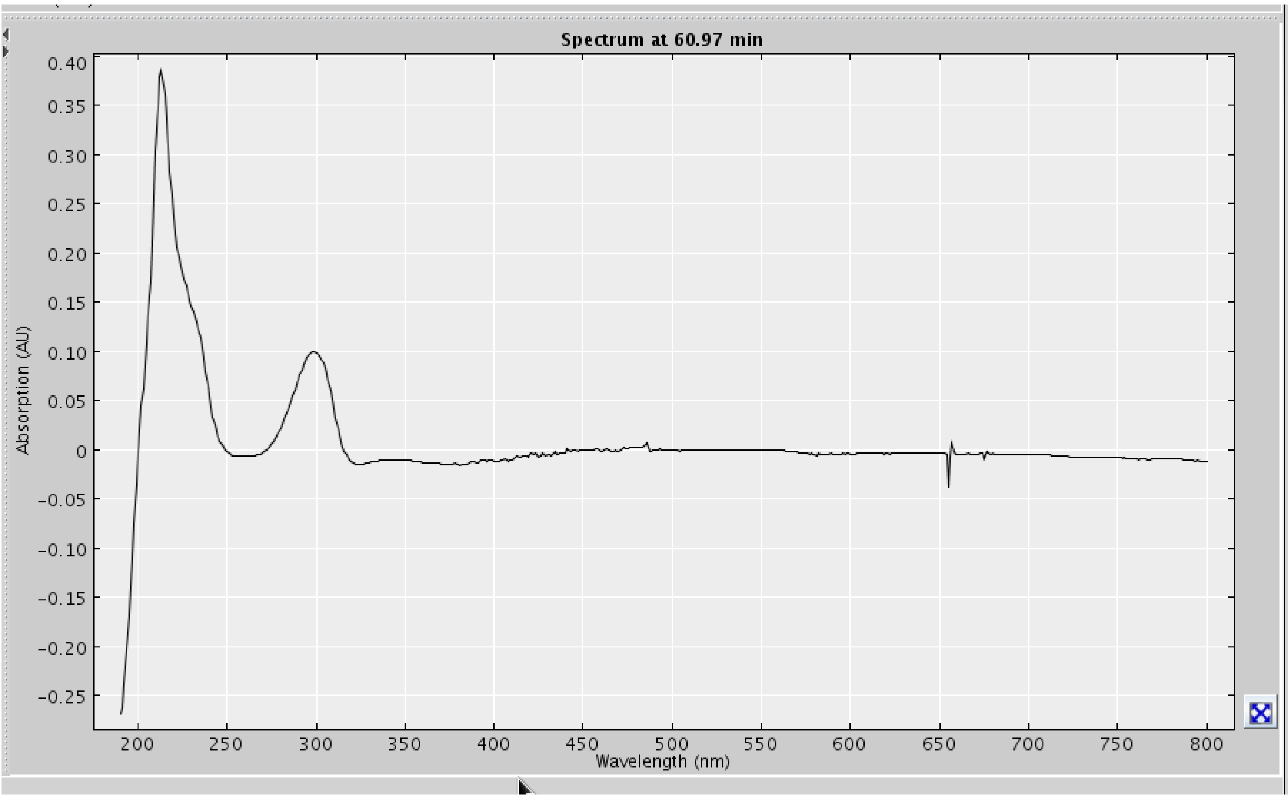

Figure S90. Extracted UV profile of compound eluting at 60.80 min (**15**) from HPLC-NMR (*S. cf. fallax*).

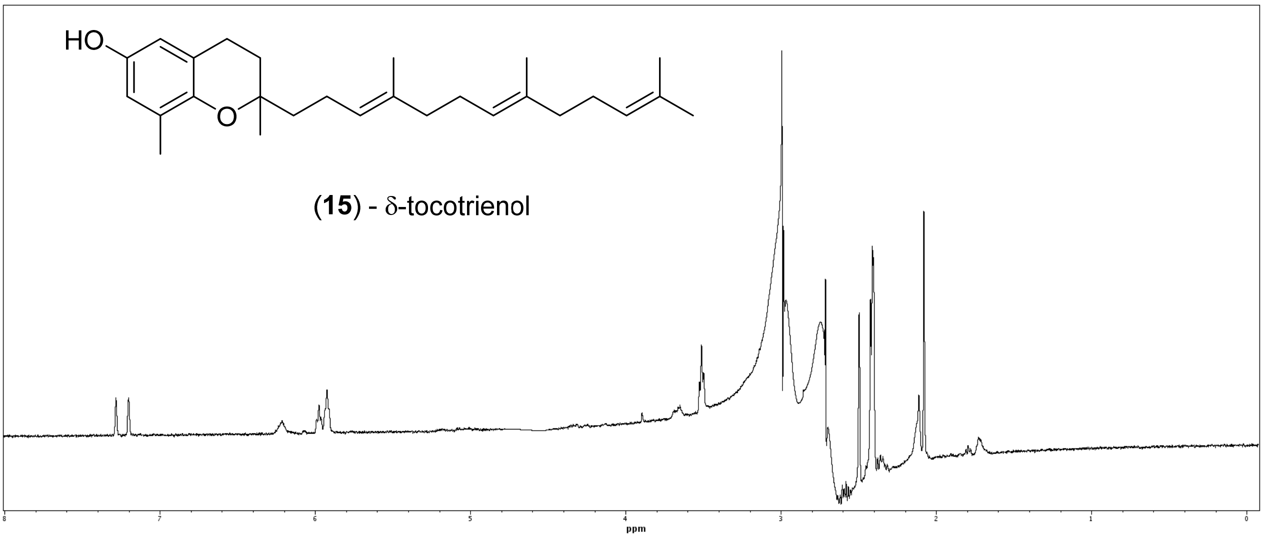

Figure S91. WET1D Proton NMR spectrum (500 MHz, 75% CH_3CN/D_2O) of compound eluting at 60.80 min (**15**) (*S. cf. fallax*).

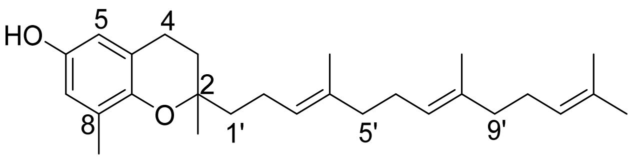

(**15**) - δ-tocotrienol	
**Position**	**δ_H (*J* in Hz)**
1	
2	
3	SS
4	3.51, t (7.0)
5	7.20, s
6	
7	7.28, s
8	
9	
10	
1′	SS
2′	SS
3′	5.97, t (7.0)
4′	
5′	SS
6′	SS
7′	5.92, m
8′	
9′	SS
10′	SS
11′	5.92, m
12′	
2-CH_3	2.08, s
8-CH_3	SS
4′-CH_3	2.41, s
8′-CH_3	2.42, s*
12a′-CH_3	2.40, s*
12b′-CH_3	2.49, s
6-OH	ND

Referenced to 75% CH_3CN/D_2O; * Signals interchangeable.

Figure S92. NMR data for compound eluting at 60.80 min (**15**) (*S. cf. fallax*).

© 2015 by the authors; licensee MDPI, Basel, Switzerland. This article is an open access article distributed under the terms and conditions of the Creative Commons Attribution license (http://creativecommons.org/licenses/by/4.0/).
